# On the aromaticity and photophysics of 1-arylbenzo[*a*]imidazo[5,1,2-*cd*]indolizines as bicolor fluorescent molecules for barium tagging in the study of double-beta decay of ^136^Xe

**DOI:** 10.3762/bjoc.21.126

**Published:** 2025-08-13

**Authors:** Eric Iván Velazco-Cabral, Fernando Auria-Luna, Juan Molina-Canteras, Miguel A Vázquez, Iván Rivilla, Fernando P Cossío

**Affiliations:** 1 Departamento de Química Orgánica I and Centro de Innovación y Química Avanzada (ORFEO-CINQA), Facultad de Química/Kimika Fakultatea, Universidad del País Vasco/Euskal Herriko Unibertsitatea (UPV/EHU), 20018 Donostia/San Sebastián, Spain; 2 Departmento de Química, Universidad de Guanajuato, 36050 Guanajuato, Gto, Mexico; 3 Donostia International Physics Center (DIPC), 20018 Donostia/San Sebastián, Spain; 4 Ikerbasque, Basque Foundation for Science, 48009 Bilbao, Spain

**Keywords:** aromaticity, DFT-TDDFT calculations, double-beta decay, fluorescent sensors, polycyclic arenes

## Abstract

In this paper, the behavior of a bicolor fluorescent indicator for the detection of barium cations formed by double-beta decay of ^136^Xe is analyzed by means of computational tools. Both DFT and TDDFT permit to understand the origin of the bicolor fluorescent signal emitted by 1-arylbenzo[*a*]imidazo[5,1,2-*cd*]indolizines in the free and Ba^2+^-bound states. The aromatic character of the fluorophore is analyzed by means of energetic (hyperhomodesmotic equations), structural (harmonic oscillator model of aromaticity, HOMA) and magnetic (nucleus independent chemical shifts, NICS) criteria. It is concluded that the aromatic character of the fluorophore is better described as the combination of two aromatic subunits integrated in the polycyclic system. Different DFT functional are used to analyze the photochemical behavior of this family of sensors. It is concluded that PBE0 and M06 functionals describe better the excitation process in the free state, whereas interaction of the sensor with Ba^2+^ requires the M06L functional. TDDFT analysis of the emission spectra shows larger errors, which have been corrected by means of a structural model. The bicolor behavior is rationalized based on the decoupling between the *para*-phenylene and benzo[*a*]imidazo[5,1,2-*cd*]indolizine components that results in a blue shift upon Ba^2+^ coordination.

## Introduction

**Figure 1:**
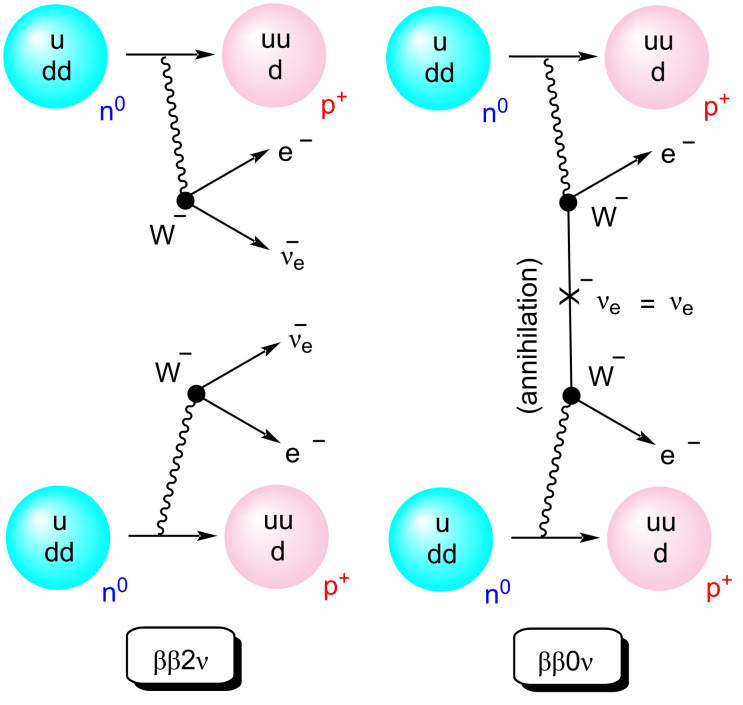
Two possible double beta decay modes. Left: with emission of two electronic antineutrinos (ββ2ν). Right: neutrinoless double beta decay (ββ0ν).

Double beta-decay [1] is a radioactive decay in which two neutrons are converted into two protons by means of the transformation of two quarks *down* into two quarks *up* (Figure 1). This process involves the emission of two *W^−^* bosons that in turn evolve towards the emission of two electrons. In the two-neutrino double-beta decay (ββ2ν), two electronic antineutrinos are also produced. Another possibility corresponds to the neutrinoless double-beta decay [2] (ββ0ν). This latter process could take place if the electronic neutrino is a Majorana particle [3], namely, it coincides with its own antiparticle (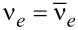). This would result in a mutual annihilation, according to which the two emitted electrons would take more energy than in the ββ2ν process. In both processes, the initial nuclide must advance two steps beyond the periodic table. Among the possible candidates for double-beta decay, ^136^Xe is a suitable isotope. In the ββ2ν radioactive decay, the reaction is ^136^Xe → ^136^Ba^2+^ + 2e^−^ + 2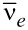. The ββ0ν analog would consist of simply ^136^Xe → ^136^Ba^2+^ + 2e^−^. Both transformations are extraordinarily rare events. For instance, the estimated half-life for the ββ0ν decay is at least higher than 2.3·10^26^ years, whereas the current best estimate of the age of the universe [4] is 13.8·10^9^ years. However, characterization of the neutrino as a Majorana particle constitutes a formidable challenge that would have an extraordinary impact in cosmology since this would contribute decisively to explain why our universe is formed by matter and not antimatter [5].

Within this context, from a chemical point of view, detection of ββ0ν radioactive decay of ^136^Xe requires an extremely sensitive detection of ^136^Ba^2+^. One promising candidate [6] would be a radiometric fluorescent sensor. With this idea in mind, we started a project aiming at designing, synthetizing and validating a fluorescent indicator that would fulfill the following conditions: (i) high discrimination between the free and Ba^2+^-bound states; (ii) high binding affinity for Ba^2+^, and low background signal for the chelated state. We reasoned that a bicolor fluorescent indicator [7] (FBI), namely, a radiometric sensor that emit the fluorescent signal at different wavelengths in the free and bound states, would be the best option given the extremely rare character of the ββ0ν event.

**Figure 2:**
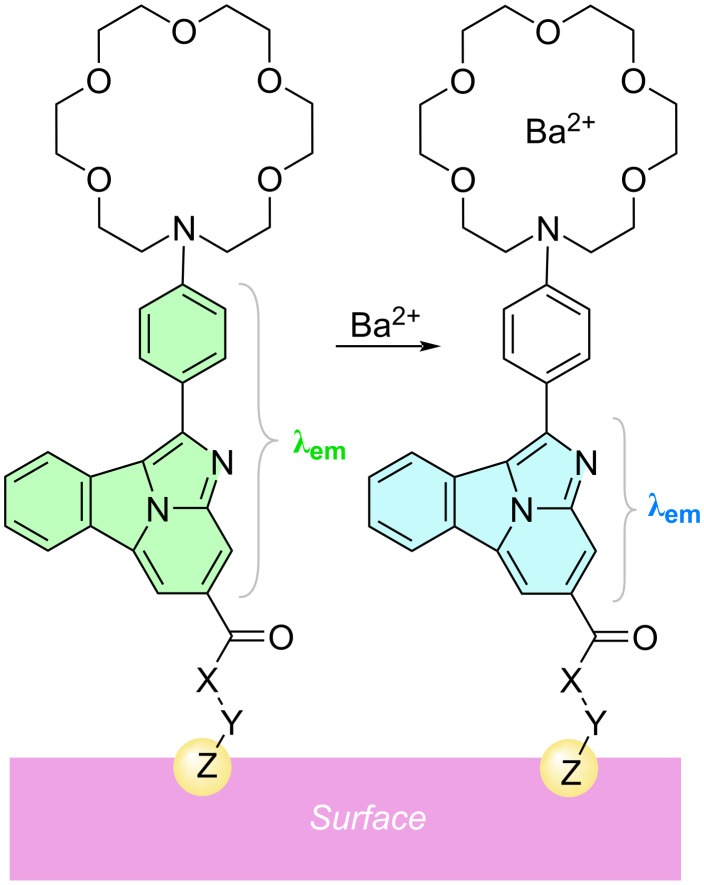
General structure of first-generation bicolor fluorescent indicators based on 1-aryl benzo[*a*]imidazo[5,1,2-*cd*]indolizines. X and Y represent the spacer and Z stands for the linker to the surface, respectively. The different emission wavelengths in the free and bound states are highlighted.

After analyzing different possibilities, we finally observed that FBIs based on benzo[*a*]imidazo[5,1,2-*cd*]indolizines as fluorescent moieties constitute promising candidates to detect Ba^2+^ cations [8–9] (Figure 2). Another essential component is an aza-crown ether of appropriate dimensions to capture the barium cation. In addition, one *para*-disubstituted phenyl (or aryl) group is installed to generate selective cation–π interactions. Finally, a spacer (denoted as X and Y in Figure 2) and a linker (denoted as Z) to anchor the sensor to a suitable surface via a covalent interaction are required. Ideally, different configurations and conformations of the fluorophore in the free and chelated states would result in a bicolor behavior in the emission spectra. Indeed, initial experiments were successful. However, we observed that translation of the behavior of these FBIs from supramolecular chemistry to solid–gas interfaces raises important issues in terms of both discrimination between free and chelated states and photophysical properties [10].

Therefore, in this paper, we reexamine the electronic features of these 1-arylbenzo[*a*]imidazo[5,1,2-*cd*]indolizine-based FBIs in terms of aromaticity (a relevant feature to analyze the nature of the excited states) and emission properties. The final goal of this research has been to contribute to the design of a second generation of bicolor fluorescent indicators for barium tagging in neutrinoless double-beta decay.

## Results and Discussion

**Scheme 1:**
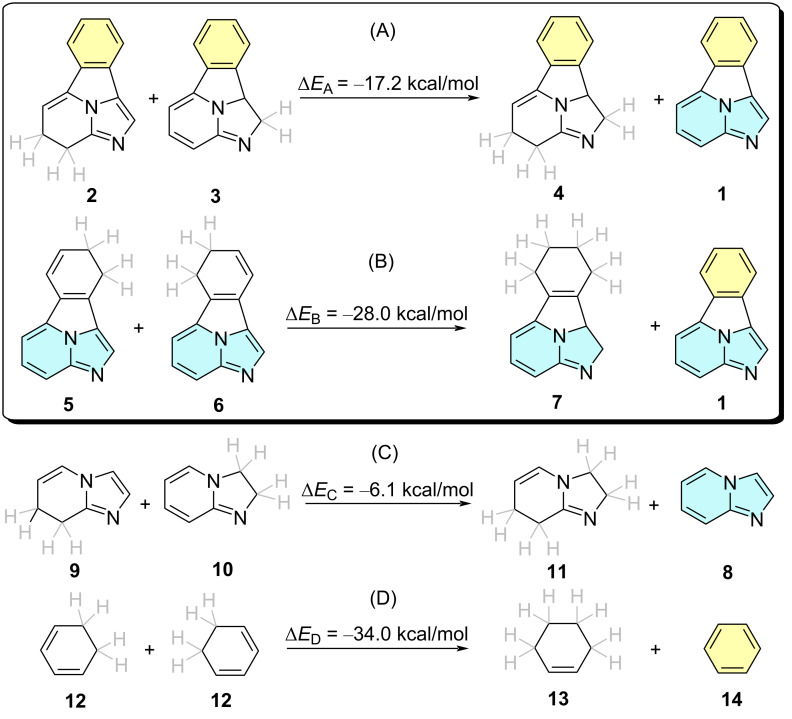
Hyperhomodesmotic equations used to analyze the resonance energy of benzo[*a*]imidazo[5,1,2-*cd*]indolizine **1** with respect to the imidazo[1,2-*a*]pyridine unit (A) and the *ortho*-disubstituted phenyl ring (B). Similar reactions for the separate components of **1** are shown in (C) and (D). All the relative energies have been calculated at the B3LYP-D3BJ/6-311+G** level of theory. Explicit hydrogens on the saturated C_sp³_ atoms are highlighted in gray.

First, we analyzed the aromaticity of parent benzo[*a*]imidazo[5,1,2-*cd*]indolizine **1** (Scheme 1) in order to get a better understanding of the properties of this tetracyclic system [11]. Since ground state aromaticity can be assessed by energetic [12], geometric [13] and magnetic [14–15] criteria, among others [16–18], we analyzed first the resonance energy of **1** with respect to the aromatic resonance energies of the *ortho-*phenyl and the bicyclic imidazo[1,2-*a*]pyridine components. In reaction A, an hyperhomodesmotic equation [19] **2** + **3** → **4** + **1** was defined, in which the conjugation of the bicyclic imidazo[1,2-*a*]pyridine unit was removed, while preserving the *ortho*-disubstituted phenyl ring, highlighted in yellow in Scheme 1A. This reaction yielded a stabilization energy of ca. 17 kcal/mol. In the alternative hyperhomodesmotic reaction B, defined as **5** + **6** → **7** + **1**, the formal ten-electron Hückel aromaticity of the imidazo[1,2-*a*]pyridine moiety (in blue) was preserved while the phenyl component was decomposed. The computed stabilization energy of this second reaction was calculated to be of 28 kcal/mol, slightly lower than the aromatic stabilization energy (ASE) and isomerization stabilization energy (ISE) calculated for benzene [20] (see reaction D in Scheme 1). Most likely this lowering stems from the strain imposed to the *ortho*-phenylene moiety in the tetracyclic structure. Combination of reactions A and B in the form


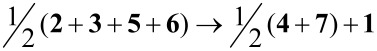


yields an average value of ⟨Δ*E*_AB_⟩ = −22.6 kcal/mol. A similar treatment of the separate components as outlined in reactions C and D shows a much lower stabilization energy for imidazo[1,2-*a*]pyridine **8** and a higher stabilization energy of benzene (**15**). Combination of these latter equations yields


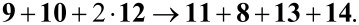


This second averaged equation results in a computed stabilization energy of ⟨Δ*E*_CD_⟩ = −20.5 kcal/mol, 2.1 kcal/mol lower than that calculated for ⟨Δ*E*_AB_⟩. These results indicate that there is a noticeable interplay between the phenyl (yellow) and imidazo[1,2-*a*]pyridine (blue) components of **1** and that these aromatic units preserve their respective aromatic characters.

**Figure 3:**
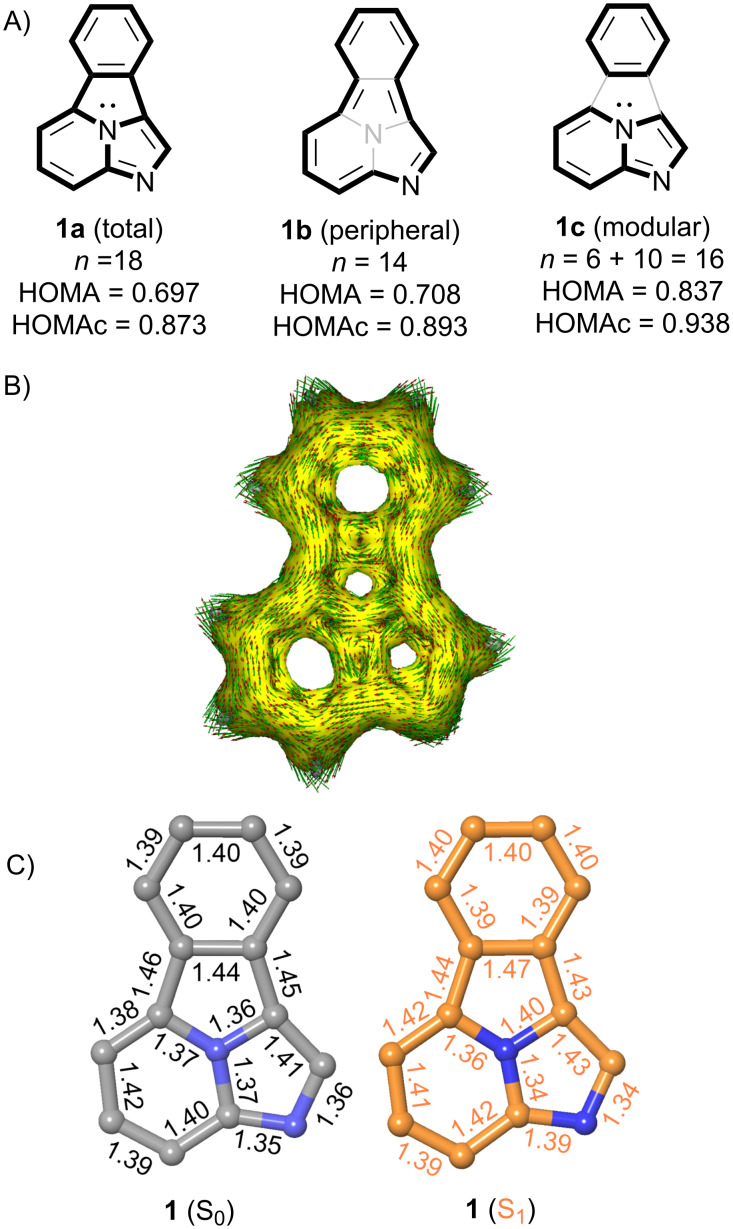
(A) Total, peripheral and modular delocalization patterns for fluorophore **1**. The ground state (S_o_) Harmonic oscillator models of aromaticity, both standard (HOMA) and computational antiaromaticity-including (HOMAc) descriptors are gathered for each pattern. Descriptor *n* stands for the number of covalent bonds, of each pattern, according to Equation 1. (B) Anisotropy of the current induced density (ACID) diagram of compound **1**. (isosurface value: 0.035 a.u.) (C) Bond distances (in Å) for fully optimized structure of **1**, computed at the ground state (S_0_) and at the first singlet excited state (S_1_, in orange). Results obtained at the B3LYP-D3BJ/6-311+G** (S_0_) and using TDDFT (S_1_). Hydrogen atoms have been omitted for clarity.

We next examined the aromatic character of benzo[*a*]imidazo[5,1,2-*cd*]indolizine **1** by analyzing its geometry in terms of bond equalization. Three possibilities were considered: a total delocalized geometry denoted as **1a** in Figure 3A, a peripheric conjugation **1b** that excludes the participation of the lone pair of the central N atom and, finally, a two-component delocalization scheme denoted as **1c**. The chief features of fully optimized structures of **1** at the ground state (S_0_) and first singlet excited state (S_1_) are reported in Figure 3B. Using geometric criteria, we computed the HOMA [21–22] for **1** at the ground state, according to the following expression:


[1] $$\text{HOMA}=1-\frac{1}{n}\sum_{i=1}^{n}\alpha_{\text{k}}{\left(R_{\text{k}}^{\text{opt}}-R_{\text{k}}^{\text{i}}\right)}^{2}.$$

In this equation, *n* is the number of covalent bonds, *k* describes the type of bond (CC or CN), 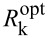 stands for the optimal CC or CN distances associated with aromatic structures, 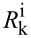 represents the corresponding bond distance gathered in Figure 3B, and α_k_ is a parametric term defined as


[2] $$\alpha_{k}=\frac{2}{{\left(R_{\text{k}}^{\text{s}}-R_{\text{k}}^{\text{d}}\right)}^{2}+{\left(R_{\text{k}}^{\text{d}}-R_{\text{k}}^{\text{opt}}\right)}^{2}},$$

where 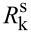 is the standard single bond distance of the *k-*pair of atoms (C–C, C–N) and 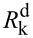 is the same paremeter but referred to the corresponding double bonds (C=C, C=N).

We computed the HOMA values associated with the total, peripheral and modular patterns using the standard parameters and a more recent set based on computational parameters that take into account antiaromaticity, denoted as HOMAc [23]. According to our results, formal structure **1a** is the less aromatic one, a result compatible with the formal anti-Hückel character of this structure, with 16 π-electrons if the central nitrogen atom is included in the electron counting. Peripheral structure **1b** is formally Hückel aromatic since the lone pair of this atom is not considered, thus resulting in 14 π-electrons and a higher HOMA value. Finally, modular structure **1c** includes formally separated components with six and ten π-electrons, both units being Hückel aromatic. This structure shows the highest HOMA and HOMAc values, which is in agreement with our conclusion from the analysis in terms of stabilization energy. In addition, an analysis of the Anisotropy of the induced current density (ACID) plot [24] calculated for **1** (Figure 3B) shows a significant diatropic ring current formally associated with the peripheral model **1b**. Another diatropic contribution can be assigned to the modular model **1c**, with a vortex in the bond connecting the phenyl group with the pyrrole ring. Interestingly, paratropic ring currents are observed close to the molecular plane.

This conclusion is reinforced by the NICS computed for the four rings of **1**. As shown in Table 1, the isotropic NICS values at the molecular plane are always negative, but the pyrrole ring shows the lowest value. Indeed, if the NICSzz(0) values are considered, a paratropic character is observed at the center of the pyrrole and imidazole rings. The situation is more consistent when the NICS values are computed 1 Å above the molecular plane [25–26] since diatropic ring currents are observed over the centers of the four ring points of electron density. However, the issues associated with magnetic criteria to describe the aromaticity of polycyclic systems must be taken into account. Thus, a recent study [27] emphasizes the relative (and competitive) contributions of global, semi-local and local ring currents associated with Kekulé resonance and Clar’s disjoint aromatic π–sextets, which reveals different coexisting ring current circuits in this kind of systems. Therefore, the assessment of the aromaticity of system **1** relies on the combined agreement among conceptually different criteria. In summary, thermochemical, structural and magnetic analysis permit to conclude that the aromaticity of the fluorophore defined by **1** has modular and peripheral character, which results in a moderate total aromaticity for this parent compound in the ground state. Since it is known that the aromaticity rules are reversed in ^1^ππ* excited states [28], the high fluorescent response of **1** is connected with its higher aromaticity at the excited S_1_ state. Actually, the two peripheric C–C bonds of the central pyrrole ring are slightly shorter in the optimized S_1_ state, thus suggesting a less modular aromatic character. Unfortunately, since the HOMA parameters for excited states [29] are available for triplet ^3^ππ* states only and we are interested in fluorescence emission spectra, this kind of quantitative assessment of aromaticity was not possible.

**Table 1:** NICS(iso) and NICSzz values at the molecular plane (*z* = 0) and 1 Å (*z* = 1) above this plane in a perpendicular direction. Points *a–c* correspond to the respective ring points, in light red. Perpendicular points at *z* = 1 are shown in light green.

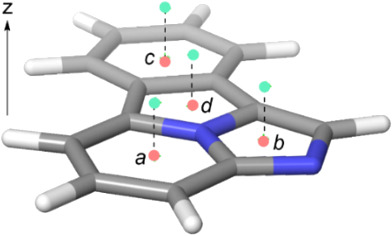				

Point	NICS(iso)^a^		NICSzz^a^	

	*z* = 0	*z* = 1	*z* = 0	*z* = 1

*a*	−11.729	−16.728	−11.798	−32.255
*b*	−12.486	−11.486	+8.812	−31.343
*c*	−8.740	−10.298	−12.648	−27.974
*d*	−7.287	−7.705	+8.730	−17.884

^a^Values computed using the B3LYP/6-311+G** hybrid functional and the GIAO method.

**Scheme 2:**
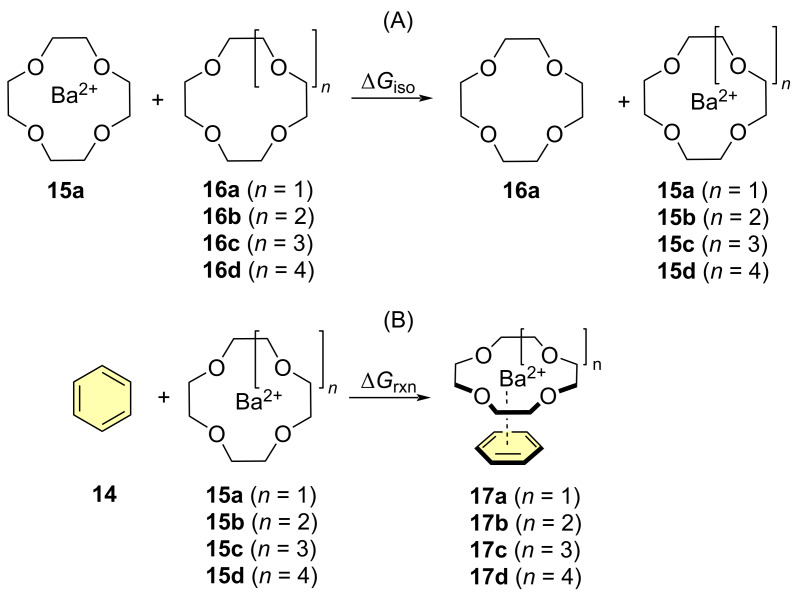
Isodesmic (A) and reaction profiles (B) for the analysis of the interaction of Ba^2+^ with different crown ethers and a benzene ring as a computational model of *para*-phenylene ring shown in Figure 2.

The role of the crown ether and the *para*-phenylene moieties was also analyzed. The interactions of different sized crown ethers with Ba^2+^ and coordination with the aromatic ring modeled by means of benzene (**14**, highlighted in yellow in Scheme 2) were studied computationally. Although the efficiency of crown ethers as components in cation-selective fluorescent probes has been extensively explored [7,30], to the best of our knowledge no previous computational DFT studies on the selectivity of crown ethers of different sizes with Ba^2+^ have been reported. Therefore, we explored (Scheme 2A) the binding between this cation and 12-crown-4 (**16a**, *n* = 1), 15-crown-5 (**16b**, *n* = 2), 18-crown-6 (**16c**, *n* = 3) and 21-crown-7 (**16d**, *n* = 4), to form Ba^2+^·crown ethers **15a–d**. We compared the corresponding binding energies by means of the following isodesmic equation:


[3] $$\begin{array}{r} \Delta G_{iso}(\text{a}-\text{d})=\Delta G_{298}(\text{15a}-\text{d})+\Delta G_{298}(\text{16a})-\\ {}[\Delta G_{298}(\text{16a}-\text{d})+\Delta G_{298}(\text{15a})]. \end{array}$$

Where the different terms correspond to Gibbs energies computed at 298.17 K. We also extended this study to the interaction between complexes **15a–d** and benzene (**14**) and computed the corresponding complexation energies as


[4] $$\begin{array}{c} \Delta G_{\mathrm{rxn}}(\text{a}-\text{d})=\Delta G_{298}(\text{17a}-\text{d})-\\ {}[\Delta G_{298}(\text{14})+\Delta G_{298}(\text{15a}-\text{d})]. \end{array}$$

**Figure 4:**
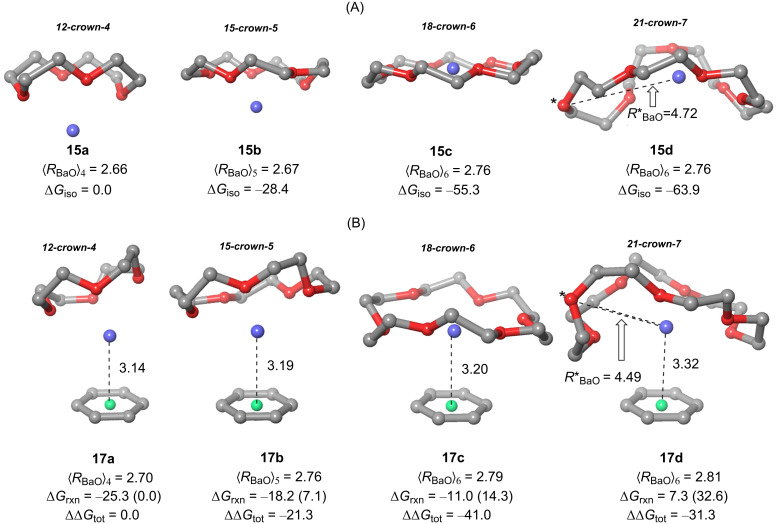
Fully optimized geometries (B3LYP-D3BJ/6ccrow-311++G**&DefTZVPP(Ba) level of theory) of Ba^2+^·crown ethers **15a–d** (A) and phenyl·Ba^2+^·crown ether complexes **17a–d** (B). Barium cations are represented in dark blue. Descriptors (⟨*R*_Ba0_⟩)*_n_* denote the average Ba–O bond distances (in Å) for the different crown ethers. Distances between Ba^2+^ and the ring points of electron density of benzene (in green) are also gathered in (B). Δ*G*_iso_ and Δ*G*_rxn_ terms stand for the Gibbs energies (in kcal/mol) described in Scheme 2 and have been calculated according to Equation 3 and Equation 4. Numbers in parentheses are the relative Δ*G*_rxn_ energies with respect to complex **17a**. ΔΔ*G*_tot_ energies have been calculated according to Equation 5. Hydrogen atoms have been omitted for clarity.

In addition, Figure 4 includes the chief geometric parameters of the different complexes, as well as the corresponding free energy values.

Our calculations show that, as expected, 12-crown-4 **16a** and 15-crown-5 **16b** are too small and consequently the barium cation lies outside the average molecular plane determined by the macrocycle. In the case of 18-crown-6 **16c**, the cyclic ligand accommodates very well the cation, which is now within the average molecular plane. In addition, the corresponding Δ*G*_iso_ values increase with the *n*-values (Figure 4A). Ligand 21-crown-7 **15d** suggests that this size of the cyclic ligand is less than optimal, since the calculated structure shown a concave-convex topology, in which one oxygen atom, highlighted by an asterisk, lies out from the direct coordination perimeter, thus suggesting that this ligand is too big. The relatively lower increase of the Δ*G*_iso_(**d**) with respect to its Δ*G*_iso_(**c**) congener also indicate that the stabilization induced by the additional oxygen atom is lower in magnitude.

An analysis of the effect of the aromatic ring represented by the benzene ring shown in Scheme 2 and in Figure 4B was also performed. We observed that for complexes **17a** (*n* = 1) and **17b** (*n* = 2) the low size of the crown ethers generates a poor coordination to Ba^2+^, which results in more charge available for further coordination thus giving rise to a relatively strong π–cation interaction with the phenyl group. In the case of complex **17c** (*n* = 3) stemming from 18-crown-6, the barium cation remains within the average molecular plane determined by the macrocyclic moiety. The larger Δ*G*_iso_ value for **15c** results in a relatively lower Δ*G*_rxn_ free energy for **17c**, given the lower charge available for further interaction with the phenyl group. The geometry of complex **17d** (*n* = 4) resembles that found for parent **15d**, since the 21-crown-7 moiety adopts a concave–convex shape, in which the barium cation occupies a central position within the concave face. Also in this case, one oxygen atom of the oversized macrocycle does not interact directly with Ba^2+^, thus resulting in a non-optimal coordination pattern. Therefore, the shape of the ligand and the low positive charge available for the cation result in the largest Ba^2+^-ring point distance and in a positive value of Δ*G*_rxn_(**d**) although the corresponding energy is slightly negative (ca. −4 kcal/mol). If we combine both relative magnitudes in the form


[5] $$\Delta \Delta G_{tot}(\text{a}-\text{d})=\Delta G_{iso}(\text{a}-\text{d})-[\Delta G_{\mathrm{rxn}}(\text{a}-\text{d})-\Delta G_{\mathrm{rxn}}(\text{a})]$$

in which the second term of the right hand (in brackets) correspond to the relative Gibbs reaction energy with respect to **17a**, gathered in parentheses in Figure 4. These combined ΔΔ*G*_tot_ values permit to conclude that 18-crown-6 (*n* = 3) is the best tradeoff between coordination to the cation and subsequent interaction with the phenyl group. This is the reason why in our design we introduced and aza-equivalent of 18-crown-6, namely the 1,4,7,10,13-pentaoxa-16-azacyclooctadecane moiety.

**Figure 5:**
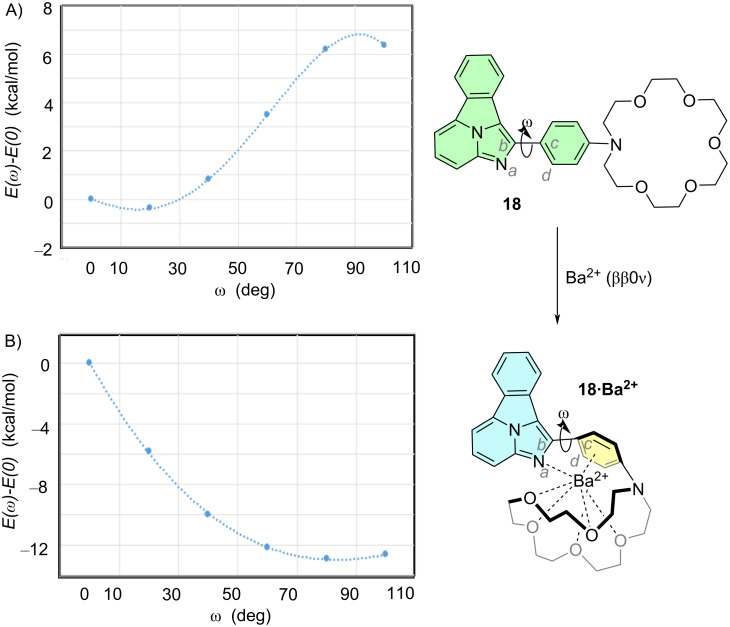
Relaxed scan of the relative conformational energies of sensor **18** at the free state (A) and bound to barium cation **18·Ba^2+^** (B), calculated at the B3LYP-D3BJ/6-31G*&DefTZVPP(Ba) level of theory. Capture of one naked barium cation generated after a neutrinoless double-beta decay (ββ0ν) is assumed. The dihedral angle ω = *a–b–c–d* formed by the two components of the fluorophore are graphically defined.

We next investigated the coupling between the two components of the sensor gathered in Figure 2 at the free and Ba^2+^-bound states, namely the aza-crown ether-Ba^2+^-*para*-phenylene and the benzo[*a*]imidazo[5,1,2-*cd*]indolizine components. We chose compound **18** (Figure 5) as a convenient computational model. We calculated the energy profile associated with the rotation between the 1,4-phenylene and benzo[*a*]imidazo[5,1,2-*cd*]indolizine **1** components, defined as variation of the ω = *a*–*d*–*c*–*d* dihedral angle shown in Figure 5. Our calculations show that, in the absence of barium, compound **18** exhibits almost coplanar components, so both systems form a combined fluorophore highlighted in green in Figure 5. The correlation between energy and this dihedral angle by means of a Karplus-like [31] equation up to the fourth degree in the form


[6] $$\begin{array}{c} E(\omega )-E(0)=13.89{\text{cos}}^{4}\omega -10.61{\text{cos}}^{3}\omega -\\ 9.73{\text{cos}}^{2}\omega -0.20\text{cos}\omega +6.55 \end{array}$$

shows an excellent correlation (R^2^ = 0.9987). The situation is completely different in the presence of a naked barium cation (Figure 5B). Thus, the *E*(ω) – *E*(0) vs ω curve shows a wide minimum in the region of 90 deg. Also in this case, the correlation for a fourth-degree polynomial expansion in terms of cos*^n^*ω in the form


[7] $$\begin{array}{c} E(\omega )-E(0)=84.56{\text{cos}}^{4}\omega -113.49{\text{cos}}^{3}\omega -\\ 40.10{\text{cos}}^{2}\omega -2.32\text{cos}\omega -14.12 \end{array}$$

with a correlation factor of R^2^ = 0.9829. This minimum involves the simultaneous coordination of the cation to one nitrogen atom of the fluorophore **1**, to the *para*-phenylene group and the crown ether, a result in line with our experimental results [8].

**Scheme 3:**
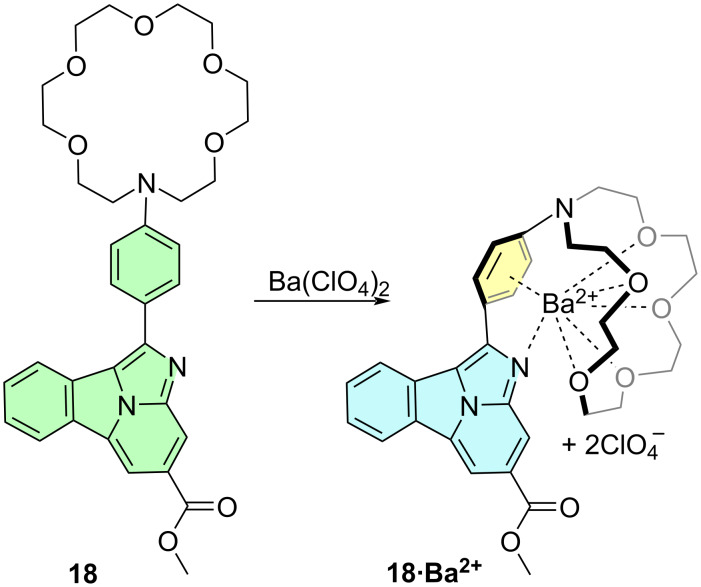
Reaction of fluorescent probe **18** with barium perchlorate, as indicated in Figure 2 (X = O, Y, Z = Me). The possible coordination patterns are shown.

**Figure 6:**
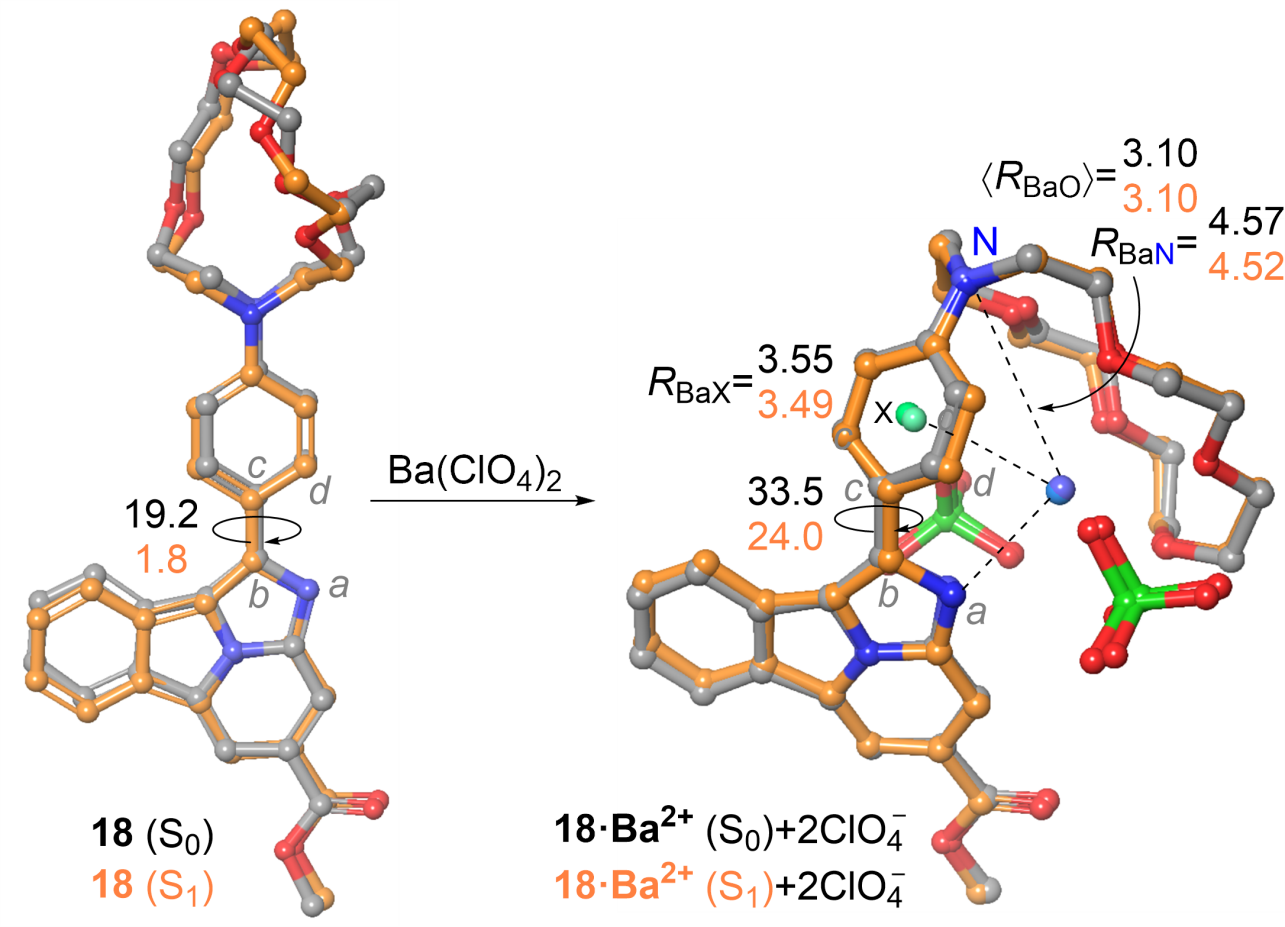
Fully optimized structures (B3LYP-D3BJ/6-311++G(d,p)&DefTZVPP(Ba) level of theory) of compounds **18** and **18·Ba^2+^** at the ground (S_0_, carbon atoms in gray) and first excited (S_1_, carbons in orange) sates. Bond distances are given in Å. The dihedral angles ω = *a–b–c–d*, in absolute value, are reported in deg.

Next, we analyzed the geometry and electronic features of synthetic compounds **18** and **18·Ba^2+^** as a model case study of the general design shown in Figure 2. Instead of the isolated cation generated by the ββ0ν process, we included barium perchlorate since this salt was used in experimental studies, as it can be observed in Scheme 3. DFT and TDDFT calculations show that the geometries of **18** at the ground first excited states are quite similar (Figure 6), the aza-crown ether component being more flexible, in good agreement with our experimental observations [9], with very low values of the dihedral angle formed by the benzo[*a*]imidazo[5,1,2-*cd*]indolizine and the *para*-phenylene groups, especially in the S_1_ state, thus indicating that both aromatic units are coupled under excitation–relaxation to produce the corresponding absorption–emission spectra (vide infra). The calculated structures of **18** complexed with barium perchlorate are more rigid, with only small modifications on going from the ground state to the first single excited state (Figure 6). However, the presence of the two perchlorate anions results in additional coordination with Ba^2+^, thus resulting in larger values of the Ba–N distances, as well as of the average Ba–O and Ba–phenylene distances. In addition, the ω = *a–b–c–d* dihedral angle between fluorophore **1** and the 1,4-phenylene ring is smaller than that calculated for the naked barium cation, but still shows a noticeable departure from coplanarity.

**Figure 7:**
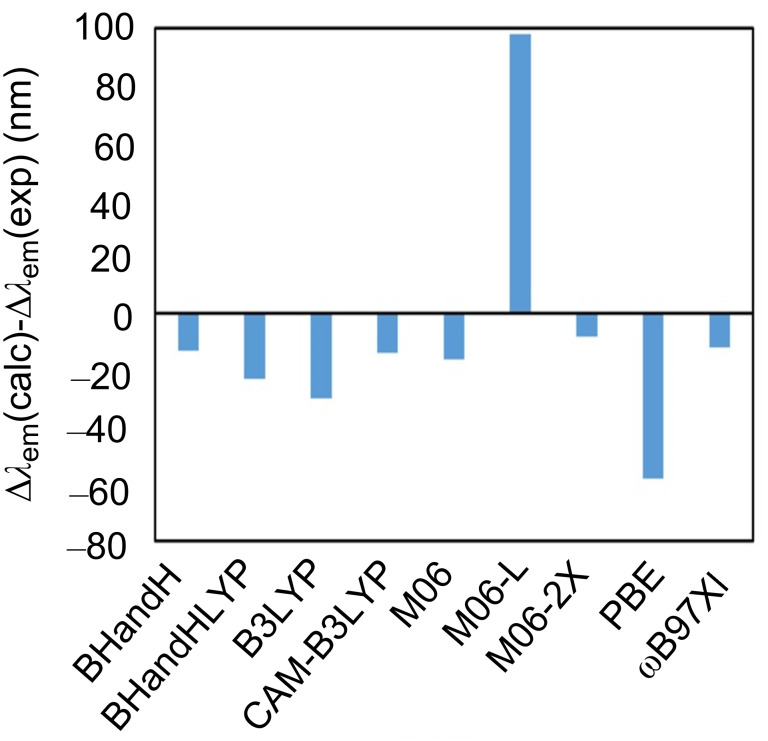
Comparison between the calculated and experimental differences between the emission wavelength of **18** and **18·Ba(ClO_4_)_2_**, with different functionals within DFT and TDDFT frameworks.

The peculiar behavior of barium perchlorate with respect to naked Ba^2+^ prompted us to compare the photophysical properties of unbound compound **18** in the presence of Ba(ClO_4_)_2_. The values corresponding to the adiabatic absorption (*S*_0_(optimized) → *S*_1_*, adiabatic absorption) and emission (*S*_1_(optimized) → *S*_0_, fluorescence) are reported in Table 2, together with the differences between the free and bound states. The corresponding signed errors are gathered in Figure 7.

**Table 2:** Calculated^a^ absorption (λ_abs_, in nm) and emission (λ_em_, in nm) wavelengths of compound **19** at the free and barium perchlorate bound states, using different DFT functionals.

Functional	λ_abs_				λ_em_		
	
	**18**	**18·Ba(ClO** **_4_** **)** **_2_**	Δλ_abs_^b^		**18**	**18·Ba(ClO** **_4_** **)** **_2_**	Δλ_em_^b^

experimental^c^	434	420	−14		508	434	−74
BHandH	377	335	−42		425	364	−61
BHandHLYP	376	335	−41		427	376	−51
B3LYP	462	397	−36		462	426	−36
CAM-B3LYP	382	342	−40		435	375	−60
M06	431	382	−49		478	420	−58
M06-L	384	341	−43		621	645	+24
M06-2X	518	432	−86		445	379	−66
PBE	433	369	−64		473	457	−16
ωB97XD	377	340	−37		434	372	−62

^a^Calculations performed with the 6-311++G(d,p)& DefTZVPP (Ba) basis sets and effective-core potential. ^b^Difference between the free and chelated values: Δλ = λ(**19·Ba(ClO_4_)_2_**) − λ(**19**). ^c^Data taken from ref. [8].

The behavior of the different functionals resulted to be very variable, although the ground state and excited state geometries were very similar. In the case of absorption wavelengths, wB97XD, BHandHHLYP and CAM-B3LYP were the most convenient functionals to describe the blue shift on going from the free to the Ba-chelated state. If errors for the free and chelated states are considered, B3LYP and ωB97XD are the functionals that introduce the lowest error values, although these data are less relevant than Δλ_abs_.

Calculated emission wavelengths and the differences between the calculated fluorescent emissions in the free and bound states showed in some cases noticeable differences. Thus, M06-L and wB97XD functionals described better the emission of **18** at the unbound state, whereas M06 and M06-L gave the lower errors for the λ_em_ values of **18·Ba(ClO_4_)_2_**. However, the situation was found to be different when the Δλ_em_ values were calculated. In this case, M06 (which even predicted a red shift) and PBE were the less accurate functionals, whereas M06-2X was the most precise functional, followed by wB97XD, the other functionals being quite similar among them. Therefore, we concluded that M06-2X, whose calculated geometry is almost coincident with that computed with B3LYP-D3BJ, is the most precise functional to predict the two-color behavior of these fluorescent sensors.

## Computational Methods

All the DFT [32] and TDDFT [33] calculations were performed using the B3LYP[34–36], B3LYP-D3BJ [37–38], CAM-B3LYP [39], M06 [40–41], M06-2X [42], M06-L [43–45], PBE0 [46] and ωB97XD [47] functionals. The 6-311+G* and 6-31++G** bases sets [48–49] were used for C, N, O, and H. The DefTZVPP [50] effective-core potential and basis set were used for Na and Ba. NICS calculations were carried out by using the GIAO [51] method. Wiberg bond orders [52] were computed within the NBO bicentric localized orbitals [53–54]. All structures were fully optimized [55] and characterized by harmonic analysis. All the calculations were performed by using the Gaussian 16 suite of programs [56].

## Conclusion

From the computational study reported in this paper, we conclude that the benzo[*a*]imidazo[5,1,2-*cd*]indolizine scaffold is a convenient fluorophore for barium tagging in neutrinoless double-beta decay. This fluorophore exhibits modular aromaticity in which the central pyrrole ring is less aromatic that the other three rings, as proved by energetic, geometric and magnetic criteria of aromaticity. The lower ground state aromaticity of the tetracyclic system, as a whole, results in a highly fluorescent signal in the first singlet excited state. Analysis of the crown-ether component permits to conclude that the aza-analog equivalent to 18-crown-6 represents the best compromise between coordinating oxygen atoms and ability to form a π–Ba^2+^ complex with the *para*-phenylene component of the sensor. Rotation about the dihedral angle defined by the two aromatic components of the sensor result in an essentially planar conformation at the free state, whereas binding to a naked barium cation results in a perpendicular arrangement between the benzo[*a*]imidazo[5,1,2-*cd*]indolizine and the 1,4-phenylene components, thus promoting a blue shift responsible for the bicolor behavior of the sensor. Interaction with barium perchlorate results in a slightly different coordination pattern, although the bicolor behavior observed in the experimental fluorescence spectra is preserved. These photophysical properties were observed in DFT and TDDFT calculations. Although the calculated geometries were found to be very similar, the emission wavelengths varied significantly depending upon the functional used.

These conclusions have permitted us to design a second generation of fluorescent bicolor sensors with modifications at the benzo[*a*]imidazo[5,1,2-*cd*]indolizine scaffold. The chemical synthesis, photophysical properties and suitability for barium tagging will be published in due course.

## Supporting Information

File 1 Energies, calculated absorption and emission wavelengths.

File 2 Cartesian coordinates of the optimized structures.

## Data Availability

All data that supports the findings of this study is available in the published article and/or the supporting information of this article.
