# Supplementary material for: On the aromaticity and photophysics of 1-arylbenzo[a]imidazo[5,1,2-cd]indolizines as bicolor fluorescent molecules for barium tagging in the study of double-beta decay of 136Xe
Source: Beilstein J Org Chem. 2025 Aug 13;21:1627–38. doi: 10.3762/bjoc.21.126 (PMC12362309; doi:10.3762/bjoc.21.126)
Supplement: File 1 — Energies, calculated absorption and emission wavelengths. [file Beilstein_J_Org_Chem-21-1627-s001.pdf]

BEILSTEIN JOURNAL OF ORGANIC CHEMISTRY

# Supporting Information

for

# On the aromaticity and photophysics of 1-arylbenzo[*a*]imidazo[5,1,2-*cd*]indolizines as bicolor fluorescent molecules for barium tagging in the study of double-beta decay of $^{136}Xe$

Eric Iván Velazco-Cabral, Fernando Auria-Luna, Juan Molina-Canteras, Miguel A. Vázquez, Iván Rivilla and Fernando P. Cossío

*Beilstein J. Org. Chem.* **2025,** *21,* 1627–1638. doi:10.3762/bjoc.21.126

## Energies, calculated absorption and emission wavelengths

License and Terms: This is a supporting information file under the terms of the Creative Commons Attribution License (https://creativecommons.org/licenses/by/4.0). Please note that the reuse, redistribution and reproduction in particular requires that the author(s) and source are credited and that individual graphics may be subject to special legal provisions.
The license is subject to the *Beilstein Journal of Organic Chemistry* terms and conditions: (https://www.beilstein-journals.org/bjoc/terms)

**Table S1**. Total energies (E), zero-point vibrational energies (ZPVE) and thermal corrections for Gibbs energies (TCGE) calculated for compounds **1-15**.[a]

| Compound | E (a.u) | ZPVE (a. u.) | TCGE (a.u.) |
|---|---|---|---|
| **1** ($S_0$) | -609.9060587 | 0.176762 | 0.141739 |
| **1** ($S_1$) | -609.8996567 | | |
| **2** | -611.0968762 | 0.199189 | 0.163241 |
| **3** | -611.0866456 | 0.199260 | 0.163686 |
| **4** | -612.3055856 | 0.222345 | 0.186106 |
| **5** | -611.090726 | 0.199031 | 0.163115 |
| **6** | -611.0904228 | 0.199084 | 0.163161 |
| **7** | -612.3213374 | 0.222925 | 0.186554 |
| **8** | -379.9757466 | 0.117380 | 0.087238 |
| **9** | -381.1757085 | 0.140157 | 0.109153 |
| **10** | -381.169404 | 0.139909 | 0.109333 |
| **11** | -382.3796935 | 0.163231 | 0.131183 |
| **12** | -233.5049978 | 0.121901 | 0.094336 |
| **13** | -234.7365585 | 0.145754 | 0.117773 |
| **14** | -232.330161 | 0.100194 | 0.075076 |

[a] Values computed at the B3LYP-D3BJ/6.311++G(d,p)%DefTZVPP(Ba) level.

**Table S2**. Total energies (E), zero-point vibrational energies (ZPVE) and thermal corrections for Gibbs energies (TCGE) calculated for compounds **15a-d, 16a-d, 17a-d**, **19** and **$\cdot Ba(ClO_4)_2$**.[a]

| Compound | E (a.u) | ZPVE (a. u.) | TCGE (a.u.) |
|---|---|---|---|
| **15a** | -640.6658645 | 0.248434 | 0.208982 |
| **15b** | -794.6007494 | 0.309634 | 0.264037 |
| **15c** | -948.533772 | 0.371660 | 0.320205 |
| **15d** | -1102.443735 | 0.433721 | 0.378415 |
| **16a** | -615.5355929 | 0.245247 | 0.207758 |
| **16b** | -769.4247609 | 0.306950 | 0.262335 |
| **16c** | -923.3163967 | 0.368607 | 0.320017 |
| **16d** | -1077.208118 | 0.429505 | 0.373651 |
| **17a** | -873.050199 | 0.349293 | 0.295361 |
| **17b** | -1026.9762566 | 0.410893 | 0.353034 |

| | | | |
|---|---|---|---|
| **17c** | -1180.8999491 | 0.472725 | 0.411279 |
| **17d** | -1334.7818278 | 0.534993 | 0.470533 |
| **19** ($S_0$) | -1971.2608099 | 0.661267 | 0.584192 |
| **19** ($S_1$) | -1971.1557349 | | |
| **19·Ba(ClO$_4$)$_2$** ($S_0$) | -3518.7683558 | 0.686340 | 0.591384 |
| **19·Ba(ClO$_4$)$_2$** ($S_1$) | -3518.6550761 | | |

[a] Values computed at the B3LYP-D3BJ/6.311++G(d,p)%DefTZVPP(Ba) level.

**Table S3**. Total energies calculated for compounds **19** and **19·Ba(ClO$_4$)$_2$** at $S_0$ and $S_1$ states with different functionals.[a]

| Functional | $S_0$ | | $S_1$ | |
|---|---|---|---|---|
| | **19** | **Ba(ClO$_4$)$_2$** | **19** | **Ba(ClO$_4$)$_2$** |
| BHandH | -1957.14463621 | -3498.81290051 | -1956.9999427 | -3498.6296551 |
| BHandHLYP | -1969.94831606 | -3516.90526237 | -1969.8128635 | -3516.7529069 |
| B3LYP | -1971.2608099 | -3518.76835587 | -1971.1557349 | -3518.6550761 |
| CAM-B3LYP | -1970.15492224 | -3517.34937615 | -1970.0276974 | -3517.2112303 |
| M06 | -1969.78065911 | -3516.87448682 | -1969.6462543 | -3516.7563090 |
| M06-L | -1970.84848954 | -3518.23281981 | -1969.6714904 | -3518.1215546 |
| M06-2X | -1970.33142837 | -3517.42930374 | -1970.2099690 | -3517.2858321 |
| PBE | -1968.84831481 | -3515.32524575 | -1968.7408013 | -3515.1955066 |
| wB97XD | -1970.47373002 | -3517.74326079 | -1970.3499872 | -3517.6037878 |

[a] Values computed with the 6.311++G(d,p)&DefTZVPP(Ba) basis set.
