# Supplementary material for: On the aromaticity and photophysics of 1-arylbenzo[a]imidazo[5,1,2-cd]indolizines as bicolor fluorescent molecules for barium tagging in the study of double-beta decay of 136Xe
Source: Beilstein J Org Chem. 2025 Aug 13;21:1627–38. doi: 10.3762/bjoc.21.126 (PMC12362309; doi:10.3762/bjoc.21.126)
Supplement: File 2 — Cartesian coordinates of the optimized structures. [file Beilstein_J_Org_Chem-21-1627-s002.pdf]

BEILSTEIN JOURNAL OF ORGANIC CHEMISTRY

Supporting Information

for

On the aromaticity and photophysics of 1-arylbenzo[*a*]imidazo[5,1,2-*cd*]indolizines as bicolor fluorescent molecules for barium tagging in the study of double-beta decay of ^{136}Xe

Eric Iván Velazco-Cabral, Fernando Auria-Luna, Juan Molina-Canteras, Miguel A. Vázquez, Iván Rivilla and Fernando P. Cossío

Beilstein J. Org. Chem. **2025,** *21,* 1627–1638. doi:10.3762/bjoc.21.126

Cartesian coordinates of the optimized structures

License and Terms: This is a supporting information file under the terms of the Creative Commons Attribution License (https://creativecommons.org/licenses/by/4.0). Please note that the reuse, redistribution and reproduction in particular requires that the author(s) and source are credited and that individual graphics may be subject to special legal provisions.
The license is subject to the *Beilstein Journal of Organic Chemistry* terms and conditions: (https://www.beilstein-journals.org/bjoc/terms)

Cartesian coordinates of all the stationary points discussed in the main text

1 (S_0)

Center Number	Atomic Number	Atomic Type	Coordinates (Angstroms) X	Y	Z
1	6	0	2.313815	2.199899	0.000000
2	6	0	1.163644	2.989069	0.000000
3	6	0	-0.082170	2.343002	-0.000000
4	7	0	0.000000	0.976132	-0.000000
5	6	0	1.078281	0.135525	0.000000
6	6	0	2.299495	0.780334	0.000000
7	7	0	-1.390766	2.686558	-0.000000
8	6	0	-2.090172	1.518370	-0.000000
9	6	0	-1.236701	0.398700	-0.000000
10	6	0	0.470554	-1.188346	0.000000
11	6	0	-0.962957	-1.023604	-0.000000
12	6	0	-1.787372	-2.151083	-0.000000
13	6	0	-1.201987	-3.411203	-0.000000
14	6	0	0.193169	-3.569839	0.000000
15	6	0	1.034407	-2.464391	0.000000
16	1	0	3.276797	2.695792	0.000000
17	1	0	1.220977	4.068804	-0.000000
18	1	0	3.227602	0.225334	0.000000
19	1	0	-3.169530	1.526633	-0.000000
20	1	0	-2.865400	-2.044957	-0.000000
21	1	0	-1.835019	-4.290818	-0.000000
22	1	0	0.617157	-4.566654	0.000000
23	1	0	2.110737	-2.591556	0.000000

1 (S_1)

Center Number	Atomic Number	Atomic Type	Coordinates (Angstroms) X	Y	Z
1	6	0	2.269672	2.271280	0.000000
2	6	0	1.090943	3.045375	-0.000000
3	6	0	-0.128685	2.317132	-0.000000
4	7	0	0.000000	0.986054	-0.000000
5	6	0	1.086460	0.161220	0.000000
6	6	0	2.321799	0.866821	0.000000
7	7	0	-1.483437	2.612683	-0.000000
8	6	0	-2.152273	1.448329	-0.000000
9	6	0	-1.244906	0.343410	-0.000000
10	6	0	0.521343	-1.160690	0.000000
11	6	0	-0.942584	-1.049966	-0.000000
12	6	0	-1.746055	-2.188483	-0.000000
13	6	0	-1.140056	-3.453175	0.000000
14	6	0	0.256477	-3.559238	0.000000
15	6	0	1.094482	-2.431997	0.000000
16	1	0	3.214578	2.803749	0.000000
17	1	0	1.107755	4.123410	-0.000000
18	1	0	3.267700	0.346361	0.000000
19	1	0	-3.230091	1.416707	-0.000000
20	1	0	-2.825657	-2.095135	-0.000000
21	1	0	-1.750117	-4.346723	0.000000
22	1	0	0.709587	-4.544079	0.000000
23	1	0	2.170606	-2.555543	0.000000

2

Center Number	Atomic Number	Atomic Type	Coordinates (Angstroms) X	Y	Z
1	6	0	2.652359	-1.788447	-0.238304
2	6	0	3.189395	-0.416717	0.273562
3	6	0	2.191741	0.674667	0.027304
4	7	0	0.913866	0.258206	-0.077769
5	6	0	0.327442	-1.009882	-0.002331
6	6	0	1.167281	-2.053911	-0.020053
7	7	0	2.222661	1.997933	0.045604
8	6	0	0.896538	2.419786	-0.030178
9	6	0	0.048994	1.326120	-0.090234
10	6	0	-1.116487	-0.702186	0.022694
11	6	0	-1.282476	0.718559	-0.049866
12	6	0	-2.559655	1.265847	-0.055552
13	6	0	-3.661778	0.410225	0.009492
14	6	0	-3.498062	-0.974546	0.076883
15	6	0	-2.220450	-1.539518	0.081256
16	1	0	2.837325	-1.853562	-1.320180
17	1	0	3.243973	-2.586727	0.214891
18	1	0	4.149406	-0.194076	-0.193910
19	1	0	3.367566	-0.470208	1.355392
20	1	0	0.809077	-3.074335	0.016729
21	1	0	0.653108	3.469589	-0.031775
22	1	0	-2.699377	2.338667	-0.110328
23	1	0	-4.661193	0.829332	0.006244
24	1	0	-4.369074	-1.616699	0.126472
25	1	0	-2.095551	-2.614930	0.133577

3

Center Number	Atomic Number	Atomic Type	Coordinates (Angstroms) X	Y	Z
1	6	0	2.604477	-1.838988	-0.110322
2	6	0	3.134131	-0.577777	-0.134364
3	6	0	2.227311	0.543335	-0.015627
4	7	0	1.002189	0.179081	0.556918
5	6	0	0.389686	-1.021864	0.275220
6	6	0	1.192397	-2.092782	0.004162
7	7	0	2.287023	1.760975	-0.432826
8	6	0	0.912199	2.321542	-0.338246
9	6	0	0.104090	1.333832	0.563111
10	6	0	-1.039619	-0.722075	0.147729
11	6	0	-1.212378	0.680437	0.210911
12	6	0	-2.470720	1.239166	0.045375
13	6	0	-3.554945	0.394544	-0.205174
14	6	0	-3.383503	-0.991018	-0.268136
15	6	0	-2.124627	-1.561411	-0.091456
16	1	0	3.264247	-2.683334	-0.277569
17	1	0	4.174858	-0.398580	-0.366297
18	1	0	0.783256	-3.070152	-0.206847
19	1	0	0.930353	3.319361	0.100048
20	1	0	0.495188	2.404770	-1.349315
21	1	0	0.044415	1.739001	1.581143
22	1	0	-2.613535	2.312377	0.101313
23	1	0	-4.541624	0.817979	-0.351648
24	1	0	-4.239623	-1.627604	-0.457482
25	1	0	-1.993013	-2.635850	-0.141080

4

Center Number	Atomic Number	Atomic Type	Coordinates (Angstroms) X	Y	Z
1	6	0	2.714009	-1.733111	0.178623
2	6	0	3.085500	-0.364276	-0.454494
3	6	0	2.040973	0.676391	-0.159197
4	7	0	0.994452	0.260217	0.695068
5	6	0	0.413656	-0.989507	0.318577
6	6	0	1.236379	-2.011763	0.047491
7	7	0	1.932049	1.839603	-0.665793
8	6	0	0.640771	2.408345	-0.232034
9	6	0	-0.014767	1.342042	0.706054
10	6	0	-1.019617	-0.749267	0.138733
11	6	0	-1.278149	0.623687	0.296554
12	6	0	-2.559840	1.125571	0.117909
13	6	0	-3.586758	0.244200	-0.226167
14	6	0	-3.331392	-1.121760	-0.377418
15	6	0	-2.046755	-1.629211	-0.196029
16	1	0	3.309741	-2.523472	-0.280569
17	1	0	2.986399	-1.705655	1.241703
18	1	0	3.165118	-0.450799	-1.540912
19	1	0	4.058258	-0.023673	-0.089410
20	1	0	0.865014	-2.964344	-0.304639
21	1	0	0.805578	3.348640	0.298001
22	1	0	0.031124	2.626491	-1.113909
23	1	0	-0.121452	1.742194	1.718558
24	1	0	-2.764883	2.183284	0.241522
25	1	0	-4.592070	0.621107	-0.373357
26	1	0	-4.141711	-1.792109	-0.638892
27	1	0	-1.850697	-2.688441	-0.314630

5

Center Number	Atomic Number	Atomic Type	Coordinates (Angstroms) X	Y	Z
1	6	0	1.319264	-2.093397	-0.000162
2	1	0	0.976485	-3.119199	-0.009772
3	6	0	0.420001	-1.032843	-0.042763
4	7	0	1.029385	0.192998	-0.020776
5	6	0	0.190222	1.268633	-0.053852
6	6	0	-0.983739	-0.707256	-0.095680
7	6	0	-1.117190	0.692637	-0.099079
8	6	0	-2.471042	1.318731	-0.235196
9	1	0	-2.498539	2.312937	0.216821
10	1	0	-2.682732	1.453419	-1.305531
11	6	0	-3.550320	0.414588	0.390997
12	1	0	-4.541430	0.763046	0.090556
13	1	0	-3.509611	0.510896	1.486200
14	6	0	2.699267	-1.805148	0.058418
15	1	0	3.389371	-2.639808	0.089234
16	6	0	3.234689	-0.506660	0.081325
17	1	0	4.301461	-0.338555	0.128307
18	6	0	2.341211	0.563564	0.041544
19	7	0	2.390582	1.927026	0.046208
20	6	0	1.109279	2.352251	-0.009592
21	1	0	0.881704	3.407933	-0.015831
22	6	0	-2.150614	-1.567807	-0.159681
23	1	0	-2.021955	-2.625529	-0.361615
24	6	0	-3.371663	-1.044981	0.038911
25	1	0	-4.250711	-1.679178	0.012462

6

Center Number	Atomic Number	Atomic Type	Coordinates (Angstroms) X	Y	Z
1	6	0	1.263396	-2.095408	-0.031503
2	1	0	0.895276	-3.112263	-0.055088
3	6	0	0.387408	-1.011415	-0.059530
4	7	0	1.030778	0.200913	-0.024915
5	6	0	0.222825	1.295462	-0.044265
6	6	0	-0.999573	-0.658209	-0.103820
7	6	0	-1.105012	0.744059	-0.098223
8	6	0	2.648687	-1.842480	0.034315
9	1	0	3.317779	-2.694415	0.056333
10	6	0	3.217413	-0.557842	0.077642
11	1	0	4.287922	-0.418403	0.132611
12	6	0	2.352464	0.536183	0.049512
13	7	0	2.436791	1.896373	0.075363
14	6	0	1.165424	2.354739	0.022696
15	1	0	0.964113	3.415635	0.031941
16	6	0	-2.223410	-1.512831	-0.246753
17	1	0	-2.080163	-2.496537	0.207467
18	1	0	-2.419313	-1.684675	-1.314697
19	6	0	-3.439709	-0.811700	0.387646
20	1	0	-3.383473	-0.905955	1.482355
21	1	0	-4.360057	-1.316970	0.084517
22	6	0	-3.511743	0.659641	0.051623
23	1	0	-4.486410	1.135535	0.042755
24	6	0	-2.402876	1.389996	-0.152691
25	1	0	-2.460433	2.455883	-0.341243

7

Center Number	Atomic Number	Atomic Type	Coordinates (Angstroms) X	Y	Z
1	6	0	-1.343277	-2.091345	-0.041480
2	1	0	-0.982776	-3.111057	-0.062388
3	6	0	-0.462617	-1.015923	-0.020851
4	7	0	-1.093351	0.198277	0.004696
5	6	0	-0.271870	1.282148	0.025115
6	6	0	0.938864	-0.673160	-0.014376
7	6	0	1.053989	0.719582	0.011465
8	6	0	2.114746	-1.604430	-0.038237
9	1	0	2.235132	-2.027714	-1.044245
10	1	0	1.944077	-2.453455	0.631808
11	6	0	2.370428	1.434538	0.031740
12	1	0	2.342360	2.299261	-0.638677
13	1	0	2.548700	1.835390	1.038118
14	6	0	3.515747	0.486607	-0.359742
15	1	0	4.477041	0.957151	-0.136660
16	1	0	3.488513	0.316132	-1.442292
17	6	0	3.399871	-0.861612	0.363397
18	1	0	4.271346	-1.485219	0.146669
19	1	0	3.392776	-0.688414	1.445883
20	6	0	-2.731199	-1.826687	-0.036383
21	1	0	-3.407462	-2.673107	-0.053075
22	6	0	-3.288775	-0.539263	-0.011846
23	1	0	-4.359540	-0.390354	-0.009990
24	6	0	-2.413383	0.548925	0.010133
25	7	0	-2.483450	1.908296	0.035394
26	6	0	-1.202830	2.351486	0.044388
27	1	0	-0.990713	3.410180	0.064275

8

Center Number	Atomic Number	Atomic Type	Coordinates (Angstroms) X	Y	Z
1	6	0	2.190960	-0.143137	-0.000000
2	1	0	3.260159	0.020412	-0.000000
3	6	0	1.357507	0.934932	-0.000000
4	1	0	1.694185	1.962332	-0.000000
5	7	0	0.000000	0.732933	0.000000
6	6	0	-1.054243	1.623131	0.000000
7	1	0	-0.902634	2.688841	0.000000
8	6	0	-2.190372	0.841619	0.000000
9	1	0	-3.213021	1.187702	0.000000
10	7	0	-1.894056	-0.489018	0.000000
11	6	0	-0.567007	-0.558065	0.000000
12	6	0	1.660525	-1.463965	-0.000000
13	1	0	2.337922	-2.308530	-0.000000
14	6	0	0.305113	-1.668041	-0.000000
15	1	0	-0.133107	-2.657004	-0.000000

9

Center Number	Atomic Number	Atomic Type	Coordinates (Angstroms) X	Y	Z
1	6	0	-1.987207	0.885130	0.016048
2	1	0	-2.876798	1.499279	-0.041239
3	6	0	-0.790763	1.454235	-0.143832
4	1	0	-0.635970	2.507319	-0.335713
5	7	0	0.373656	0.669981	-0.064024
6	7	0	1.561132	-1.205175	0.033996
7	6	0	0.351834	-0.707579	-0.071081
8	6	0	-2.111620	-0.587099	0.327848
9	1	0	-2.118426	-0.715451	1.419017
10	1	0	-3.067106	-0.974183	-0.033000
11	6	0	-0.954215	-1.401664	-0.281215
12	1	0	-0.903848	-2.405151	0.142172
13	1	0	-1.119142	-1.514138	-1.360809
14	6	0	1.700839	1.054545	0.053352
15	1	0	1.992476	2.090104	0.099293
16	6	0	2.409442	-0.114472	0.115864
17	1	0	3.475451	-0.240008	0.218567

10

Center Number	Atomic Number	Atomic Type	Coordinates (Angstroms) X	Y	Z
1	6	0	-2.284199	0.082640	0.000000
2	1	0	-3.325314	0.370124	0.000000
3	6	0	-1.308621	1.034667	0.000000
4	1	0	-1.516792	2.097370	0.000000
5	7	0	-0.000000	0.676357	0.000000
6	7	0	1.732339	-0.812172	0.000000
7	6	0	0.453449	-0.660210	0.000000
8	6	0	-1.890041	-1.294608	0.000000
9	1	0	-2.659097	-2.059004	0.000000

10	6	0	-0.580675	-1.664393	0.000000
11	1	0	-0.271036	-2.700386	0.000000
12	6	0	1.168756	1.564095	0.000000
13	1	0	1.161483	2.200860	0.888202
14	1	0	1.161483	2.200860	-0.888202
15	6	0	2.336830	0.528082	0.000000
16	1	0	2.974952	0.649622	-0.880086
17	1	0	2.974952	0.649622	0.880086

11

Center Number	Atomic Number	Atomic Type	Coordinates (Angstroms) X	Y	Z
1	6	0	-2.040618	0.900817	0.089230
2	1	0	-2.914383	1.539059	0.116237
3	6	0	-0.842066	1.439360	-0.170116
4	1	0	-0.702769	2.499492	-0.344996
5	7	0	0.315462	0.665591	-0.264845
6	7	0	1.392158	-1.297832	0.110697
7	6	0	0.275483	-0.721468	-0.123668
8	6	0	1.659228	1.112317	0.120859
9	1	0	1.636000	1.571700	1.116629
10	1	0	2.059429	1.837855	-0.590128
11	6	0	2.422823	-0.238931	0.118707
12	1	0	3.042394	-0.347498	-0.777636
13	1	0	3.076127	-0.350161	0.985564
14	6	0	-2.183796	-0.576914	0.359448
15	1	0	-2.168199	-0.757606	1.442606
16	1	0	-3.151682	-0.941601	0.005501
17	6	0	-1.055452	-1.381185	-0.316140
18	1	0	-0.998815	-2.405642	0.050525
19	1	0	-1.245053	-1.423889	-1.395178

12

Center Number	Atomic Number	Atomic Type	Coordinates (Angstroms) X	Y	Z
1	6	0	0.051076	1.422065	0.110802
2	1	0	0.013601	2.506307	0.112398
3	6	0	-0.051076	0.730563	1.255119
4	1	0	-0.188893	1.243185	2.201101
5	6	0	0.051076	-0.730563	1.255119
6	1	0	0.188893	-1.243185	2.201101
7	6	0	-0.051076	-1.422065	0.110802
8	1	0	-0.013601	-2.506307	0.112398
9	6	0	-0.309576	-0.702356	-1.190108
10	1	0	-1.398855	-0.624178	-1.331274
11	1	0	0.068520	-1.280602	-2.037106
12	6	0	0.309576	0.702356	-1.190108
13	1	0	-0.068520	1.280602	-2.037106
14	1	0	1.398855	0.624178	-1.331274

13

Center Number	Atomic Number	Atomic Type	Coordinates (Angstroms) X	Y	Z
1	6	0	-0.120665	-0.656064	1.303401
2	1	0	-0.227666	-1.178502	2.250285
3	6	0	0.120665	0.656064	1.303401
4	1	0	0.227666	1.178502	2.250285
5	6	0	0.253305	1.477288	0.046506
6	1	0	1.305887	1.761185	-0.091600
7	1	0	-0.292381	2.420002	0.164946
8	6	0	-0.253305	0.723007	-1.189702
9	1	0	0.056246	1.241425	-2.101754
10	1	0	-1.349605	0.717297	-1.183107
11	6	0	0.253305	-0.723007	-1.189702
12	1	0	1.349605	-0.717297	-1.183107
13	1	0	-0.056246	-1.241425	-2.101754
14	6	0	-0.253305	-1.477288	0.046506
15	1	0	0.292381	-2.420002	0.164946
16	1	0	-1.305887	-1.761185	-0.091600

14

Center Number	Atomic Number	Atomic Type	Coordinates (Angstroms) X	Y	Z
1	6	0	-0.000000	1.394109	0.000000
2	1	0	-0.000000	2.478130	0.000000
3	6	0	1.207334	0.697054	0.000000
4	1	0	2.146124	1.239065	0.000000
5	6	0	1.207334	-0.697054	0.000000
6	1	0	2.146124	-1.239065	0.000000
7	6	0	-0.000000	-1.394109	0.000000
8	1	0	-0.000000	-2.478130	0.000000
9	6	0	-1.207334	0.697054	0.000000
10	1	0	-2.146124	1.239065	0.000000
11	6	0	-1.207334	-0.697054	0.000000
12	1	0	-2.146124	-1.239065	0.000000

15a

Center Number	Atomic Number	Atomic Type	Coordinates (Angstroms) X	Y	Z
1	8	0	-1.577182	0.000092	-0.700894
2	8	0	-0.000000	-2.150065	0.024390
3	8	0	1.577182	-0.000092	-0.700894
4	8	0	0.000000	2.150065	0.024390
5	6	0	1.251230	-2.394339	-0.660886
6	6	0	1.703784	-1.204248	-1.491455
7	6	0	1.699920	1.202329	-1.494705
8	6	0	1.248635	2.394074	-0.665808
9	6	0	-1.251230	2.394339	-0.660886
10	6	0	-1.703784	1.204248	-1.491455
11	6	0	-1.699920	-1.202329	-1.494705
12	6	0	-1.248635	-2.394074	-0.665808
13	1	0	1.169101	-3.284150	-1.289263
14	1	0	1.971306	-2.614703	0.130057
15	1	0	2.745678	-1.348334	-1.792740
16	1	0	1.098920	-1.091593	-2.394837

17	1	0	2.740743	1.346950	-1.799443
18	1	0	1.092525	1.086585	-2.395996
19	1	0	1.163034	3.281921	-1.296495
20	1	0	1.971130	2.617558	0.121979
21	1	0	-1.169101	3.284150	-1.289263
22	1	0	-1.971306	2.614703	0.130057
23	1	0	-2.745678	1.348334	-1.792740
24	1	0	-1.098920	1.091593	-2.394837
25	1	0	-2.740743	-1.346950	-1.799443
26	1	0	-1.092525	-1.086585	-2.395996
27	1	0	-1.163034	-3.281921	-1.296495
28	1	0	-1.971130	-2.617558	0.121979
29	56	0	-0.000000	0.000000	1.500210

15b

Center Number	Atomic Number	Atomic Type	Coordinates (Angstroms) X	Y	Z
1	8	0	-1.035764	-2.029192	0.308120
2	8	0	-2.416115	0.384164	-0.096724
3	8	0	-0.379781	2.069781	0.471399
4	8	0	2.139603	1.106290	0.176220
5	8	0	1.594125	-1.516150	0.284950
6	6	0	-2.611364	1.390389	0.923705
7	6	0	-1.727875	2.573133	0.590713
8	6	0	0.697561	3.030804	0.436023
9	6	0	1.946680	2.328772	0.925082
10	6	0	3.116788	0.234814	0.792951
11	6	0	2.989399	-1.158169	0.208087
12	6	0	1.210635	-2.898474	0.431588
13	6	0	-0.164872	-2.900213	1.067144
14	6	0	-2.323810	-1.838867	0.935878
15	6	0	-3.128607	-0.859593	0.103551
16	1	0	-3.659589	1.700511	0.936203
17	1	0	-2.341772	0.980319	1.901168
18	1	0	-1.782957	3.308705	1.397429
19	1	0	-2.032849	3.051079	-0.346299
20	1	0	0.478714	3.871418	1.099229
21	1	0	0.812975	3.420690	-0.582169
22	1	0	2.814661	2.983152	0.803762
23	1	0	1.834917	2.072992	1.982630
24	1	0	4.124610	0.624854	0.625074
25	1	0	2.918759	0.200706	1.867953
26	1	0	3.596691	-1.852151	0.793984
27	1	0	3.327499	-1.203678	-0.833476
28	1	0	1.912424	-3.421561	1.085812
29	1	0	1.211618	-3.392250	-0.546197
30	1	0	-0.571710	-3.915319	1.076772
31	1	0	-0.105328	-2.525788	2.093578
32	1	0	-2.857235	-2.792948	0.988291
33	1	0	-2.167774	-1.470906	1.955401
34	1	0	-4.096017	-0.669927	0.574346
35	1	0	-3.316776	-1.261603	-0.894137
36	56	0	0.011703	0.013517	-1.136061

15c

Center Number	Atomic Number	Atomic Type	Coordinates (Angstroms) X	Y	Z
1	56	0	0.000123	-0.000423	-0.002400
2	6	0	2.204945	2.910899	-0.322293
3	8	0	0.846774	2.623554	0.067138
4	8	0	2.695544	0.578392	-0.066210
5	6	0	3.116775	1.900601	0.326369
6	8	0	1.849036	-2.044770	0.068396
7	6	0	3.204936	-1.749308	-0.322627
8	6	0	3.622553	-0.453815	0.325708
9	8	0	-0.847097	-2.623562	-0.067685
10	6	0	0.087166	-3.649748	0.323456
11	6	0	1.418181	-3.363604	-0.324077
12	6	0	-0.087332	3.649624	-0.324806
13	6	0	-1.418176	3.364432	0.323558
14	8	0	-1.849274	2.044942	-0.066955
15	6	0	-3.204582	1.749118	0.326334
16	6	0	-3.623328	0.454123	-0.321843
17	8	0	-2.694920	-0.578185	0.067581
18	6	0	-3.117629	-1.900373	-0.322205
19	6	0	-2.204409	-2.909486	0.325909
20	1	0	2.287348	2.872319	-1.413985
21	1	0	2.482932	3.913407	0.016377
22	1	0	3.083782	1.984363	1.418147
23	1	0	4.143036	2.077180	-0.009183
24	1	0	3.262895	-1.680116	-1.414396
25	1	0	3.870404	-2.549357	0.015084
26	1	0	4.630487	-0.193651	-0.011038
27	1	0	3.628195	-0.543554	1.417498
28	1	0	0.175532	-3.665486	1.415242
29	1	0	-0.272638	-4.626150	-0.014465
30	1	0	2.147129	-4.106674	0.012753
31	1	0	1.343631	-3.412944	-1.415908
32	1	0	-0.176014	3.664287	-1.416599
33	1	0	0.272784	4.626297	0.011980
34	1	0	-2.147246	4.106978	-0.014186
35	1	0	-1.343316	3.415292	1.415279
36	1	0	-3.260484	1.679192	1.418162
37	1	0	-3.870634	2.549468	-0.009528
38	1	0	-4.630393	0.193476	0.017078
39	1	0	-3.631362	0.544382	-1.413565
40	1	0	-3.087610	-1.985882	-1.413928
41	1	0	-4.143018	-2.076145	0.016408
42	1	0	-2.483714	-3.912696	-0.009534
43	1	0	-2.283712	-2.868016	1.417696

15d

Center Number	Atomic Number	Atomic Type	Coordinates (Angstroms) X	Y	Z
1	8	0	0.688775	-2.474166	-0.736065
2	8	0	2.370419	-1.275457	1.107103
3	8	0	2.589161	1.309724	0.072627
4	8	0	0.360413	2.654847	-0.842766
5	8	0	-2.013046	1.893595	0.387504
6	8	0	-2.258792	-0.670904	1.440460
7	8	0	-1.790124	-1.321739	-1.258996
8	6	0	1.596309	-3.277431	0.042473
9	6	0	2.803774	-2.443226	0.386350
10	6	0	-1.197727	-2.266241	-2.170885

11	6	0	-0.300918	-3.242389	-1.440707
12	6	0	-3.316449	1.448260	0.795363
13	6	0	-3.128748	0.395829	1.870357
14	6	0	3.444309	-0.394524	1.493530
15	6	0	3.787543	0.578046	0.383937
16	6	0	-0.718223	3.597733	-0.684663
17	6	0	-2.035528	2.865089	-0.673869
18	6	0	2.740140	2.291461	-0.968350
19	6	0	1.641830	3.314315	-0.820448
20	6	0	-2.874352	-1.834905	0.860457
21	6	0	-3.038693	-1.711140	-0.647006
22	1	0	1.920058	-4.145934	-0.538858
23	1	0	1.087468	-3.629730	0.946520
24	1	0	3.483805	-3.029918	1.011641
25	1	0	3.328530	-2.147198	-0.527794
26	1	0	-1.970920	-2.796994	-2.731029
27	1	0	-0.614751	-1.676739	-2.881995
28	1	0	0.186405	-3.908755	-2.160228
29	1	0	-0.867274	-3.855306	-0.729697
30	1	0	-3.884600	2.288892	1.207996
31	1	0	-3.858515	1.059039	-0.072225
32	1	0	-4.096184	-0.005894	2.181726
33	1	0	-2.647579	0.837250	2.745052
34	1	0	4.325413	-0.973539	1.782322
35	1	0	3.091695	0.145858	2.374032
36	1	0	4.566219	1.266844	0.727153
37	1	0	4.150344	0.064657	-0.513403
38	1	0	-0.707529	4.312639	-1.513580
39	1	0	-0.579925	4.142697	0.255130
40	1	0	-2.836103	3.590971	-0.502998
41	1	0	-2.223540	2.358374	-1.627732
42	1	0	3.704887	2.798249	-0.872983
43	1	0	2.703747	1.797727	-1.946292
44	1	0	1.701392	4.034002	-1.643143
45	1	0	1.748782	3.850875	0.128258
46	1	0	-3.842708	-2.024858	1.332322
47	1	0	-2.213199	-2.668450	1.106240
48	1	0	-3.378725	-2.668072	-1.051610
49	1	0	-3.779509	-0.955678	-0.912467
50	56	0	0.098362	0.090831	0.223383

16a

Center Number	Atomic Number	Atomic Type	Coordinates (Angstroms) X	Y	Z
1	8	0	-0.055502	2.893191	0.096623
2	8	0	-1.533061	0.262840	-0.311448
3	8	0	0.055502	-2.893191	0.096623
4	8	0	1.533061	-0.262840	-0.311448
5	6	0	-1.434968	-0.967307	0.387210
6	6	0	-0.862130	-2.023379	-0.567337
7	6	0	1.434968	-2.646554	-0.136722
8	6	0	1.968830	-1.360393	0.475089
9	6	0	1.434968	0.967307	0.387210
10	6	0	0.862130	2.023379	-0.567337
11	6	0	-1.434968	2.646554	-0.136722
12	6	0	-1.968830	1.360393	0.475089
13	1	0	-2.418870	-1.267795	0.773858
14	1	0	-0.762253	-0.861657	1.247055
15	1	0	-1.657731	-2.657661	-0.965542
16	1	0	-0.376941	-1.500954	-1.394028
17	1	0	1.957914	-3.495785	0.309517
18	1	0	1.644685	-2.639726	-1.215000
19	1	0	3.068340	-1.395370	0.507118

20	1	0	1.602850	-1.266691	1.506176
21	1	0	2.418870	1.267795	0.773858
22	1	0	0.762253	0.861657	1.247055
23	1	0	1.657731	2.657661	-0.965542
24	1	0	0.376941	1.500954	-1.394028
25	1	0	-1.957914	3.495785	0.309517
26	1	0	-1.644685	2.639726	-1.215000
27	1	0	-3.068340	1.395370	0.507118
28	1	0	-1.602850	1.266691	1.506176

16b

Center Number	Atomic Number	Atomic Type	Coordinates (Angstroms) X	Y	Z
1	8	0	3.230866	-0.764654	0.391304
2	8	0	0.150509	-1.213400	-0.140602
3	8	0	-3.086856	-1.245829	-0.178425
4	8	0	-2.000489	2.198378	0.442816
5	8	0	1.501583	2.298676	-0.598505
6	6	0	-0.855897	-1.992169	0.539592
7	6	0	-2.033847	-2.193058	-0.419125
8	6	0	-2.791548	0.125599	-0.524353
9	6	0	-2.486128	0.885026	0.774381
10	6	0	-0.578731	2.394453	0.539786
11	6	0	0.128440	1.859938	-0.710500
12	6	0	2.499223	1.267599	-0.660488
13	6	0	2.582629	0.499668	0.664976
14	6	0	2.389095	-1.925568	0.472858
15	6	0	1.307435	-1.931257	-0.611479
16	1	0	-0.450772	-2.938811	0.923525
17	1	0	-1.146323	-1.338753	1.391080
18	1	0	-2.561813	-3.149853	-0.242267
19	1	0	-1.705791	-2.131434	-1.472342
20	1	0	-3.729183	0.473165	-0.995604
21	1	0	-1.966010	0.198929	-1.249850
22	1	0	-3.407005	1.097398	1.349309
23	1	0	-1.778511	0.326441	1.412499
24	1	0	-0.505994	3.498886	0.602461
25	1	0	-0.179600	1.950515	1.465029
26	1	0	-0.250652	2.339891	-1.630251
27	1	0	0.042747	0.761618	-0.801502
28	1	0	3.414311	1.857193	-0.861417
29	1	0	2.311780	0.586392	-1.508501
30	1	0	3.257880	0.984978	1.391154
31	1	0	1.585934	0.361219	1.117048
32	1	0	3.128345	-2.737488	0.327979
33	1	0	1.949763	-1.992977	1.483411
34	1	0	1.041287	-2.946937	-0.940023
35	1	0	1.620682	-1.327136	-1.488340

16c

Center Number	Atomic Number	Atomic Type	Coordinates (Angstroms) X	Y	Z
1	6	0	1.381351	-1.927326	0.155572
2	8	0	0.137799	-1.904565	-0.535633
3	8	0	3.736658	-1.454777	-0.252961
4	6	0	2.461351	-1.608218	-0.869786
5	8	0	2.509214	0.920697	1.080786
6	6	0	3.903139	0.690430	0.953308

7	6	0	4.287600	-0.147306	-0.263067
8	8	0	-0.167855	2.643570	-0.602329
9	6	0	0.465702	1.621201	0.163004
10	6	0	1.955723	1.906773	0.218857
11	6	0	-0.987686	-1.967192	0.326301
12	6	0	-2.232431	-1.955131	-0.549928
13	8	0	-3.411754	-1.918088	0.253257
14	6	0	-4.151568	-0.709151	0.238159
15	6	0	-3.529545	0.414265	1.059903
16	8	0	-2.348777	0.941308	0.464090
17	6	0	-2.559042	2.031146	-0.422688
18	6	0	-1.329351	2.225328	-1.294588
19	1	0	1.398933	-1.172328	0.947116
20	1	0	1.553983	-2.914497	0.605199
21	1	0	2.176384	-0.698623	-1.408357
22	1	0	2.534893	-2.420002	-1.599957
23	1	0	4.194214	0.154295	1.859120
24	1	0	4.455880	1.642117	0.923235
25	1	0	5.375915	-0.267090	-0.256436
26	1	0	4.020741	0.366382	-1.195520
27	1	0	0.054488	1.583609	1.175291
28	1	0	0.298999	0.645137	-0.301434
29	1	0	2.380105	1.848329	-0.790324
30	1	0	2.148506	2.914088	0.611008
31	1	0	-0.998723	-1.102979	0.999853
32	1	0	-0.963193	-2.883055	0.932652
33	1	0	-2.269473	-2.866387	-1.153775
34	1	0	-2.188174	-1.095051	-1.223185
35	1	0	-4.315465	-0.375250	-0.796603
36	1	0	-5.126893	-0.956734	0.667009
37	1	0	-4.269814	1.211349	1.216754
38	1	0	-3.239123	0.023274	2.038095
39	1	0	-2.767860	2.944778	0.151035
40	1	0	-3.415550	1.838267	-1.085635
41	1	0	-1.545942	3.012528	-2.021782
42	1	0	-1.126561	1.293766	-1.841309

16d

Center Number	Atomic Number	Atomic Type	Coordinates (Angstroms) X	Y	Z
1	8	0	2.589853	2.558905	0.086766
2	8	0	3.346817	-0.137810	-1.027724
3	8	0	2.878462	-1.751762	1.368902
4	8	0	-0.555131	-2.268125	0.291659
5	8	0	-3.928943	-1.854001	-1.012800
6	8	0	-3.597425	0.820928	0.345932
7	8	0	-0.983415	2.161859	0.103650
8	6	0	3.703019	2.217559	-0.721709
9	6	0	4.233164	0.818481	-0.463243
10	6	0	0.294401	2.477092	0.630367
11	6	0	1.326310	2.161721	-0.435281
12	6	0	-4.903715	-1.081196	-0.333983
13	6	0	-4.698027	0.417200	-0.449133
14	6	0	3.778303	-1.483051	-0.931936
15	6	0	3.962466	-2.001649	0.496501
16	6	0	-1.579579	-1.558713	-0.397107
17	6	0	-2.853771	-2.376257	-0.239187
18	6	0	1.668897	-2.445207	1.079322
19	6	0	0.656660	-1.544412	0.382747
20	6	0	-3.361608	2.216714	0.298749
21	6	0	-2.043255	2.534464	0.964270
22	1	0	4.491207	2.932601	-0.469133
23	1	0	3.460883	2.334139	-1.786469

24	1	0	5.231414	0.720803	-0.920150
25	1	0	4.326743	0.671682	0.618592
26	1	0	0.351257	3.541838	0.897348
27	1	0	0.499039	1.889864	1.536256
28	1	0	1.098506	2.725301	-1.350531
29	1	0	1.316612	1.096957	-0.672426
30	1	0	-5.868142	-1.322828	-0.793106
31	1	0	-4.950691	-1.362781	0.725432
32	1	0	-5.616548	0.924664	-0.111823
33	1	0	-4.525307	0.685644	-1.500706
34	1	0	4.732172	-1.622271	-1.466448
35	1	0	3.018852	-2.066067	-1.456714
36	1	0	4.175090	-3.080243	0.433694
37	1	0	4.824941	-1.528087	0.969396
38	1	0	-1.329063	-1.464034	-1.463210
39	1	0	-1.707157	-0.554525	0.015343
40	1	0	-2.669455	-3.394556	-0.592373
41	1	0	-3.129066	-2.429126	0.820158
42	1	0	1.862665	-3.338494	0.473094
43	1	0	1.247593	-2.774115	2.032574
44	1	0	1.008612	-1.248629	-0.612788
45	1	0	0.520624	-0.627794	0.971766
46	1	0	-4.171315	2.752733	0.817148
47	1	0	-3.330566	2.566098	-0.742317
48	1	0	-1.998031	3.614395	1.171486
49	1	0	-1.972209	2.000400	1.922556

17a

Center Number	Atomic Number	Atomic Type	Coordinates (Angstroms) X	Y	Z
1	1	0	3.984316	0.381024	-2.448522
2	6	0	3.764462	0.214134	-1.400035
3	6	0	3.318308	-0.217196	1.332061
4	6	0	3.689254	-1.093072	-0.900208
5	6	0	3.616801	1.305345	-0.533517
6	6	0	3.393684	1.089663	0.832921
7	6	0	3.466563	-1.308664	0.466096
8	1	0	3.851167	-1.935972	-1.562607
9	1	0	3.722678	2.315612	-0.912451
10	1	0	3.324148	1.933684	1.509610
11	1	0	3.454388	-2.318383	0.860426
12	1	0	3.195191	-0.384856	2.396012
13	56	0	0.439844	-0.014427	-0.522099
14	8	0	-0.752049	-1.130605	1.628327
15	8	0	-0.891512	1.606152	1.184695
16	8	0	-1.727159	-1.598036	-0.924721
17	8	0	-1.866398	1.137301	-1.370641
18	6	0	-1.910769	-1.978921	1.478181
19	1	0	-2.821036	-1.384317	1.598379
20	1	0	-1.899647	-2.756854	2.246980
21	6	0	-1.844217	-2.609619	0.100210
22	1	0	-2.719532	-3.238388	-0.078844
23	1	0	-0.957588	-3.240144	0.006397
24	6	0	-0.802586	-0.208116	2.738650
25	1	0	-1.271093	-0.677669	3.606773
26	1	0	0.236233	0.009642	2.994682
27	6	0	-1.536378	1.063302	2.356296
28	1	0	-2.589414	0.862943	2.138092
29	1	0	-1.483090	1.786519	3.175172
30	6	0	-1.639885	2.626336	0.486110
31	1	0	-0.901618	3.238060	-0.036639
32	1	0	-2.163545	3.271005	1.195832
33	6	0	-2.615870	2.007208	-0.496123

34	1	0	-3.387959	1.431509	0.022739
35	1	0	-3.102694	2.791063	-1.083487
36	6	0	-2.977738	-1.035922	-1.377234
37	1	0	-3.494290	-1.754970	-2.019481
38	1	0	-3.615951	-0.818211	-0.515868
39	6	0	-2.662461	0.224877	-2.159443
40	1	0	-2.067968	-0.007882	-3.045417
41	1	0	-3.582039	0.709855	-2.495283

17b

Center Number	Atomic Number	Atomic Type	Coordinates (Angstroms) X	Y	Z
1	1	0	3.771473	-0.386524	-1.582428
2	6	0	3.581518	-0.152503	-0.541013
3	6	0	3.215107	0.455894	2.167391
4	6	0	3.499605	-1.183737	0.402417
5	6	0	3.479736	1.182568	-0.131529
6	6	0	3.296159	1.486979	1.222807
7	6	0	3.316139	-0.879655	1.756891
8	1	0	3.617955	-2.215273	0.091517
9	1	0	3.584583	1.982515	-0.855405
10	1	0	3.262165	2.521139	1.546040
11	1	0	3.296943	-1.675299	2.492934
12	1	0	3.118599	0.692866	3.220918
13	56	0	0.229373	0.030468	0.450791
14	8	0	-2.123384	-0.974937	1.284984
15	8	0	-1.745790	1.744325	1.175049
16	8	0	-0.464455	-2.502586	-0.309284
17	8	0	-0.540220	1.997294	-1.332535
18	6	0	-2.575924	-2.294904	0.918717
19	1	0	-3.234082	-2.219207	0.047365
20	1	0	-3.135516	-2.745531	1.743605
21	6	0	-1.346441	-3.140255	0.637728
22	1	0	-1.639006	-4.130963	0.280551
23	1	0	-0.761139	-3.271134	1.550061
24	6	0	-3.113005	-0.091925	1.865428
25	1	0	-4.108749	-0.532957	1.785008
26	1	0	-2.871848	0.038603	2.922724
27	6	0	-3.092678	1.232464	1.133226
28	1	0	-3.409167	1.099145	0.093490
29	1	0	-3.770505	1.936755	1.625258
30	6	0	-1.553200	3.042927	0.579208
31	1	0	-0.602790	3.407343	0.974864
32	1	0	-2.336780	3.731417	0.907166
33	6	0	-1.515923	2.980317	-0.936457
34	1	0	-2.489630	2.700525	-1.351838
35	1	0	-1.245088	3.964023	-1.333308
36	6	0	-0.570127	1.720383	-2.746918
37	1	0	-1.603628	1.515664	-3.043643
38	1	0	-0.202464	2.585050	-3.307763
39	6	0	0.295288	0.511721	-3.029982
40	1	0	0.167689	0.215135	-4.074518
41	1	0	1.357091	0.721208	-2.856316
42	8	0	-0.144149	-0.539042	-2.150827
43	6	0	-0.748036	-2.785041	-1.694473
44	1	0	-1.801471	-2.580085	-1.907031
45	1	0	-0.534892	-3.836624	-1.906963
46	6	0	0.130617	-1.895695	-2.545057
47	1	0	1.192343	-2.121250	-2.395508
48	1	0	-0.116005	-2.040398	-3.600478

17c

Center Number	Atomic Number	Atomic Type	Coordinates (Angstroms) X	Y	Z
1	1	0	-0.835923	2.522496	-2.947190
2	6	0	-0.347601	1.555166	-2.969537
3	6	0	0.920632	-0.934832	-3.124770
4	6	0	-1.106572	0.393714	-3.153342
5	6	0	1.045282	1.472579	-2.864711
6	6	0	1.678924	0.227132	-2.943375
7	6	0	-0.472865	-0.851514	-3.229364
8	1	0	-2.181911	0.463320	-3.264781
9	1	0	1.634677	2.375396	-2.756412
10	1	0	2.760563	0.167459	-2.907566
11	1	0	-1.055921	-1.747849	-3.405886
12	1	0	1.413292	-1.895221	-3.222252
13	56	0	-0.011414	-0.021220	0.127057
14	8	0	-1.584447	-2.273051	0.645914
15	8	0	2.671770	-0.255567	0.818727
16	8	0	-2.793421	0.162735	0.005585
17	8	0	1.452155	2.201779	0.986196
18	6	0	-3.000707	-2.119641	0.844221
19	1	0	-3.199748	-1.815057	1.878332
20	1	0	-3.507759	-3.072436	0.660458
21	6	0	-3.484579	-1.089323	-0.153025
22	1	0	-4.563869	-0.943818	-0.059533
23	1	0	-3.269391	-1.425988	-1.168764
24	6	0	-0.990953	-3.324249	1.427860
25	1	0	-1.629140	-4.213076	1.408046
26	6	0	3.456279	0.933276	0.637007
27	1	0	3.504447	1.188736	-0.427859
28	1	0	4.474074	0.772755	1.005312
29	6	0	2.809677	2.037437	1.435030
30	1	0	2.811800	1.789124	2.501791
31	1	0	3.364046	2.969650	1.286781
32	6	0	0.764905	3.248566	1.693933
33	1	0	0.650985	2.962655	2.745404
34	1	0	1.345797	4.175139	1.643002
35	6	0	-0.579253	3.476316	1.052263
36	1	0	-1.114337	4.247597	1.614862
37	1	0	-0.468997	3.812359	0.014903
38	8	0	-1.324563	2.245689	1.079511
39	6	0	-3.420404	1.102553	0.895170
40	1	0	-3.362503	0.746099	1.929624
41	1	0	-4.473136	1.226094	0.623825
42	6	0	-2.713044	2.425496	0.747391
43	1	0	-2.798582	2.794891	-0.280472
44	1	0	-3.163663	3.156615	1.425805
45	1	0	-0.887132	-2.991859	2.467223
46	6	0	0.352139	-3.667587	0.827297
47	1	0	0.237304	-4.121826	-0.160821
48	1	0	0.860634	-4.377203	1.485149
49	8	0	1.134955	-2.463953	0.691340
50	6	0	3.287943	-1.464335	0.350199
51	1	0	4.334415	-1.504491	0.667502
52	1	0	3.253886	-1.493469	-0.745280
53	6	0	2.542120	-2.633522	0.950004
54	1	0	2.699938	-2.685522	2.031340
55	1	0	2.902208	-3.558728	0.490578

17d

Center Number	Atomic Number	Atomic Type	Coordinates (Angstroms) X	Y	Z
1	1	0	-1.039696	-2.981625	2.580397
2	6	0	-0.071213	-2.501277	2.528040
3	6	0	2.462243	-1.321043	2.455111
4	6	0	0.191776	-1.365398	3.298156
5	6	0	0.928342	-3.040738	1.712249
6	6	0	2.194516	-2.450874	1.676492
7	6	0	1.461219	-0.774004	3.261898
8	1	0	-0.568375	-0.968932	3.961755
9	1	0	0.733537	-3.942628	1.143364
10	1	0	2.982091	-2.891886	1.077036
11	1	0	1.677734	0.078882	3.895144
12	1	0	3.455707	-0.891165	2.453345
13	56	0	0.182893	0.073831	0.033374
14	8	0	1.243690	2.647654	0.387638
15	8	0	1.670291	-0.800888	-2.173709
16	8	0	-1.504823	2.329059	-0.072434
17	8	0	-1.065720	-1.252621	-2.071620
18	6	0	0.423363	3.791783	0.094440
19	1	0	0.444569	3.988680	-0.983411
20	1	0	0.811612	4.670099	0.620584
21	6	0	-0.979253	3.512097	0.568962
22	1	0	-1.606176	4.375667	0.337836
23	1	0	-0.998034	3.357532	1.652306
24	6	0	2.646449	2.904522	0.201054
25	1	0	2.957973	3.743115	0.832489
26	6	0	1.091235	-1.731364	-3.105118
27	1	0	1.172438	-2.750182	-2.709086
28	1	0	1.627129	-1.687774	-4.058679
29	6	0	-0.347464	-1.314896	-3.315894
30	1	0	-0.387794	-0.307467	-3.733512
31	1	0	-0.847721	-1.995928	-4.009456
32	6	0	-3.636134	0.405391	1.317832
33	1	0	-2.800463	1.090239	1.495003
34	8	0	-4.269046	0.673521	0.074661
35	6	0	-2.817665	2.540232	-0.669682
36	1	0	-2.681746	3.119697	-1.587831
37	1	0	-3.437871	3.115976	0.022206
38	6	0	-3.495434	1.220715	-0.983398
39	1	0	-4.218128	1.396994	-1.781868
40	1	0	-2.758798	0.503340	-1.367184
41	1	0	2.831972	3.173017	-0.844994
42	6	0	3.399388	1.657830	0.607482
43	1	0	3.201513	1.428292	1.655789
44	1	0	4.476225	1.804572	0.486053
45	8	0	2.959843	0.512677	-0.139594
46	6	0	3.095480	-0.935409	-2.025742
47	1	0	3.561262	-1.071757	-3.006069
48	1	0	3.317898	-1.809116	-1.404253
49	6	0	3.625473	0.328331	-1.398036
50	1	0	3.439472	1.183499	-2.056340
51	1	0	4.704086	0.232808	-1.237361
52	1	0	-4.379789	0.591165	2.097702
53	6	0	-1.628508	-2.497742	-1.613958
54	1	0	-0.851197	-3.110681	-1.142527
55	1	0	-2.042675	-3.053063	-2.460044
56	6	0	-2.735477	-2.182690	-0.639523
57	1	0	-3.549987	-1.649376	-1.135723
58	1	0	-3.126640	-3.118673	-0.228151
59	8	0	-2.201433	-1.370174	0.420439
60	6	0	-3.182966	-1.037516	1.432769
61	1	0	-4.036539	-1.710858	1.336251
62	1	0	-2.720237	-1.208545	2.408425

19 (S_0) BHandH

Center Number	Atomic Number	Atomic Type	Coordinates (Angstroms) X	Y	Z
1	6	0	-1.670492	-0.250365	-0.305750
2	6	0	-0.967460	0.798483	0.262092
3	6	0	-0.938701	-1.234219	-0.951972
4	6	0	0.397710	0.880428	0.178413
5	1	0	-1.499364	1.567854	0.805680
6	6	0	0.425578	-1.168035	-1.045969
7	1	0	-1.466008	-2.074540	-1.382614
8	6	0	1.140417	-0.100792	-0.487747
9	1	0	0.891909	1.731134	0.622153
10	1	0	0.947509	-1.974397	-1.538409
11	6	0	7.333370	-1.295987	2.672102
12	1	0	7.841939	-2.076783	2.099259
13	1	0	7.565646	-1.470785	3.727748
14	6	0	7.842167	0.052954	2.270423
15	1	0	8.903172	0.140632	2.531592
16	1	0	7.296221	0.818187	2.822948
17	6	0	8.595972	-0.226863	0.069846
18	1	0	8.812976	-1.272973	0.318291
19	1	0	9.535546	0.332859	0.154645
20	6	0	8.086345	-0.175843	-1.332767
21	1	0	7.118433	-0.685769	-1.375017
22	1	0	8.787026	-0.708824	-1.980849
23	6	0	6.680161	1.515365	-2.172330
24	1	0	6.234272	0.757701	-2.828224
25	1	0	6.772687	2.436993	-2.746277
26	6	0	5.794918	1.723234	-0.981864
27	1	0	6.184047	2.539672	-0.361120
28	1	0	5.803237	0.816381	-0.374292
29	6	0	3.597603	2.174548	-0.391881
30	1	0	2.704914	2.630677	-0.820776
31	1	0	4.002233	2.858966	0.364638
32	6	0	3.243509	0.865323	0.262743
33	1	0	2.685260	1.062647	1.176897
34	1	0	4.150181	0.342257	0.571546
35	6	0	3.232136	-0.840056	-1.493741
36	1	0	3.994244	-0.218302	-1.968257
37	1	0	2.571127	-1.175126	-2.289029
38	6	0	3.920443	-2.019976	-0.871502
39	1	0	4.129476	-2.777704	-1.636077
40	1	0	3.275593	-2.476183	-0.110986
41	6	0	5.805307	-2.564090	0.397760
42	1	0	5.567455	-3.558022	0.003604
43	1	0	6.871913	-2.399060	0.233480
44	6	0	5.498738	-2.520897	1.864501
45	1	0	4.418887	-2.550343	2.015964
46	1	0	5.926475	-3.404513	2.352158
47	7	0	2.491832	-0.022155	-0.577768
48	8	0	4.513556	2.028969	-1.433286
49	8	0	7.963110	1.140547	-1.786062
50	8	0	7.638536	0.308308	0.919714
51	8	0	5.955875	-1.353488	2.469409
52	8	0	5.120791	-1.581849	-0.311633
53	6	0	-5.050258	-1.258233	-0.268138
54	6	0	-4.024017	0.682042	0.087879
55	7	0	-5.199792	0.041101	0.049913
56	6	0	-6.306112	0.772734	0.275747
57	6	0	-7.479499	0.080512	0.207806
58	6	0	-7.413802	-1.288450	-0.096343

59	6	0	-6.234154	-1.969362	-0.339611
60	1	0	-8.435393	0.551772	0.372058
61	1	0	-6.260740	-3.021042	-0.580994
62	6	0	-4.374306	2.045337	0.369714
63	6	0	-5.788367	2.096603	0.496703
64	6	0	-3.640517	3.212976	0.506301
65	6	0	-6.435480	3.286148	0.771168
66	6	0	-5.685508	4.425750	0.912080
67	6	0	-4.302409	4.384870	0.774878
68	1	0	-3.734412	5.298997	0.880272
69	1	0	-7.512304	3.316091	0.868428
70	1	0	-2.566458	3.216145	0.392201
71	7	0	-3.751675	-1.499086	-0.432624
72	6	0	-3.106292	-0.339140	-0.217008
73	1	0	-6.171975	5.366897	1.126013
74	6	0	-8.652300	-2.077934	-0.180196
75	8	0	-8.685961	-3.245134	-0.442209
76	8	0	-9.726494	-1.368480	0.063849
77	6	0	-10.952695	-2.058268	0.001911
78	1	0	-11.720356	-1.329141	0.233937
79	1	0	-11.107217	-2.466567	-0.994351
80	1	0	-10.968022	-2.868578	0.727331

19 (S_1) BHandH

Center Number	Atomic Number	Atomic Type	Coordinates (Angstroms) X	Y	Z
1	6	0	-1.670601	-0.277856	-0.340993
2	6	0	-0.937972	0.762678	0.250155
3	6	0	-0.943248	-1.257489	-1.037027
4	6	0	0.439523	0.840017	0.143243
5	1	0	-1.451792	1.520082	0.827902
6	6	0	0.433292	-1.194834	-1.154329
7	1	0	-1.477029	-2.083558	-1.489820
8	6	0	1.176377	-0.135975	-0.572649
9	1	0	0.942780	1.675535	0.607648
10	1	0	0.937314	-1.988619	-1.686491
11	6	0	7.227887	-1.300849	2.702754
12	1	0	7.721928	-2.069992	2.100645
13	1	0	7.357031	-1.578490	3.757965
14	6	0	7.875639	0.051127	2.458460
15	1	0	8.909963	0.028975	2.826035
16	1	0	7.334059	0.819826	3.014030
17	6	0	8.793775	-0.162827	0.246055
18	1	0	8.993936	-1.195776	0.555515
19	1	0	9.739987	0.392025	0.297126
20	6	0	8.275218	-0.200494	-1.177424
21	1	0	7.304038	-0.703828	-1.195298
22	1	0	8.977780	-0.779998	-1.787260
23	6	0	6.866120	1.466022	-2.197967
24	1	0	6.410589	0.637572	-2.754252
25	1	0	6.995396	2.304474	-2.885121
26	6	0	5.965776	1.854537	-1.033628
27	1	0	6.331217	2.774382	-0.557922
28	1	0	5.992188	1.061285	-0.285050
29	6	0	3.678069	2.170219	-0.482793
30	1	0	2.788846	2.609185	-0.938583
31	1	0	4.047032	2.858890	0.289570
32	6	0	3.335068	0.824689	0.159894
33	1	0	2.799358	1.000571	1.093530

34	1	0	4.247953	0.293366	0.430989
35	6	0	3.273993	-0.912440	-1.625646
36	1	0	4.087908	-0.324547	-2.052337
37	1	0	2.617092	-1.179318	-2.452377
38	6	0	3.862865	-2.174272	-1.005143
39	1	0	4.032879	-2.933250	-1.780820
40	1	0	3.169388	-2.589218	-0.264567
41	6	0	5.609808	-2.799889	0.518173
42	1	0	5.222865	-3.798008	0.281000
43	1	0	6.693366	-2.830840	0.381214
44	6	0	5.260105	-2.450928	1.959254
45	1	0	4.180094	-2.320861	2.057193
46	1	0	5.564437	-3.273822	2.620168
47	7	0	2.544214	-0.060686	-0.691740
48	8	0	4.643620	2.055227	-1.529427
49	8	0	8.169879	1.110604	-1.745822
50	8	0	7.828351	0.449989	1.093594
51	8	0	5.841749	-1.221082	2.384315
52	8	0	5.107398	-1.829591	-0.396154
53	6	0	-5.108171	-1.271110	-0.254755
54	6	0	-4.034806	0.691823	0.090325
55	7	0	-5.236493	0.053212	0.069342
56	6	0	-6.351043	0.811353	0.295644
57	6	0	-7.544926	0.120539	0.233453
58	6	0	-7.506431	-1.268067	-0.066857
59	6	0	-6.314110	-1.969810	-0.316753
60	1	0	-8.489879	0.614340	0.398206
61	1	0	-6.345826	-3.022214	-0.558109
62	6	0	-4.374543	2.074665	0.361797
63	6	0	-5.812009	2.144721	0.500818
64	6	0	-3.620418	3.248193	0.480657
65	6	0	-6.444918	3.359937	0.768407
66	6	0	-5.669674	4.505480	0.890868
67	6	0	-4.273375	4.446103	0.742678
68	1	0	-3.692174	5.356189	0.833131
69	1	0	-7.522289	3.406775	0.873736
70	1	0	-2.545652	3.234851	0.360395
71	7	0	-3.792865	-1.531069	-0.436535
72	6	0	-3.117366	-0.355285	-0.229171
73	1	0	-6.144572	5.456839	1.097333
74	6	0	-8.768937	-2.052732	-0.143995
75	8	0	-8.823649	-3.240447	-0.397520
76	8	0	-9.856863	-1.304414	0.098234
77	6	0	-11.129420	-1.982109	0.043131
78	1	0	-11.871388	-1.218329	0.259622
79	1	0	-11.289624	-2.402410	-0.949619
80	1	0	-11.166356	-2.775405	0.789620

19·Ba(ClO_4)$_2$ (S_0) BHandH

Center Number	Atomic Number	Atomic Type	Coordinates (Angstroms) X	Y	Z
1	6	0	-0.149897	2.014101	-1.763257
2	6	0	0.200791	2.976041	-0.829467
3	6	0	0.840142	1.527551	-2.596874
4	6	0	1.515950	3.290819	-0.610868
5	1	0	-0.556326	3.378939	-0.171103
6	6	0	2.153851	1.878086	-2.416930
7	1	0	0.589643	0.780022	-3.337758
8	6	0	2.537214	2.670409	-1.336511
9	1	0	1.745846	3.974865	0.190250

10	1	0	2.889094	1.434165	-3.064461
11	6	0	2.929161	-3.082762	1.740062
12	1	0	3.132403	-2.761949	2.769560
13	1	0	3.557418	-3.955880	1.529181
14	6	0	1.503012	-3.477223	1.551046
15	1	0	1.250075	-4.293800	2.234077
16	1	0	1.361500	-3.813151	0.525686
17	6	0	0.189075	-2.152401	3.035681
18	1	0	0.177567	-3.078334	3.618423
19	1	0	-0.827001	-1.782508	2.944108
20	6	0	1.025033	-1.124467	3.739428
21	1	0	1.980211	-1.530275	4.095004
22	1	0	0.459050	-0.761368	4.603572
23	6	0	1.791321	1.049462	3.511333
24	1	0	2.682988	0.759825	4.082352
25	1	0	1.038817	1.448576	4.200788
26	6	0	2.137690	2.090245	2.496475
27	1	0	2.607681	2.937794	3.003206
28	1	0	1.224313	2.439961	2.016167
29	6	0	4.232424	2.175905	1.370133
30	1	0	4.935257	1.402645	1.073529
31	1	0	4.559425	2.606754	2.323466
32	6	0	4.215885	3.256239	0.315672
33	1	0	5.219319	3.685237	0.262889
34	1	0	3.565321	4.075654	0.617501
35	6	0	4.873404	2.167707	-1.776090
36	1	0	5.821865	2.520822	-1.372371
37	1	0	4.825234	2.531473	-2.807788
38	6	0	4.910288	0.651629	-1.829587
39	1	0	5.929677	0.368655	-2.124567
40	1	0	4.234492	0.225600	-2.572960
41	6	0	5.007833	-1.223882	-0.507569
42	1	0	4.567298	-1.816558	-1.313348
43	1	0	6.101581	-1.279782	-0.589464
44	6	0	4.607823	-1.799615	0.802609
45	1	0	5.151303	-2.741652	0.926513
46	1	0	4.894148	-1.129580	1.622828
47	7	0	3.850909	2.796544	-0.991728
48	8	0	2.995841	1.545989	1.532970
49	8	0	1.278488	-0.071194	2.863654
50	8	0	0.631695	-2.404291	1.734813
51	8	0	3.237240	-2.055176	0.851061
52	8	0	4.607708	0.106191	-0.590896
53	56	0	1.089211	-0.239603	0.108641
54	6	0	-2.676090	1.684272	-1.296407
55	6	0	-2.508648	-0.534861	-1.341339
56	6	0	-4.589465	0.582727	-0.702654
57	6	0	-3.092899	-1.768330	-1.118482
58	1	0	-2.551906	-2.694573	-1.243958
59	6	0	-5.176134	-0.624317	-0.476992
60	1	0	-6.190009	-0.718009	-0.122490
61	6	0	-4.407582	-1.781391	-0.699124
62	6	0	-4.835378	2.000354	-0.599913
63	6	0	-3.649045	2.680883	-0.966608
64	6	0	-5.954031	2.705617	-0.209474
65	1	0	-6.859437	2.187980	0.076999
66	6	0	-3.606707	4.060795	-0.938455
67	6	0	-4.730866	4.744485	-0.546978
68	1	0	-4.712617	5.825342	-0.518831
69	6	0	-5.893729	4.076770	-0.185917
70	1	0	-2.706499	4.590223	-1.219529
71	7	0	-3.317464	0.517317	-1.134453
72	1	0	-6.760123	4.645994	0.120044

73	7	0	-1.301876	-0.065526	-1.656715
74	6	0	-1.390395	1.280953	-1.634669
75	6	0	-4.997896	-3.106935	-0.456468
76	8	0	-4.418035	-4.138890	-0.590691
77	8	0	-6.254954	-3.041050	-0.061325
78	6	0	-6.866326	-4.278524	0.192306
79	1	0	-7.881831	-4.057051	0.502577
80	1	0	-6.866471	-4.894655	-0.704921
81	1	0	-6.334739	-4.812196	0.977963
82	17	0	1.952614	-2.502399	-2.283408
83	17	0	-1.673873	0.808913	2.029232
84	8	0	3.040705	-3.401182	-2.008885
85	8	0	2.433322	-1.113991	-2.161787
86	8	0	0.925090	-2.637089	-1.248380
87	8	0	1.403067	-2.721789	-3.578429
88	8	0	-1.459410	-0.460218	1.334304
89	8	0	-0.668081	1.720122	1.476393
90	8	0	-1.448203	0.635123	3.440957
91	8	0	-2.990076	1.300872	1.766184

19·Ba(ClO_4)$_2$ (S_1) BHandH

Center Number	Atomic Number	Atomic Type	Coordinates (Angstroms) X	Y	Z
1	6	0	-0.368426	2.475186	-1.322086
2	6	0	0.007083	3.208431	-0.187168
3	6	0	0.587005	2.292291	-2.327565
4	6	0	1.332887	3.551111	0.025808
5	1	0	-0.718324	3.387760	0.595399
6	6	0	1.905101	2.683499	-2.143247
7	1	0	0.317807	1.756979	-3.229236
8	6	0	2.338264	3.185391	-0.898411
9	1	0	1.589477	4.017927	0.964546
10	1	0	2.606258	2.494443	-2.939017
11	6	0	3.516740	-3.323247	0.977044
12	1	0	3.855573	-3.195775	2.013850
13	1	0	4.148334	-4.087376	0.504955
14	6	0	2.079447	-3.795503	0.902631
15	1	0	1.977693	-4.748327	1.434557
16	1	0	1.800208	-3.935651	-0.141146
17	6	0	0.866924	-2.880523	2.819296
18	1	0	0.993658	-3.898410	3.204028
19	1	0	-0.176652	-2.588473	2.909043
20	6	0	1.729843	-1.918875	3.620258
21	1	0	2.771885	-2.257306	3.697130
22	1	0	1.315194	-1.845492	4.633826
23	6	0	2.262081	0.397521	3.764418
24	1	0	3.304264	0.149590	4.012255
25	1	0	1.694057	0.518451	4.695660
26	6	0	2.187237	1.676462	2.955573
27	1	0	2.611583	2.505268	3.530733
28	1	0	1.145020	1.900331	2.729439
29	6	0	4.163797	2.134724	1.610213
30	1	0	4.819690	1.419633	1.119946
31	1	0	4.553646	2.350954	2.612403
32	6	0	4.122286	3.430723	0.793512
33	1	0	5.134644	3.846883	0.784747
34	1	0	3.496713	4.173981	1.288576
35	6	0	4.699953	2.821544	-1.545685
36	1	0	5.667803	3.084888	-1.115774

37	1	0	4.604288	3.401388	-2.471972
38	6	0	4.742387	1.342782	-1.956631
39	1	0	5.674091	1.208594	-2.526214
40	1	0	3.914408	1.054433	-2.605036
41	6	0	5.136778	-0.838204	-1.172796
42	1	0	4.525351	-1.217013	-1.994984
43	1	0	6.190471	-0.831098	-1.489065
44	6	0	5.008316	-1.756066	0.016156
45	1	0	5.563035	-2.672984	-0.215357
46	1	0	5.453091	-1.301133	0.911453
47	7	0	3.690326	3.266287	-0.589369
48	8	0	2.877575	1.512828	1.711205
49	8	0	1.694542	-0.647551	2.984131
50	8	0	1.153735	-2.842178	1.419565
51	8	0	3.643379	-2.092889	0.268697
52	8	0	4.745021	0.485576	-0.821853
53	56	0	1.157387	-0.358529	0.155087
54	6	0	-2.919030	1.922261	-1.028762
55	6	0	-2.579515	-0.308866	-1.209843
56	6	0	-4.803868	0.622642	-0.627794
57	6	0	-3.082611	-1.603549	-1.089246
58	1	0	-2.453006	-2.471005	-1.223522
59	6	0	-5.306034	-0.654156	-0.506281
60	1	0	-6.326929	-0.836403	-0.208671
61	6	0	-4.433012	-1.752252	-0.747529
62	6	0	-5.162811	2.020662	-0.426225
63	6	0	-3.991216	2.823834	-0.671977
64	6	0	-6.360100	2.623408	-0.044090
65	1	0	-7.242428	2.023611	0.146205
66	6	0	-4.054873	4.210533	-0.526707
67	6	0	-5.260045	4.787955	-0.145947
68	1	0	-5.321801	5.863753	-0.029545
69	6	0	-6.400991	4.005629	0.093637
70	1	0	-3.179479	4.822333	-0.708262
71	7	0	-3.491748	0.684811	-1.000587
72	1	0	-7.324281	4.487617	0.391897
73	7	0	-1.372540	0.278298	-1.402050
74	6	0	-1.569903	1.637069	-1.302241
75	6	0	-4.926981	-3.149285	-0.601598
76	8	0	-4.250614	-4.139999	-0.753656
77	8	0	-6.238199	-3.199459	-0.272753
78	6	0	-6.784196	-4.517769	-0.104180
79	1	0	-7.829933	-4.367763	0.154709
80	1	0	-6.693616	-5.088302	-1.029387
81	1	0	-6.261397	-5.046311	0.693955
82	17	0	1.732565	-1.977750	-2.852297
83	17	0	-1.632866	0.117351	2.289092
84	8	0	2.929309	-2.842321	-2.991287
85	8	0	2.176615	-0.574609	-2.444474
86	8	0	0.878311	-2.465667	-1.697289
87	8	0	0.952136	-1.922026	-4.094278
88	8	0	-1.240104	-1.134499	1.534580
89	8	0	-0.686211	1.197084	1.787130
90	8	0	-1.434920	-0.086913	3.743253
91	8	0	-3.023865	0.488427	1.974072

----

19 (S_0) BHandHLYP

Center Number	Atomic Number	Atomic Type	Coordinates (Angstroms) X	Y	Z
1	6	0	-1.740312	-0.251948	-0.107915
2	6	0	-1.061080	0.709329	0.634134
3	6	0	-0.965857	-1.139894	-0.852073
4	6	0	0.313342	0.800380	0.626246
5	1	0	-1.611082	1.394340	1.254983
6	6	0	0.408008	-1.063923	-0.871180
7	1	0	-1.454922	-1.906244	-1.426871
8	6	0	1.099612	-0.082081	-0.136084
9	1	0	0.770447	1.571794	1.215026
10	1	0	0.945073	-1.786734	-1.454547
11	6	0	8.231418	-2.024162	1.372134
12	1	0	8.037370	-2.477395	0.400883
13	1	0	8.797382	-2.740718	1.970196
14	6	0	9.043965	-0.764204	1.198798
15	1	0	10.056082	-1.023418	0.893924
16	1	0	9.103575	-0.247510	2.151370
17	6	0	8.942098	0.027580	-1.040895
18	1	0	9.121277	-1.011804	-1.314729
19	1	0	9.885586	0.566352	-1.131961
20	6	0	7.922141	0.583874	-1.995613
21	1	0	6.989184	0.042514	-1.862772
22	1	0	8.263281	0.419812	-3.017340
23	6	0	6.398932	2.402486	-1.930399
24	1	0	5.911613	1.885871	-2.755659
25	1	0	6.434504	3.458139	-2.173882
26	6	0	5.611730	2.178204	-0.653538
27	1	0	5.938199	2.876871	0.116779
28	1	0	5.804024	1.172594	-0.286049
29	6	0	3.394242	2.310979	0.184051
30	1	0	2.450831	2.734576	-0.140157
31	1	0	3.792317	2.939388	0.981981
32	6	0	3.184335	0.904014	0.728658
33	1	0	2.663482	0.977464	1.677255
34	1	0	4.137430	0.444208	0.954477
35	6	0	3.265161	-0.785318	-1.073870
36	1	0	4.137907	-0.202312	-1.339579
37	1	0	2.717965	-0.943896	-1.996076
38	6	0	3.730737	-2.126362	-0.542167
39	1	0	4.256869	-2.648020	-1.343303
40	1	0	2.888514	-2.746087	-0.233858
41	6	0	5.311642	-3.056674	0.937370
42	1	0	4.631396	-3.877856	1.170442
43	1	0	5.955305	-3.386054	0.121106
44	6	0	6.111418	-2.744195	2.180648
45	1	0	5.432488	-2.434497	2.967662
46	1	0	6.618650	-3.651947	2.509370
47	7	0	2.466624	0.005770	-0.157577
48	8	0	4.247366	2.367435	-0.938344
49	8	0	7.728700	1.964977	-1.778862
50	8	0	8.456939	0.124855	0.276126
51	8	0	7.031480	-1.692410	2.026111
52	8	0	4.594685	-1.914910	0.546948
53	6	0	-5.137749	-1.272564	-0.323714
54	6	0	-4.137787	0.654236	0.211404
55	7	0	-5.313080	0.010625	0.065222
56	6	0	-6.443752	0.729269	0.260813
57	6	0	-7.610284	0.036470	0.074109
58	6	0	-7.523523	-1.322627	-0.308618

59	6	0	-6.317577	-1.984209	-0.514583
60	1	0	-8.567783	0.500656	0.202964
61	1	0	-6.310396	-3.012968	-0.819555
62	6	0	-4.517438	2.007897	0.549016
63	6	0	-5.944498	2.049284	0.590179
64	6	0	-3.795674	3.172286	0.799000
65	6	0	-6.614551	3.227928	0.891676
66	6	0	-5.876670	4.364345	1.143659
67	6	0	-4.480731	4.332736	1.091975
68	1	0	-3.927052	5.235549	1.284022
69	1	0	-7.689961	3.253465	0.923820
70	1	0	-2.722145	3.179924	0.757037
71	7	0	-3.822803	-1.503014	-0.425354
72	6	0	-3.192353	-0.347484	-0.101608
73	1	0	-6.378032	5.286909	1.378023
74	6	0	-8.761458	-2.114605	-0.525198
75	8	0	-8.769424	-3.270314	-0.853370
76	8	0	-9.863074	-1.413036	-0.319157
77	6	0	-11.107142	-2.086949	-0.504531
78	1	0	-11.869306	-1.354530	-0.286827
79	1	0	-11.196851	-2.433575	-1.525854
80	1	0	-11.183834	-2.925911	0.174732

19 (S_1) BHandHLYP

Center Number	Atomic Number	Atomic Type	Coordinates (Angstroms) X	Y	Z
1	6	0	-1.670601	-0.277856	-0.340993
2	6	0	-0.937972	0.762678	0.250155
3	6	0	-0.943248	-1.257489	-1.037027
4	6	0	0.439523	0.840017	0.143243
5	1	0	-1.451792	1.520082	0.827902
6	6	0	0.433292	-1.194834	-1.154329
7	1	0	-1.477029	-2.083558	-1.489820
8	6	0	1.176377	-0.135975	-0.572649
9	1	0	0.942780	1.675535	0.607648
10	1	0	0.937314	-1.988619	-1.686491
11	6	0	7.227887	-1.300849	2.702754
12	1	0	7.721928	-2.069992	2.100645
13	1	0	7.357031	-1.578490	3.757965
14	6	0	7.875639	0.051127	2.458460
15	1	0	8.909963	0.028975	2.826035
16	1	0	7.334059	0.819826	3.014030
17	6	0	8.793775	-0.162827	0.246055
18	1	0	8.993936	-1.195776	0.555515
19	1	0	9.739987	0.392025	0.297126
20	6	0	8.275218	-0.200494	-1.177424
21	1	0	7.304038	-0.703828	-1.195298
22	1	0	8.977780	-0.779998	-1.787260
23	6	0	6.866120	1.466022	-2.197967
24	1	0	6.410589	0.637572	-2.754252
25	1	0	6.995396	2.304474	-2.885121
26	6	0	5.965776	1.854537	-1.033628
27	1	0	6.331217	2.774382	-0.557922
28	1	0	5.992188	1.061285	-0.285050
29	6	0	3.678069	2.170219	-0.482793
30	1	0	2.788846	2.609185	-0.938583
31	1	0	4.047032	2.858890	0.289570
32	6	0	3.335068	0.824689	0.159894
33	1	0	2.799358	1.000571	1.093530

34	1	0	4.247953	0.293366	0.430989
35	6	0	3.273993	-0.912440	-1.625646
36	1	0	4.087908	-0.324547	-2.052337
37	1	0	2.617092	-1.179318	-2.452377
38	6	0	3.862865	-2.174272	-1.005143
39	1	0	4.032879	-2.933250	-1.780820
40	1	0	3.169388	-2.589218	-0.264567
41	6	0	5.609808	-2.799889	0.518173
42	1	0	5.222865	-3.798008	0.281000
43	1	0	6.693366	-2.830840	0.381214
44	6	0	5.260105	-2.450928	1.959254
45	1	0	4.180094	-2.320861	2.057193
46	1	0	5.564437	-3.273822	2.620168
47	7	0	2.544214	-0.060686	-0.691740
48	8	0	4.643620	2.055227	-1.529427
49	8	0	8.169879	1.110604	-1.745822
50	8	0	7.828351	0.449989	1.093594
51	8	0	5.841749	-1.221082	2.384315
52	8	0	5.107398	-1.829591	-0.396154
53	6	0	-5.108171	-1.271110	-0.254755
54	6	0	-4.034806	0.691823	0.090325
55	7	0	-5.236493	0.053212	0.069342
56	6	0	-6.351043	0.811353	0.295644
57	6	0	-7.544926	0.120539	0.233453
58	6	0	-7.506431	-1.268067	-0.066857
59	6	0	-6.314110	-1.969810	-0.316753
60	1	0	-8.489879	0.614340	0.398206
61	1	0	-6.345826	-3.022214	-0.558109
62	6	0	-4.374543	2.074665	0.361797
63	6	0	-5.812009	2.144721	0.500818
64	6	0	-3.620418	3.248193	0.480657
65	6	0	-6.444918	3.359937	0.768407
66	6	0	-5.669674	4.505480	0.890868
67	6	0	-4.273375	4.446103	0.742678
68	1	0	-3.692174	5.356189	0.833131
69	1	0	-7.522289	3.406775	0.873736
70	1	0	-2.545652	3.234851	0.360395
71	7	0	-3.792865	-1.531069	-0.436535
72	6	0	-3.117366	-0.355285	-0.229171
73	1	0	-6.144572	5.456839	1.097333
74	6	0	-8.768937	-2.052732	-0.143995
75	8	0	-8.823649	-3.240447	-0.397520
76	8	0	-9.856863	-1.304414	0.098234
77	6	0	-11.129420	-1.982109	0.043131
78	1	0	-11.871388	-1.218329	0.259622
79	1	0	-11.289624	-2.402410	-0.949619
80	1	0	-11.166356	-2.775405	0.789620

19·Ba(ClO$_4$)$_2$ (S$_0$) BHandHLYP

Center Number	Atomic Number	Atomic Type	Coordinates (Angstroms) X	Y	Z
1	6	0	-0.544642	2.401384	-0.985180
2	6	0	-0.100200	3.086593	0.141253
3	6	0	0.329842	2.285293	-2.055736
4	6	0	1.214767	3.479064	0.256139
5	1	0	-0.759209	3.212102	0.981655
6	6	0	1.638417	2.712906	-1.962380
7	1	0	0.010666	1.785339	-2.953125
8	6	0	2.148669	3.202403	-0.755200

9	1	0	1.524731	3.922542	1.181364
10	1	0	2.273488	2.565121	-2.810526
11	6	0	3.975807	-3.181724	0.530325
12	1	0	4.395114	-3.085724	1.532960
13	1	0	4.599154	-3.884271	-0.024218
14	6	0	2.576455	-3.742922	0.559417
15	1	0	2.591861	-4.725767	1.026801
16	1	0	2.211618	-3.843348	-0.454226
17	6	0	1.455762	-3.090175	2.612327
18	1	0	1.687061	-4.115011	2.894715
19	1	0	0.406356	-2.904110	2.792139
20	6	0	2.283647	-2.133360	3.438580
21	1	0	3.345601	-2.380035	3.408064
22	1	0	1.950733	-2.192668	4.475019
23	6	0	2.691358	0.171597	3.717477
24	1	0	3.762077	-0.022678	3.807429
25	1	0	2.255155	0.182002	4.716511
26	6	0	2.441694	1.498181	3.047380
27	1	0	2.879606	2.296766	3.641855
28	1	0	1.374265	1.665552	2.975816
29	6	0	4.207260	2.162169	1.535839
30	1	0	4.842861	1.499173	0.967240
31	1	0	4.678356	2.363080	2.497401
32	6	0	4.046720	3.476722	0.782160
33	1	0	5.032067	3.928416	0.706760
34	1	0	3.448226	4.170876	1.360562
35	6	0	4.452271	3.107352	-1.632147
36	1	0	5.413450	3.469693	-1.285510
37	1	0	4.188673	3.719701	-2.493665
38	6	0	4.641461	1.682840	-2.150499
39	1	0	5.514140	1.710476	-2.808197
40	1	0	3.801078	1.337461	-2.741482
41	6	0	5.240590	-0.496861	-1.587543
42	1	0	4.557330	-0.834228	-2.360636
43	1	0	6.243837	-0.431122	-2.014424
44	6	0	5.272354	-1.509612	-0.478416
45	1	0	5.834453	-2.368517	-0.842829
46	1	0	5.794198	-1.109466	0.391962
47	7	0	3.508616	3.355896	-0.558541
48	8	0	2.969206	1.499022	1.734297
49	8	0	2.090807	-0.833262	2.938204
50	8	0	1.653413	-2.905365	1.224101
51	8	0	3.974505	-1.931338	-0.116200
52	8	0	4.858972	0.770324	-1.104748
53	56	0	1.141479	-0.376685	0.159731
54	6	0	-3.095291	1.887673	-0.721745
55	6	0	-2.800948	-0.321420	-0.914192
56	6	0	-5.021181	0.643594	-0.492201
57	6	0	-3.348650	-1.600970	-0.895476
58	1	0	-2.745828	-2.477569	-1.036062
59	6	0	-5.570242	-0.607240	-0.470936
60	1	0	-6.615815	-0.766042	-0.296410
61	6	0	-4.712805	-1.716589	-0.677006
62	6	0	-5.366348	2.047880	-0.339550
63	6	0	-4.174745	2.815490	-0.482398
64	6	0	-6.579542	2.673281	-0.094298
65	1	0	-7.481589	2.096651	0.017413
66	6	0	-4.225873	4.199126	-0.379125
67	6	0	-5.443076	4.801003	-0.136736
68	1	0	-5.495826	5.873060	-0.054913
69	6	0	-6.609697	4.048480	0.005852
70	1	0	-3.333268	4.789939	-0.487331
71	7	0	-3.687906	0.675016	-0.718955

72	1	0	-7.542787	4.549178	0.196680
73	7	0	-1.586247	0.232575	-1.051950
74	6	0	-1.755970	1.577822	-0.943488
75	6	0	-5.263147	-3.096854	-0.656695
76	8	0	-4.612224	-4.085131	-0.821988
77	8	0	-6.575797	-3.122715	-0.432875
78	6	0	-7.178575	-4.409281	-0.391207
79	1	0	-8.227763	-4.235193	-0.201800
80	1	0	-7.042464	-4.921490	-1.335617
81	1	0	-6.743137	-5.004803	0.401541
82	17	0	1.681502	-1.769142	-2.990116
83	17	0	-1.419470	-0.247299	2.566290
84	8	0	2.883230	-2.520188	-3.301988
85	8	0	2.058098	-0.402602	-2.527810
86	8	0	0.990171	-2.380296	-1.828526
87	8	0	0.798907	-1.684598	-4.124881
88	8	0	-0.987972	-1.404857	1.745930
89	8	0	-0.566091	0.884493	2.115354
90	8	0	-1.176787	-0.518466	3.968888
91	8	0	-2.808929	0.056394	2.312958

19·Ba(ClO$_4$)$_2$ (S$_1$) BHandHLYP

Center Number	Atomic Number	Atomic Type	Coordinates (Angstroms) X	Y	Z
1	6	0	-0.368426	2.475186	-1.322086
2	6	0	0.007083	3.208431	-0.187168
3	6	0	0.587005	2.292291	-2.327565
4	6	0	1.332887	3.551111	0.025808
5	1	0	-0.718324	3.387760	0.595399
6	6	0	1.905101	2.683499	-2.143247
7	1	0	0.317807	1.756979	-3.229236
8	6	0	2.338264	3.185391	-0.898411
9	1	0	1.589477	4.017927	0.964546
10	1	0	2.606258	2.494443	-2.939017
11	6	0	3.516740	-3.323247	0.977044
12	1	0	3.855573	-3.195775	2.013850
13	1	0	4.148334	-4.087376	0.504955
14	6	0	2.079447	-3.795503	0.902631
15	1	0	1.977693	-4.748327	1.434557
16	1	0	1.800208	-3.935651	-0.141146
17	6	0	0.866924	-2.880523	2.819296
18	1	0	0.993658	-3.898410	3.204028
19	1	0	-0.176652	-2.588473	2.909043
20	6	0	1.729843	-1.918875	3.620258
21	1	0	2.771885	-2.257306	3.697130
22	1	0	1.315194	-1.845492	4.633826
23	6	0	2.262081	0.397521	3.764418
24	1	0	3.304264	0.149590	4.012255
25	1	0	1.694057	0.518451	4.695660
26	6	0	2.187237	1.676462	2.955573
27	1	0	2.611583	2.505268	3.530733
28	1	0	1.145020	1.900331	2.729439
29	6	0	4.163797	2.134724	1.610213
30	1	0	4.819690	1.419633	1.119946
31	1	0	4.553646	2.350954	2.612403
32	6	0	4.122286	3.430723	0.793512
33	1	0	5.134644	3.846883	0.784747
34	1	0	3.496713	4.173981	1.288576
35	6	0	4.699953	2.821544	-1.545685

36	1	0	5.667803	3.084888	-1.115774
37	1	0	4.604288	3.401388	-2.471972
38	6	0	4.742387	1.342782	-1.956631
39	1	0	5.674091	1.208594	-2.526214
40	1	0	3.914408	1.054433	-2.605036
41	6	0	5.136778	-0.838204	-1.172796
42	1	0	4.525351	-1.217013	-1.994984
43	1	0	6.190471	-0.831098	-1.489065
44	6	0	5.008316	-1.756066	0.016156
45	1	0	5.563035	-2.672984	-0.215357
46	1	0	5.453091	-1.301133	0.911453
47	7	0	3.690326	3.266287	-0.589369
48	8	0	2.877575	1.512828	1.711205
49	8	0	1.694542	-0.647551	2.984131
50	8	0	1.153735	-2.842178	1.419565
51	8	0	3.643379	-2.092889	0.268697
52	8	0	4.745021	0.485576	-0.821853
53	56	0	1.157387	-0.358529	0.155087
54	6	0	-2.919030	1.922261	-1.028762
55	6	0	-2.579515	-0.308866	-1.209843
56	6	0	-4.803868	0.622642	-0.627794
57	6	0	-3.082611	-1.603549	-1.089246
58	1	0	-2.453006	-2.471005	-1.223522
59	6	0	-5.306034	-0.654156	-0.506281
60	1	0	-6.326929	-0.836403	-0.208671
61	6	0	-4.433012	-1.752252	-0.747529
62	6	0	-5.162811	2.020662	-0.426225
63	6	0	-3.991216	2.823834	-0.671977
64	6	0	-6.360100	2.623408	-0.044090
65	1	0	-7.242428	2.023611	0.146205
66	6	0	-4.054873	4.210533	-0.526707
67	6	0	-5.260045	4.787955	-0.145947
68	1	0	-5.321801	5.863753	-0.029545
69	6	0	-6.400991	4.005629	0.093637
70	1	0	-3.179479	4.822333	-0.708262
71	7	0	-3.491748	0.684811	-1.000587
72	1	0	-7.324281	4.487617	0.391897
73	7	0	-1.372540	0.278298	-1.402050
74	6	0	-1.569903	1.637069	-1.302241
75	6	0	-4.926981	-3.149285	-0.601598
76	8	0	-4.250614	-4.139999	-0.753656
77	8	0	-6.238199	-3.199459	-0.272753
78	6	0	-6.784196	-4.517769	-0.104180
79	1	0	-7.829933	-4.367763	0.154709
80	1	0	-6.693616	-5.088302	-1.029387
81	1	0	-6.261397	-5.046311	0.693955
82	17	0	1.732565	-1.977750	-2.852297
83	17	0	-1.632866	0.117351	2.289092
84	8	0	2.929309	-2.842321	-2.991287
85	8	0	2.176615	-0.574609	-2.444474
86	8	0	0.878311	-2.465667	-1.697289
87	8	0	0.952136	-1.922026	-4.094278
88	8	0	-1.240104	-1.134499	1.534580
89	8	0	-0.686211	1.197084	1.787130
90	8	0	-1.434920	-0.086913	3.743253
91	8	0	-3.023865	0.488427	1.974072

----

19 (S_0) B3LYP

Center Number	Atomic Number	Atomic Type	Coordinates (Angstroms) X	Y	Z
1	6	0	-1.568331	-0.256182	-0.395187
2	6	0	-0.825695	0.799472	0.149469
3	6	0	-0.856799	-1.267242	-1.058554
4	6	0	0.552113	0.862001	0.029936
5	1	0	-1.332291	1.580364	0.702110
6	6	0	0.518902	-1.218361	-1.186985
7	1	0	-1.408439	-2.103554	-1.468740
8	6	0	1.273958	-0.147219	-0.649105
9	1	0	1.067421	1.710328	0.456559
10	1	0	1.013865	-2.033519	-1.695529
11	6	0	6.634488	-1.063442	2.895739
12	1	0	7.200463	-1.895954	2.461344
13	1	0	6.589561	-1.229165	3.982687
14	6	0	7.345792	0.250258	2.613423
15	1	0	8.313966	0.261173	3.134305
16	1	0	6.742823	1.074968	2.999606
17	6	0	8.569865	-0.190489	0.589452
18	1	0	8.725477	-1.179789	1.041537
19	1	0	9.500812	0.382152	0.694769
20	6	0	8.244572	-0.401667	-0.878856
21	1	0	7.272122	-0.898615	-0.961262
22	1	0	9.010005	-1.063649	-1.303478
23	6	0	7.016438	1.179442	-2.212682
24	1	0	6.514678	0.301609	-2.639183
25	1	0	7.253191	1.862162	-3.030571
26	6	0	6.093356	1.837715	-1.195519
27	1	0	6.465847	2.835385	-0.926067
28	1	0	6.088364	1.232796	-0.288433
29	6	0	3.786096	2.136551	-0.761377
30	1	0	2.902824	2.507201	-1.285285
31	1	0	4.114057	2.907319	-0.048289
32	6	0	3.451367	0.846529	-0.005525
33	1	0	2.929031	1.086750	0.921391
34	1	0	4.368462	0.340431	0.297904
35	6	0	3.345174	-0.973728	-1.704854
36	1	0	4.198240	-0.427007	-2.108138
37	1	0	2.690510	-1.195810	-2.549340
38	6	0	3.842493	-2.273292	-1.082601
39	1	0	4.037769	-3.021503	-1.864328
40	1	0	3.081141	-2.674800	-0.404655
41	6	0	5.308250	-2.842489	0.736271
42	1	0	4.891205	-3.846485	0.580364
43	1	0	6.394110	-2.950433	0.800572
44	6	0	4.749605	-2.255682	2.029563
45	1	0	3.678312	-2.071528	1.920630
46	1	0	4.886922	-2.975849	2.850160
47	7	0	2.646676	-0.098842	-0.773344
48	8	0	4.786218	1.939639	-1.756439
49	8	0	8.259908	0.811005	-1.625984
50	8	0	7.505071	0.499288	1.228885
51	8	0	5.325084	-0.999268	2.354263
52	8	0	5.053853	-2.002498	-0.377893
53	6	0	-4.997945	-1.246355	-0.263458
54	6	0	-3.929698	0.724983	0.047640
55	7	0	-5.129499	0.083387	0.048488
56	6	0	-6.243560	0.839803	0.280917
57	6	0	-7.435029	0.142635	0.240659
58	6	0	-7.393930	-1.248458	-0.046186

59	6	0	-6.202499	-1.948690	-0.303413
60	1	0	-8.383000	0.628888	0.411435
61	1	0	-6.237714	-3.003743	-0.532801
62	6	0	-4.270175	2.108117	0.306538
63	6	0	-5.706110	2.176420	0.464533
64	6	0	-3.520026	3.286762	0.397477
65	6	0	-6.336484	3.393555	0.726441
66	6	0	-5.564790	4.542796	0.823353
67	6	0	-4.171408	4.485408	0.653673
68	1	0	-3.592104	5.398962	0.721028
69	1	0	-7.412735	3.438931	0.846708
70	1	0	-2.447803	3.273151	0.254817
71	7	0	-3.686353	-1.502987	-0.457136
72	6	0	-3.014373	-0.327899	-0.269487
73	1	0	-6.039730	5.495377	1.024718
74	6	0	-8.650978	-2.042250	-0.101197
75	8	0	-8.709123	-3.226655	-0.342577
76	8	0	-9.747421	-1.291368	0.151021
77	6	0	-11.001663	-1.992452	0.113939
78	1	0	-11.758814	-1.243934	0.336246
79	1	0	-11.166007	-2.425993	-0.873286
80	1	0	-11.014901	-2.787956	0.859980

19 (S_1) B3LYP

Center Number	Atomic Number	Atomic Type	Coordinates (Angstroms) X	Y	Z
1	6	0	-1.670948	-0.378697	-0.302234
2	6	0	-0.920991	0.814243	-0.359748
3	6	0	-0.945632	-1.591469	-0.365833
4	6	0	0.430462	0.805522	-0.454104
5	1	0	-1.436045	1.761681	-0.359211
6	6	0	0.404222	-1.608349	-0.464788
7	1	0	-1.501089	-2.516469	-0.314024
8	6	0	1.153629	-0.408517	-0.507661
9	1	0	0.949167	1.748567	-0.529431
10	1	0	0.909106	-2.561889	-0.470942
11	6	0	7.098074	0.481832	2.861987
12	1	0	7.617288	-0.481043	2.874303
13	1	0	7.219599	0.923790	3.856764
14	6	0	7.713206	1.381660	1.837444
15	1	0	8.751322	1.599291	2.113584
16	1	0	7.167446	2.325249	1.814673
17	6	0	8.604366	-0.104257	0.253402
18	1	0	8.779909	-0.784275	1.095339
19	1	0	9.557210	0.387215	0.022846
20	6	0	8.143116	-0.917795	-0.911368
21	1	0	7.174654	-1.367927	-0.670128
22	1	0	8.863621	-1.721328	-1.084888
23	6	0	6.767236	-0.079801	-2.626681
24	1	0	6.321236	-1.079519	-2.695341
25	1	0	6.876019	0.304800	-3.640347
26	6	0	5.870160	0.808763	-1.820462
27	1	0	6.256182	1.834855	-1.818557
28	1	0	5.863291	0.459713	-0.786366
29	6	0	3.663831	1.484705	-1.628451
30	1	0	2.787320	1.632533	-2.259733
31	1	0	4.066024	2.470216	-1.364532
32	6	0	3.270906	0.768105	-0.359432
33	1	0	2.719830	1.446277	0.287564

34	1	0	4.161947	0.466683	0.195139
35	6	0	3.224989	-1.606220	-0.932390
36	1	0	3.993052	-1.322687	-1.654858
37	1	0	2.565717	-2.308045	-1.433300
38	6	0	3.894118	-2.264880	0.239407
39	1	0	4.106156	-3.312650	-0.001147
40	1	0	3.228864	-2.246331	1.110250
41	6	0	5.667444	-1.931562	1.723731
42	1	0	5.398543	-2.953949	2.008033
43	1	0	6.749724	-1.909816	1.582319
44	6	0	5.266893	-0.977251	2.807927
45	1	0	4.178986	-0.905803	2.851131
46	1	0	5.613440	-1.352020	3.777563
47	7	0	2.490789	-0.416969	-0.593403
48	8	0	4.594267	0.775868	-2.381323
49	8	0	8.041119	-0.145514	-2.072057
50	8	0	7.636679	0.843013	0.558956
51	8	0	5.746776	0.307744	2.571320
52	8	0	5.083877	-1.590671	0.504540
53	6	0	-5.042632	-1.263696	-0.120475
54	6	0	-3.978284	0.700656	-0.054233
55	7	0	-5.163203	0.082190	-0.022302
56	6	0	-6.258203	0.874194	0.116498
57	6	0	-7.457671	0.194820	0.150708
58	6	0	-7.422381	-1.200377	0.047424
59	6	0	-6.262950	-1.955651	-0.086379
60	1	0	-8.401407	0.704905	0.255521
61	1	0	-6.316137	-3.029460	-0.157453
62	6	0	-4.287284	2.087532	0.087278
63	6	0	-5.717987	2.181179	0.187007
64	6	0	-3.528720	3.249036	0.155798
65	6	0	-6.329357	3.418973	0.335839
66	6	0	-5.548958	4.542227	0.391273
67	6	0	-4.156586	4.458895	0.304214
68	1	0	-3.564866	5.361637	0.357609
69	1	0	-7.406242	3.491953	0.409673
70	1	0	-2.450398	3.224974	0.105213
71	7	0	-3.772572	-1.574595	-0.223877
72	6	0	-3.061375	-0.396508	-0.196225
73	1	0	-6.014999	5.511014	0.508333
74	6	0	-8.686569	-1.949815	0.082838
75	8	0	-8.760766	-3.142954	0.000311
76	8	0	-9.744703	-1.182708	0.215952
77	6	0	-10.985603	-1.843413	0.257885
78	1	0	-11.735810	-1.069509	0.374359
79	1	0	-11.153742	-2.396307	-0.663955
80	1	0	-11.021130	-2.533451	1.098321

19·Ba(ClO4)2 (S0) B3LYP

Center Number	Atomic Number	Atomic Type	Coordinates (Angstroms) X	Y	Z
1	6	0	-0.353552	2.431930	-1.417442
2	6	0	0.014407	3.221840	-0.318007
3	6	0	0.614826	2.185729	-2.397477
4	6	0	1.339929	3.566584	-0.107037
5	1	0	-0.718430	3.446547	0.445921
6	6	0	1.933010	2.577065	-2.217764
7	1	0	0.353390	1.602466	-3.271188
8	6	0	2.354226	3.145162	-0.997555

9	1	0	1.588687	4.081002	0.808798
10	1	0	2.642416	2.337651	-2.992059
11	6	0	3.487101	-3.292399	1.118429
12	1	0	3.809799	-3.127316	2.155042
13	1	0	4.124491	-4.074402	0.684958
14	6	0	2.050380	-3.765558	1.038835
15	1	0	1.939232	-4.698448	1.603212
16	1	0	1.787045	-3.943196	-0.003323
17	6	0	0.809727	-2.781609	2.902665
18	1	0	0.926193	-3.785859	3.324676
19	1	0	-0.233801	-2.482961	2.965903
20	6	0	1.664354	-1.795687	3.682995
21	1	0	2.702043	-2.138098	3.793562
22	1	0	1.230318	-1.680622	4.684381
23	6	0	2.206205	0.521627	3.752887
24	1	0	3.243365	0.281175	4.027674
25	1	0	1.621284	0.674456	4.668725
26	6	0	2.147211	1.772550	2.900141
27	1	0	2.567912	2.618676	3.452290
28	1	0	1.109344	1.993008	2.652585
29	6	0	4.143805	2.175166	1.567593
30	1	0	4.802978	1.440969	1.111322
31	1	0	4.520259	2.423923	2.567309
32	6	0	4.119235	3.441988	0.706122
33	1	0	5.133415	3.853527	0.694830
34	1	0	3.490903	4.204342	1.167347
35	6	0	4.722917	2.755911	-1.605750
36	1	0	5.685155	3.042144	-1.177847
37	1	0	4.630439	3.300304	-2.553549
38	6	0	4.779913	1.262584	-1.961522
39	1	0	5.725298	1.111243	-2.503683
40	1	0	3.968676	0.947486	-2.618701
41	6	0	5.149793	-0.890136	-1.092916
42	1	0	4.550818	-1.296371	-1.911186
43	1	0	6.208737	-0.898153	-1.391103
44	6	0	4.997791	-1.764182	0.126030
45	1	0	5.553871	-2.690023	-0.062811
46	1	0	5.428468	-1.278427	1.011975
47	7	0	3.702664	3.228788	-0.674769
48	8	0	2.852629	1.563931	1.670972
49	8	0	1.651597	-0.549231	2.998444
50	8	0	1.117785	-2.793661	1.506885
51	8	0	3.627905	-2.089228	0.367182
52	8	0	4.757537	0.446822	-0.797012
53	56	0	1.148274	-0.356373	0.159304
54	6	0	-2.899719	1.895519	-1.076194
55	6	0	-2.577402	-0.338820	-1.250991
56	6	0	-4.787291	0.611009	-0.642054
57	6	0	-3.087305	-1.629228	-1.115299
58	1	0	-2.465308	-2.501646	-1.253421
59	6	0	-5.296364	-0.661822	-0.507114
60	1	0	-6.314427	-0.835497	-0.195060
61	6	0	-4.434067	-1.766956	-0.753804
62	6	0	-5.133820	2.012233	-0.442834
63	6	0	-3.960348	2.806067	-0.709387
64	6	0	-6.321150	2.625083	-0.046041
65	1	0	-7.204621	2.032310	0.160285
66	6	0	-4.012445	4.193898	-0.569895
67	6	0	-5.207842	4.781424	-0.174320
68	1	0	-5.260516	5.858166	-0.062189
69	6	0	-6.350536	4.008136	0.085672
70	1	0	-3.135794	4.798951	-0.767367
71	7	0	-3.479858	0.662185	-1.032712

72	1	0	-7.265962	4.497914	0.395272
73	7	0	-1.369687	0.238947	-1.466315
74	6	0	-1.556277	1.599374	-1.368165
75	6	0	-4.935331	-3.159685	-0.593137
76	8	0	-4.267299	-4.155825	-0.746918
77	8	0	-6.242960	-3.199271	-0.248771
78	6	0	-6.795556	-4.512880	-0.065809
79	1	0	-7.837165	-4.354390	0.204435
80	1	0	-6.719664	-5.089553	-0.988529
81	1	0	-6.266881	-5.040155	0.729293
82	17	0	1.768408	-2.082016	-2.780226
83	17	0	-1.652564	0.212951	2.260626
84	8	0	2.963156	-2.955629	-2.870566
85	8	0	2.212073	-0.667479	-2.413194
86	8	0	0.893580	-2.526552	-1.623075
87	8	0	1.008106	-2.065432	-4.035715
88	8	0	-1.275708	-1.062792	1.538667
89	8	0	-0.706655	1.271315	1.714240
90	8	0	-1.436229	0.051104	3.718006
91	8	0	-3.045695	0.581792	1.954084

19·Ba(ClO$_4$)$_2$ (S$_1$) B3LYP

Center Number	Atomic Number	Atomic Type	Coordinates (Angstroms) X	Y	Z
1	6	0	-0.128717	1.793809	-1.927706
2	6	0	0.221319	2.914295	-1.163325
3	6	0	0.876584	1.193148	-2.692979
4	6	0	1.531649	3.237201	-0.975827
5	1	0	-0.538243	3.424491	-0.586742
6	6	0	2.183030	1.559209	-2.550187
7	1	0	0.619149	0.363201	-3.337087
8	6	0	2.561505	2.498084	-1.585375
9	1	0	1.762560	4.041627	-0.295564
10	1	0	2.923049	1.033069	-3.127097
11	6	0	2.888856	-2.935069	2.023993
12	1	0	3.083868	-2.513046	3.017881
13	1	0	3.516298	-3.826094	1.906968
14	6	0	1.462709	-3.342885	1.865389
15	1	0	1.201804	-4.079451	2.631071
16	1	0	1.326106	-3.786954	0.881615
17	6	0	0.138157	-1.862777	3.188741
18	1	0	0.103428	-2.724289	3.861982
19	1	0	-0.870558	-1.489382	3.044318
20	6	0	0.982303	-0.782181	3.797168
21	1	0	1.925299	-1.164961	4.206024
22	1	0	0.410298	-0.323783	4.609878
23	6	0	1.771492	1.352115	3.371234
24	1	0	2.657487	1.118868	3.975781
25	1	0	1.009342	1.810086	4.011320
26	6	0	2.126540	2.295116	2.268253
27	1	0	2.591049	3.186115	2.699576
28	1	0	1.218961	2.596982	1.746498
29	6	0	4.232133	2.278846	1.165980
30	1	0	4.943738	1.486180	0.954852
31	1	0	4.543898	2.804842	2.075733
32	6	0	4.221774	3.253303	0.011919
33	1	0	5.222559	3.681841	-0.077312
34	1	0	3.558875	4.089181	0.227224

35	6	0	4.903269	1.958450	-1.949024
36	1	0	5.843746	2.366857	-1.580925
37	1	0	4.852585	2.209320	-3.013429
38	6	0	4.960672	0.444634	-1.838804
39	1	0	5.991266	0.148839	-2.076768
40	1	0	4.310246	-0.066763	-2.550313
41	6	0	5.027390	-1.284586	-0.329390
42	1	0	4.615752	-1.955083	-1.087554
43	1	0	6.123422	-1.344100	-0.366161
44	6	0	4.580449	-1.731169	1.015946
45	1	0	5.128628	-2.648001	1.254697
46	1	0	4.827509	-0.978309	1.774796
47	7	0	3.867978	2.655959	-1.241633
48	8	0	2.996633	1.663635	1.369572
49	8	0	1.267229	0.175313	2.824188
50	8	0	0.593912	-2.252940	1.926605
51	8	0	3.211710	-2.001357	1.040515
52	8	0	4.631453	0.029916	-0.558378
53	56	0	1.069255	-0.275473	0.114331
54	6	0	-2.623028	1.579464	-1.293831
55	6	0	-2.575713	-0.649452	-1.360517
56	6	0	-4.586720	0.590503	-0.676997
57	6	0	-3.223161	-1.861693	-1.138765
58	1	0	-2.747235	-2.816431	-1.288794
59	6	0	-5.246464	-0.604342	-0.448403
60	1	0	-6.261253	-0.648182	-0.090320
61	6	0	-4.541157	-1.786121	-0.686282
62	6	0	-4.750606	2.001450	-0.595756
63	6	0	-3.516376	2.624464	-0.992676
64	6	0	-5.810474	2.795622	-0.211157
65	1	0	-6.746628	2.354930	0.103110
66	6	0	-3.390719	4.002131	-1.000781
67	6	0	-4.464220	4.769408	-0.615421
68	1	0	-4.385411	5.846717	-0.614154
69	6	0	-5.657077	4.165671	-0.225479
70	1	0	-2.464141	4.466578	-1.311721
71	7	0	-3.317295	0.440553	-1.113918
72	1	0	-6.486783	4.789909	0.077086
73	7	0	-1.352049	-0.249504	-1.733234
74	6	0	-1.337739	1.096530	-1.709516
75	6	0	-5.208587	-3.076577	-0.444451
76	8	0	-4.689913	-4.138484	-0.597883
77	8	0	-6.456942	-2.947627	-0.034396
78	6	0	-7.129983	-4.152918	0.211519
79	1	0	-8.129086	-3.881855	0.535796
80	1	0	-7.172789	-4.758119	-0.692343
81	1	0	-6.619646	-4.724581	0.984727
82	17	0	2.022878	-2.746003	-2.018601
83	17	0	-1.662734	1.039341	1.952640
84	8	0	3.100065	-3.614628	-1.628345
85	8	0	2.501957	-1.351265	-2.012036
86	8	0	0.963659	-2.781457	-1.008217
87	8	0	1.514035	-3.084371	-3.304435
88	8	0	-1.457099	-0.266390	1.318688
89	8	0	-0.700018	1.933333	1.304282
90	8	0	-1.372586	0.947282	3.360965
91	8	0	-2.998450	1.491334	1.725213

19(S_0) CAM-B3LYP

Center Number	Atomic Number	Atomic Type	Coordinates (Angstroms) X	Y	Z
1	6	0	-1.742338	-0.261580	-0.106584
2	6	0	-1.063162	0.695902	0.647273
3	6	0	-0.966508	-1.143510	-0.862756
4	6	0	0.315192	0.789403	0.638792
5	1	0	-1.619033	1.377424	1.279216
6	6	0	0.411378	-1.065717	-0.881892
7	1	0	-1.460350	-1.908889	-1.448138
8	6	0	1.103228	-0.087058	-0.135674
9	1	0	0.775890	1.560584	1.238068
10	1	0	0.953618	-1.786979	-1.475514
11	6	0	8.252466	-2.032883	1.362801
12	1	0	8.044503	-2.469760	0.379141
13	1	0	8.818632	-2.770134	1.948306
14	6	0	9.074889	-0.773219	1.205113
15	1	0	10.092488	-1.033951	0.895149
16	1	0	9.135387	-0.264217	2.169342
17	6	0	8.958212	0.023111	-1.042738
18	1	0	9.127595	-1.026731	-1.309878
19	1	0	9.909592	0.558660	-1.152385
20	6	0	7.925059	0.578003	-1.988752
21	1	0	6.983158	0.044913	-1.831331
22	1	0	8.248063	0.399550	-3.021422
23	6	0	6.411372	2.417054	-1.932051
24	1	0	5.919022	1.899072	-2.762791
25	1	0	6.453716	3.478973	-2.176488
26	6	0	5.621110	2.193219	-0.651991
27	1	0	5.942486	2.900648	0.122313
28	1	0	5.818005	1.182670	-0.278452
29	6	0	3.394216	2.316088	0.189441
30	1	0	2.442006	2.736475	-0.136009
31	1	0	3.795003	2.951888	0.989720
32	6	0	3.194371	0.903447	0.732903
33	1	0	2.674490	0.969539	1.690363
34	1	0	4.156403	0.443279	0.954016
35	6	0	3.277157	-0.780265	-1.084964
36	1	0	4.152205	-0.187495	-1.349902
37	1	0	2.725498	-0.934948	-2.013201
38	6	0	3.748501	-2.125304	-0.559485
39	1	0	4.283408	-2.645381	-1.365369
40	1	0	2.903606	-2.754073	-0.251549
41	6	0	5.331258	-3.063616	0.928337
42	1	0	4.643347	-3.889588	1.154810
43	1	0	5.981409	-3.391135	0.106956
44	6	0	6.126573	-2.757267	2.180245
45	1	0	5.440571	-2.447067	2.970132
46	1	0	6.635989	-3.672162	2.509344
47	7	0	2.475092	0.003676	-0.158107
48	8	0	4.248011	2.376863	-0.944272
49	8	0	7.748391	1.972215	-1.780245
50	8	0	8.489249	0.137964	0.288960
51	8	0	7.053176	-1.697585	2.035967
52	8	0	4.614404	-1.910161	0.539521
53	6	0	-5.154620	-1.283447	-0.320273
54	6	0	-4.143897	0.650571	0.213027
55	7	0	-5.326160	0.007526	0.068385
56	6	0	-6.459814	0.733543	0.262646
57	6	0	-7.630455	0.041141	0.075789
58	6	0	-7.548038	-1.323028	-0.305137

59	6	0	-6.341739	-1.991083	-0.509940
60	1	0	-8.593473	0.510956	0.203389
61	1	0	-6.340303	-3.027430	-0.815121
62	6	0	-4.522694	2.010939	0.548568
63	6	0	-5.953094	2.057260	0.589833
64	6	0	-3.795047	3.176015	0.796392
65	6	0	-6.620763	3.241722	0.889338
66	6	0	-5.876695	4.379672	1.139323
67	6	0	-4.477719	4.343334	1.087540
68	1	0	-3.917142	5.250451	1.278709
69	1	0	-7.703120	3.270877	0.921740
70	1	0	-2.714347	3.178514	0.754156
71	7	0	-3.833621	-1.518111	-0.422506
72	6	0	-3.199269	-0.356950	-0.099887
73	1	0	-6.378438	5.310369	1.373089
74	6	0	-8.791850	-2.114240	-0.521704
75	8	0	-8.807171	-3.278374	-0.849969
76	8	0	-9.898691	-1.401241	-0.315476
77	6	0	-11.147823	-2.083621	-0.503920
78	1	0	-11.916532	-1.347797	-0.287626
79	1	0	-11.233394	-2.434707	-1.531300
80	1	0	-11.222837	-2.927307	0.180549

19 (S_1) CAM-B3LYP

Center Number	Atomic Number	Atomic Type	Coordinates (Angstroms) X	Y	Z
1	6	0	-1.670601	-0.277856	-0.340993
2	6	0	-0.937972	0.762678	0.250155
3	6	0	-0.943248	-1.257489	-1.037027
4	6	0	0.439523	0.840017	0.143243
5	1	0	-1.451792	1.520082	0.827902
6	6	0	0.433292	-1.194834	-1.154329
7	1	0	-1.477029	-2.083558	-1.489820
8	6	0	1.176377	-0.135975	-0.572649
9	1	0	0.942780	1.675535	0.607648
10	1	0	0.937314	-1.988619	-1.686491
11	6	0	7.227887	-1.300849	2.702754
12	1	0	7.721928	-2.069992	2.100645
13	1	0	7.357031	-1.578490	3.757965
14	6	0	7.875639	0.051127	2.458460
15	1	0	8.909963	0.028975	2.826035
16	1	0	7.334059	0.819826	3.014030
17	6	0	8.793775	-0.162827	0.246055
18	1	0	8.993936	-1.195776	0.555515
19	1	0	9.739987	0.392025	0.297126
20	6	0	8.275218	-0.200494	-1.177424
21	1	0	7.304038	-0.703828	-1.195298
22	1	0	8.977780	-0.779998	-1.787260
23	6	0	6.866120	1.466022	-2.197967
24	1	0	6.410589	0.637572	-2.754252
25	1	0	6.995396	2.304474	-2.885121
26	6	0	5.965776	1.854537	-1.033628
27	1	0	6.331217	2.774382	-0.557922
28	1	0	5.992188	1.061285	-0.285050
29	6	0	3.678069	2.170219	-0.482793
30	1	0	2.788846	2.609185	-0.938583
31	1	0	4.047032	2.858890	0.289570
32	6	0	3.335068	0.824689	0.159894
33	1	0	2.799358	1.000571	1.093530
34	1	0	4.247953	0.293366	0.430989

35	6	0	3.273993	-0.912440	-1.625646
36	1	0	4.087908	-0.324547	-2.052337
37	1	0	2.617092	-1.179318	-2.452377
38	6	0	3.862865	-2.174272	-1.005143
39	1	0	4.032879	-2.933250	-1.780820
40	1	0	3.169388	-2.589218	-0.264567
41	6	0	5.609808	-2.799889	0.518173
42	1	0	5.222865	-3.798008	0.281000
43	1	0	6.693366	-2.830840	0.381214
44	6	0	5.260105	-2.450928	1.959254
45	1	0	4.180094	-2.320861	2.057193
46	1	0	5.564437	-3.273822	2.620168
47	7	0	2.544214	-0.060686	-0.691740
48	8	0	4.643620	2.055227	-1.529427
49	8	0	8.169879	1.110604	-1.745822
50	8	0	7.828351	0.449989	1.093594
51	8	0	5.841749	-1.221082	2.384315
52	8	0	5.107398	-1.829591	-0.396154
53	6	0	-5.108171	-1.271110	-0.254755
54	6	0	-4.034806	0.691823	0.090325
55	7	0	-5.236493	0.053212	0.069342
56	6	0	-6.351043	0.811353	0.295644
57	6	0	-7.544926	0.120539	0.233453
58	6	0	-7.506431	-1.268067	-0.066857
59	6	0	-6.314110	-1.969810	-0.316753
60	1	0	-8.489879	0.614340	0.398206
61	1	0	-6.345826	-3.022214	-0.558109
62	6	0	-4.374543	2.074665	0.361797
63	6	0	-5.812009	2.144721	0.500818
64	6	0	-3.620418	3.248193	0.480657
65	6	0	-6.444918	3.359937	0.768407
66	6	0	-5.669674	4.505480	0.890868
67	6	0	-4.273375	4.446103	0.742678
68	1	0	-3.692174	5.356189	0.833131
69	1	0	-7.522289	3.406775	0.873736
70	1	0	-2.545652	3.234851	0.360395
71	7	0	-3.792865	-1.531069	-0.436535
72	6	0	-3.117366	-0.355285	-0.229171
73	1	0	-6.144572	5.456839	1.097333
74	6	0	-8.768937	-2.052732	-0.143995
75	8	0	-8.823649	-3.240447	-0.397520
76	8	0	-9.856863	-1.304414	0.098234
77	6	0	-11.129420	-1.982109	0.043131
78	1	0	-11.871388	-1.218329	0.259622
79	1	0	-11.289624	-2.402410	-0.949619
80	1	0	-11.166356	-2.775405	0.789620

19·Ba(ClO_4)$_2$ (S_0) CAM-B3LYP

Center Number	Atomic Number	Atomic Type	Coordinates (Angstroms) X	Y	Z
1	6	0	-0.514913	2.423466	-0.981545
2	6	0	-0.072595	3.097436	0.157194
3	6	0	0.364647	2.316321	-2.053738
4	6	0	1.247758	3.481773	0.282972
5	1	0	-0.740493	3.217630	1.000333
6	6	0	1.678257	2.738770	-1.950027
7	1	0	0.044062	1.823778	-2.963158
8	6	0	2.186842	3.208945	-0.729957
9	1	0	1.558467	3.917283	1.220027
10	1	0	2.320694	2.599396	-2.803167

11	6	0	3.911764	-3.220547	0.540582
12	1	0	4.321813	-3.129774	1.555073
13	1	0	4.540928	-3.929758	-0.012932
14	6	0	2.503456	-3.768948	0.546907
15	1	0	2.499618	-4.761367	1.010490
16	1	0	2.147853	-3.855194	-0.479064
17	6	0	1.368762	-3.111202	2.602348
18	1	0	1.582713	-4.148474	2.879633
19	1	0	0.314850	-2.905259	2.777026
20	6	0	2.210103	-2.169583	3.439542
21	1	0	3.275536	-2.433072	3.416600
22	1	0	1.865358	-2.224450	4.479857
23	6	0	2.651674	0.138744	3.731610
24	1	0	3.725012	-0.075102	3.831536
25	1	0	2.201036	0.151439	4.731990
26	6	0	2.427297	1.472645	3.058981
27	1	0	2.872143	2.271377	3.660393
28	1	0	1.355712	1.653933	2.972347
29	6	0	4.227647	2.121079	1.564317
30	1	0	4.860657	1.455019	0.982761
31	1	0	4.696847	2.299568	2.538802
32	6	0	4.084183	3.450943	0.826396
33	1	0	5.079796	3.898391	0.762177
34	1	0	3.483373	4.147968	1.411548
35	6	0	4.501302	3.092201	-1.596530
36	1	0	5.471891	3.439278	-1.239264
37	1	0	4.250670	3.718778	-2.460311
38	6	0	4.669341	1.664642	-2.123841
39	1	0	5.549498	1.678641	-2.783616
40	1	0	3.818346	1.329480	-2.719022
41	6	0	5.231778	-0.537517	-1.568128
42	1	0	4.535553	-0.858537	-2.346854
43	1	0	6.241791	-0.486512	-1.999163
44	6	0	5.243225	-1.556904	-0.461221
45	1	0	5.794900	-2.430452	-0.827758
46	1	0	5.769513	-1.166300	0.419648
47	7	0	3.551743	3.347242	-0.523165
48	8	0	2.973484	1.462952	1.743620
49	8	0	2.039139	-0.858352	2.935810
50	8	0	1.574219	-2.920383	1.207180
51	8	0	3.927401	-1.960296	-0.106684
52	8	0	4.871749	0.741005	-1.072142
53	56	0	1.148677	-0.371929	0.160269
54	6	0	-3.075624	1.906390	-0.738692
55	6	0	-2.774372	-0.312892	-0.928150
56	6	0	-5.007036	0.652412	-0.508149
57	6	0	-3.322160	-1.596275	-0.903357
58	1	0	-2.713519	-2.478747	-1.037953
59	6	0	-5.552117	-0.603960	-0.481084
60	1	0	-6.603485	-0.767646	-0.301437
61	6	0	-4.689580	-1.714721	-0.684551
62	6	0	-5.354855	2.060933	-0.351329
63	6	0	-4.161748	2.833062	-0.493543
64	6	0	-6.571877	2.684858	-0.101700
65	1	0	-7.478409	2.102081	0.009694
66	6	0	-4.215336	4.219646	-0.384689
67	6	0	-5.436627	4.820677	-0.137446
68	1	0	-5.491156	5.899284	-0.050784
69	6	0	-6.604447	4.063713	0.004039
70	1	0	-3.317615	4.815594	-0.492650
71	7	0	-3.668607	0.687638	-0.740380
72	1	0	-7.544367	4.565043	0.199276
73	7	0	-1.554455	0.246891	-1.058487

74	6	0	-1.729998	1.597891	-0.951002
75	6	0	-5.236700	-3.100690	-0.657321
76	8	0	-4.581317	-4.097113	-0.816532
77	8	0	-6.560496	-3.127243	-0.433975
78	6	0	-7.154241	-4.427559	-0.387867
79	1	0	-8.211723	-4.260495	-0.200661
80	1	0	-7.008336	-4.945517	-1.335529
81	1	0	-6.710806	-5.018701	0.413048
82	17	0	1.682071	-1.744427	-2.998127
83	17	0	-1.443983	-0.225610	2.537831
84	8	0	2.891479	-2.509953	-3.316973
85	8	0	2.074189	-0.369366	-2.514666
86	8	0	0.974379	-2.367154	-1.830710
87	8	0	0.794530	-1.639087	-4.145764
88	8	0	-1.010828	-1.397618	1.708481
89	8	0	-0.562797	0.911603	2.094961
90	8	0	-1.218566	-0.506998	3.956963
91	8	0	-2.841033	0.098399	2.265322

19·Ba(ClO$_4$)$_2$ (S$_1$) CAM-B3LYP

Center Number	Atomic Number	Atomic Type	Coordinates (Angstroms) X	Y	Z
1	6	0	-0.368426	2.475186	-1.322086
2	6	0	0.007083	3.208431	-0.187168
3	6	0	0.587005	2.292291	-2.327565
4	6	0	1.332887	3.551111	0.025808
5	1	0	-0.718324	3.387760	0.595399
6	6	0	1.905101	2.683499	-2.143247
7	1	0	0.317807	1.756979	-3.229236
8	6	0	2.338264	3.185391	-0.898411
9	1	0	1.589477	4.017927	0.964546
10	1	0	2.606258	2.494443	-2.939017
11	6	0	3.516740	-3.323247	0.977044
12	1	0	3.855573	-3.195775	2.013850
13	1	0	4.148334	-4.087376	0.504955
14	6	0	2.079447	-3.795503	0.902631
15	1	0	1.977693	-4.748327	1.434557
16	1	0	1.800208	-3.935651	-0.141146
17	6	0	0.866924	-2.880523	2.819296
18	1	0	0.993658	-3.898410	3.204028
19	1	0	-0.176652	-2.588473	2.909043
20	6	0	1.729843	-1.918875	3.620258
21	1	0	2.771885	-2.257306	3.697130
22	1	0	1.315194	-1.845492	4.633826
23	6	0	2.262081	0.397521	3.764418
24	1	0	3.304264	0.149590	4.012255
25	1	0	1.694057	0.518451	4.695660
26	6	0	2.187237	1.676462	2.955573
27	1	0	2.611583	2.505268	3.530733
28	1	0	1.145020	1.900331	2.729439
29	6	0	4.163797	2.134724	1.610213
30	1	0	4.819690	1.419633	1.119946
31	1	0	4.553646	2.350954	2.612403
32	6	0	4.122286	3.430723	0.793512
33	1	0	5.134644	3.846883	0.784747
34	1	0	3.496713	4.173981	1.288576
35	6	0	4.699953	2.821544	-1.545685
36	1	0	5.667803	3.084888	-1.115774
37	1	0	4.604288	3.401388	-2.471972

38	6	0	4.742387	1.342782	-1.956631
39	1	0	5.674091	1.208594	-2.526214
40	1	0	3.914408	1.054433	-2.605036
41	6	0	5.136778	-0.838204	-1.172796
42	1	0	4.525351	-1.217013	-1.994984
43	1	0	6.190471	-0.831098	-1.489065
44	6	0	5.008316	-1.756066	0.016156
45	1	0	5.563035	-2.672984	-0.215357
46	1	0	5.453091	-1.301133	0.911453
47	7	0	3.690326	3.266287	-0.589369
48	8	0	2.877575	1.512828	1.711205
49	8	0	1.694542	-0.647551	2.984131
50	8	0	1.153735	-2.842178	1.419565
51	8	0	3.643379	-2.092889	0.268697
52	8	0	4.745021	0.485576	-0.821853
53	56	0	1.157387	-0.358529	0.155087
54	6	0	-2.919030	1.922261	-1.028762
55	6	0	-2.579515	-0.308866	-1.209843
56	6	0	-4.803868	0.622642	-0.627794
57	6	0	-3.082611	-1.603549	-1.089246
58	1	0	-2.453006	-2.471005	-1.223522
59	6	0	-5.306034	-0.654156	-0.506281
60	1	0	-6.326929	-0.836403	-0.208671
61	6	0	-4.433012	-1.752252	-0.747529
62	6	0	-5.162811	2.020662	-0.426225
63	6	0	-3.991216	2.823834	-0.671977
64	6	0	-6.360100	2.623408	-0.044090
65	1	0	-7.242428	2.023611	0.146205
66	6	0	-4.054873	4.210533	-0.526707
67	6	0	-5.260045	4.787955	-0.145947
68	1	0	-5.321801	5.863753	-0.029545
69	6	0	-6.400991	4.005629	0.093637
70	1	0	-3.179479	4.822333	-0.708262
71	7	0	-3.491748	0.684811	-1.000587
72	1	0	-7.324281	4.487617	0.391897
73	7	0	-1.372540	0.278298	-1.402050
74	6	0	-1.569903	1.637069	-1.302241
75	6	0	-4.926981	-3.149285	-0.601598
76	8	0	-4.250614	-4.139999	-0.753656
77	8	0	-6.238199	-3.199459	-0.272753
78	6	0	-6.784196	-4.517769	-0.104180
79	1	0	-7.829933	-4.367763	0.154709
80	1	0	-6.693616	-5.088302	-1.029387
81	1	0	-6.261397	-5.046311	0.693955
82	17	0	1.732565	-1.977750	-2.852297
83	17	0	-1.632866	0.117351	2.289092
84	8	0	2.929309	-2.842321	-2.991287
85	8	0	2.176615	-0.574609	-2.444474
86	8	0	0.878311	-2.465667	-1.697289
87	8	0	0.952136	-1.922026	-4.094278
88	8	0	-1.240104	-1.134499	1.534580
89	8	0	-0.686211	1.197084	1.787130
90	8	0	-1.434920	-0.086913	3.743253
91	8	0	-3.023865	0.488427	1.974072

----

19 (S_0) M06

Center Number	Atomic Number	Atomic Type	Coordinates (Angstroms) X	Y	Z
1	6	0	-1.699911	-0.272689	-0.322824
2	6	0	-0.979479	0.778731	0.249547
3	6	0	-0.967506	-1.257946	-0.992075
4	6	0	0.393794	0.863294	0.145700
5	1	0	-1.504357	1.543512	0.815269
6	6	0	0.405504	-1.189363	-1.104018
7	1	0	-1.496549	-2.099133	-1.429536
8	6	0	1.135172	-0.117281	-0.546204
9	1	0	0.893229	1.713660	0.595499
10	1	0	0.919594	-1.994889	-1.615894
11	6	0	7.345356	-1.294471	2.626152
12	1	0	7.813471	-2.074926	2.008197
13	1	0	7.485488	-1.594663	3.677155
14	6	0	8.025288	0.030284	2.383872
15	1	0	9.073994	-0.030559	2.714819
16	1	0	7.532460	0.806529	2.979199
17	6	0	8.844377	-0.177896	0.157905
18	1	0	9.049681	-1.217855	0.457073
19	1	0	9.805828	0.360765	0.162580
20	6	0	8.259802	-0.206046	-1.229277
21	1	0	7.290955	-0.726089	-1.200827
22	1	0	8.932982	-0.778258	-1.883323
23	6	0	6.800421	1.430947	-2.168887
24	1	0	6.343512	0.603499	-2.736183
25	1	0	6.882939	2.287260	-2.846694
26	6	0	5.922117	1.774410	-0.986975
27	1	0	6.315034	2.662093	-0.463369
28	1	0	5.940797	0.940930	-0.272354
29	6	0	3.674765	2.156742	-0.413072
30	1	0	2.792129	2.638402	-0.848592
31	1	0	4.075098	2.824171	0.367908
32	6	0	3.286277	0.826702	0.210086
33	1	0	2.729887	1.012514	1.133660
34	1	0	4.183663	0.272066	0.510318
35	6	0	3.237362	-0.895321	-1.573046
36	1	0	4.049109	-0.310518	-2.021362
37	1	0	2.583627	-1.188401	-2.397230
38	6	0	3.841234	-2.123646	-0.921009
39	1	0	3.952380	-2.938308	-1.654914
40	1	0	3.177517	-2.485817	-0.118623
41	6	0	5.658220	-2.723413	0.483727
42	1	0	5.285423	-3.733558	0.257702
43	1	0	6.742549	-2.736886	0.311865
44	6	0	5.356819	-2.375227	1.925289
45	1	0	4.277004	-2.228754	2.050803
46	1	0	5.657991	-3.206975	2.581938
47	7	0	2.501842	-0.029839	-0.666902
48	8	0	4.615840	2.022236	-1.460438
49	8	0	8.107889	1.100572	-1.757973
50	8	0	7.937241	0.443192	1.039656
51	8	0	5.972998	-1.169816	2.325888
52	8	0	5.111471	-1.773445	-0.405759
53	6	0	-5.112481	-1.271282	-0.237229
54	6	0	-4.058234	0.689307	0.088834
55	7	0	-5.252560	0.048397	0.071388
56	6	0	-6.364723	0.802398	0.286740
57	6	0	-7.551829	0.109487	0.228482
58	6	0	-7.503656	-1.276638	-0.057502

59	6	0	-6.314413	-1.972928	-0.295552
60	1	0	-8.501953	0.602988	0.385977
61	1	0	-6.344234	-3.030609	-0.526886
62	6	0	-4.404551	2.068965	0.345466
63	6	0	-5.831887	2.133101	0.479262
64	6	0	-3.660148	3.245193	0.454461
65	6	0	-6.473506	3.342149	0.733278
66	6	0	-5.710182	4.488925	0.847557
67	6	0	-4.318224	4.436155	0.703269
68	1	0	-3.741813	5.352264	0.786838
69	1	0	-7.554257	3.379012	0.834841
70	1	0	-2.582089	3.238921	0.334536
71	7	0	-3.804976	-1.529554	-0.412113
72	6	0	-3.144758	-0.354660	-0.215288
73	1	0	-6.191618	5.441097	1.044878
74	6	0	-8.756806	-2.070686	-0.132931
75	8	0	-8.800135	-3.252880	-0.379881
76	8	0	-9.842184	-1.332224	0.103613
77	6	0	-11.095114	-2.015255	0.049593
78	1	0	-11.853871	-1.266059	0.266606
79	1	0	-11.256854	-2.440209	-0.943369
80	1	0	-11.128449	-2.813588	0.794055

19 (S_1) M06

Center Number	Atomic Number	Atomic Type	Coordinates (Angstroms) X	Y	Z
1	6	0	-1.670601	-0.277856	-0.340993
2	6	0	-0.937972	0.762678	0.250155
3	6	0	-0.943248	-1.257489	-1.037027
4	6	0	0.439523	0.840017	0.143243
5	1	0	-1.451792	1.520082	0.827902
6	6	0	0.433292	-1.194834	-1.154329
7	1	0	-1.477029	-2.083558	-1.489820
8	6	0	1.176377	-0.135975	-0.572649
9	1	0	0.942780	1.675535	0.607648
10	1	0	0.937314	-1.988619	-1.686491
11	6	0	7.227887	-1.300849	2.702754
12	1	0	7.721928	-2.069992	2.100645
13	1	0	7.357031	-1.578490	3.757965
14	6	0	7.875639	0.051127	2.458460
15	1	0	8.909963	0.028975	2.826035
16	1	0	7.334059	0.819826	3.014030
17	6	0	8.793775	-0.162827	0.246055
18	1	0	8.993936	-1.195776	0.555515
19	1	0	9.739987	0.392025	0.297126
20	6	0	8.275218	-0.200494	-1.177424
21	1	0	7.304038	-0.703828	-1.195298
22	1	0	8.977780	-0.779998	-1.787260
23	6	0	6.866120	1.466022	-2.197967
24	1	0	6.410589	0.637572	-2.754252
25	1	0	6.995396	2.304474	-2.885121
26	6	0	5.965776	1.854537	-1.033628
27	1	0	6.331217	2.774382	-0.557922
28	1	0	5.992188	1.061285	-0.285050
29	6	0	3.678069	2.170219	-0.482793
30	1	0	2.788846	2.609185	-0.938583
31	1	0	4.047032	2.858890	0.289570
32	6	0	3.335068	0.824689	0.159894
33	1	0	2.799358	1.000571	1.093530

34	1	0	4.247953	0.293366	0.430989
35	6	0	3.273993	-0.912440	-1.625646
36	1	0	4.087908	-0.324547	-2.052337
37	1	0	2.617092	-1.179318	-2.452377
38	6	0	3.862865	-2.174272	-1.005143
39	1	0	4.032879	-2.933250	-1.780820
40	1	0	3.169388	-2.589218	-0.264567
41	6	0	5.609808	-2.799889	0.518173
42	1	0	5.222865	-3.798008	0.281000
43	1	0	6.693366	-2.830840	0.381214
44	6	0	5.260105	-2.450928	1.959254
45	1	0	4.180094	-2.320861	2.057193
46	1	0	5.564437	-3.273822	2.620168
47	7	0	2.544214	-0.060686	-0.691740
48	8	0	4.643620	2.055227	-1.529427
49	8	0	8.169879	1.110604	-1.745822
50	8	0	7.828351	0.449989	1.093594
51	8	0	5.841749	-1.221082	2.384315
52	8	0	5.107398	-1.829591	-0.396154
53	6	0	-5.108171	-1.271110	-0.254755
54	6	0	-4.034806	0.691823	0.090325
55	7	0	-5.236493	0.053212	0.069342
56	6	0	-6.351043	0.811353	0.295644
57	6	0	-7.544926	0.120539	0.233453
58	6	0	-7.506431	-1.268067	-0.066857
59	6	0	-6.314110	-1.969810	-0.316753
60	1	0	-8.489879	0.614340	0.398206
61	1	0	-6.345826	-3.022214	-0.558109
62	6	0	-4.374543	2.074665	0.361797
63	6	0	-5.812009	2.144721	0.500818
64	6	0	-3.620418	3.248193	0.480657
65	6	0	-6.444918	3.359937	0.768407
66	6	0	-5.669674	4.505480	0.890868
67	6	0	-4.273375	4.446103	0.742678
68	1	0	-3.692174	5.356189	0.833131
69	1	0	-7.522289	3.406775	0.873736
70	1	0	-2.545652	3.234851	0.360395
71	7	0	-3.792865	-1.531069	-0.436535
72	6	0	-3.117366	-0.355285	-0.229171
73	1	0	-6.144572	5.456839	1.097333
74	6	0	-8.768937	-2.052732	-0.143995
75	8	0	-8.823649	-3.240447	-0.397520
76	8	0	-9.856863	-1.304414	0.098234
77	6	0	-11.129420	-1.982109	0.043131
78	1	0	-11.871388	-1.218329	0.259622
79	1	0	-11.289624	-2.402410	-0.949619
80	1	0	-11.166356	-2.775405	0.789620

19·Ba(ClO4)2 (S0) M06

Center Number	Atomic Number	Atomic Type	Coordinates (Angstroms) X	Y	Z
1	6	0	-0.335517	2.279349	-1.393611
2	6	0	0.034963	3.120145	-0.341491
3	6	0	0.624813	1.979976	-2.356679
4	6	0	1.354337	3.484329	-0.160358
5	1	0	-0.701310	3.387735	0.411604
6	6	0	1.937086	2.386106	-2.207997
7	1	0	0.357208	1.350514	-3.200798
8	6	0	2.361344	3.030498	-1.034648

9	1	0	1.605722	4.059394	0.722689
10	1	0	2.642484	2.107244	-2.977092
11	6	0	3.524380	-3.220287	1.156532
12	1	0	3.847601	-3.010687	2.190926
13	1	0	4.176595	-4.015400	0.760033
14	6	0	2.103275	-3.708100	1.095314
15	1	0	2.001898	-4.631855	1.682672
16	1	0	1.846554	-3.924629	0.054016
17	6	0	0.873056	-2.702641	2.919999
18	1	0	1.005408	-3.698013	3.367031
19	1	0	-0.180600	-2.424788	2.989779
20	6	0	1.707977	-1.693313	3.674669
21	1	0	2.752509	-2.024233	3.806622
22	1	0	1.268450	-1.558257	4.676119
23	6	0	2.239544	0.600706	3.692468
24	1	0	3.269776	0.351801	4.005093
25	1	0	1.636091	0.795533	4.593624
26	6	0	2.236938	1.813006	2.800048
27	1	0	2.652416	2.676257	3.337441
28	1	0	1.205260	2.050215	2.516419
29	6	0	4.230498	2.198676	1.499090
30	1	0	4.927016	1.483057	1.056635
31	1	0	4.607251	2.481220	2.494522
32	6	0	4.143555	3.435487	0.616583
33	1	0	5.139744	3.894285	0.575749
34	1	0	3.493945	4.186808	1.076398
35	6	0	4.722526	2.713379	-1.679678
36	1	0	5.683450	3.041498	-1.269220
37	1	0	4.603979	3.242425	-2.637281
38	6	0	4.829151	1.224462	-2.005013
39	1	0	5.812729	1.080158	-2.486547
40	1	0	4.073193	0.878543	-2.721811
41	6	0	5.162587	-0.886795	-1.092239
42	1	0	4.583242	-1.329485	-1.914847
43	1	0	6.228529	-0.886397	-1.378483
44	6	0	5.003323	-1.719783	0.141961
45	1	0	5.588364	-2.641256	0.000399
46	1	0	5.410730	-1.193278	1.022378
47	7	0	3.708329	3.165457	-0.742327
48	8	0	2.980867	1.539108	1.621443
49	8	0	1.690672	-0.474652	2.966698
50	8	0	1.174303	-2.738702	1.536321
51	8	0	3.649266	-2.059810	0.362330
52	8	0	4.744847	0.437749	-0.842431
53	56	0	1.142369	-0.323512	0.121783
54	6	0	-2.882734	1.856113	-1.035527
55	6	0	-2.655190	-0.377158	-1.186994
56	6	0	-4.820084	0.671089	-0.603453
57	6	0	-3.227206	-1.639303	-1.047031
58	1	0	-2.647481	-2.545266	-1.179532
59	6	0	-5.388065	-0.570858	-0.467092
60	1	0	-6.421215	-0.695136	-0.170054
61	6	0	-4.575444	-1.710378	-0.697620
62	6	0	-5.104503	2.083870	-0.437278
63	6	0	-3.904296	2.816244	-0.703395
64	6	0	-6.270317	2.750392	-0.078579
65	1	0	-7.180668	2.194444	0.127038
66	6	0	-3.903095	4.204885	-0.606302
67	6	0	-5.075246	4.847375	-0.249691
68	1	0	-5.087735	5.930310	-0.171559
69	6	0	-6.247701	4.130476	0.013090
70	1	0	-2.999045	4.770640	-0.811217
71	7	0	-3.511800	0.655734	-0.973552

72	1	0	-7.149119	4.666390	0.292669
73	7	0	-1.431285	0.137544	-1.420329
74	6	0	-1.562955	1.494681	-1.329902
75	6	0	-5.140789	-3.076428	-0.548840
76	8	0	-4.522746	-4.095734	-0.706192
77	8	0	-6.442444	-3.053384	-0.215508
78	6	0	-7.051579	-4.328087	-0.051504
79	1	0	-8.089804	-4.134101	0.214905
80	1	0	-6.998182	-4.904207	-0.978776
81	1	0	-6.555334	-4.894129	0.740826
82	17	0	1.769836	-2.178021	-2.706991
83	17	0	-1.620877	0.178206	2.265554
84	8	0	2.909354	-3.093968	-2.733847
85	8	0	2.261634	-0.785368	-2.412824
86	8	0	0.872304	-2.523691	-1.561382
87	8	0	1.035481	-2.190065	-3.956277
88	8	0	-1.255522	-1.055270	1.508590
89	8	0	-0.684404	1.240282	1.769832
90	8	0	-1.410701	-0.034282	3.697076
91	8	0	-2.995306	0.557327	1.972504

19·Ba(ClO$_4$)$_2$ (S$_1$) M06

Center Number	Atomic Number	Atomic Type	Coordinates (Angstroms) X	Y	Z
1	6	0	-0.368426	2.475186	-1.322086
2	6	0	0.007083	3.208431	-0.187168
3	6	0	0.587005	2.292291	-2.327565
4	6	0	1.332887	3.551111	0.025808
5	1	0	-0.718324	3.387760	0.595399
6	6	0	1.905101	2.683499	-2.143247
7	1	0	0.317807	1.756979	-3.229236
8	6	0	2.338264	3.185391	-0.898411
9	1	0	1.589477	4.017927	0.964546
10	1	0	2.606258	2.494443	-2.939017
11	6	0	3.516740	-3.323247	0.977044
12	1	0	3.855573	-3.195775	2.013850
13	1	0	4.148334	-4.087376	0.504955
14	6	0	2.079447	-3.795503	0.902631
15	1	0	1.977693	-4.748327	1.434557
16	1	0	1.800208	-3.935651	-0.141146
17	6	0	0.866924	-2.880523	2.819296
18	1	0	0.993658	-3.898410	3.204028
19	1	0	-0.176652	-2.588473	2.909043
20	6	0	1.729843	-1.918875	3.620258
21	1	0	2.771885	-2.257306	3.697130
22	1	0	1.315194	-1.845492	4.633826
23	6	0	2.262081	0.397521	3.764418
24	1	0	3.304264	0.149590	4.012255
25	1	0	1.694057	0.518451	4.695660
26	6	0	2.187237	1.676462	2.955573
27	1	0	2.611583	2.505268	3.530733
28	1	0	1.145020	1.900331	2.729439
29	6	0	4.163797	2.134724	1.610213
30	1	0	4.819690	1.419633	1.119946
31	1	0	4.553646	2.350954	2.612403
32	6	0	4.122286	3.430723	0.793512
33	1	0	5.134644	3.846883	0.784747
34	1	0	3.496713	4.173981	1.288576
35	6	0	4.699953	2.821544	-1.545685

36	1	0	5.667803	3.084888	-1.115774
37	1	0	4.604288	3.401388	-2.471972
38	6	0	4.742387	1.342782	-1.956631
39	1	0	5.674091	1.208594	-2.526214
40	1	0	3.914408	1.054433	-2.605036
41	6	0	5.136778	-0.838204	-1.172796
42	1	0	4.525351	-1.217013	-1.994984
43	1	0	6.190471	-0.831098	-1.489065
44	6	0	5.008316	-1.756066	0.016156
45	1	0	5.563035	-2.672984	-0.215357
46	1	0	5.453091	-1.301133	0.911453
47	7	0	3.690326	3.266287	-0.589369
48	8	0	2.877575	1.512828	1.711205
49	8	0	1.694542	-0.647551	2.984131
50	8	0	1.153735	-2.842178	1.419565
51	8	0	3.643379	-2.092889	0.268697
52	8	0	4.745021	0.485576	-0.821853
53	56	0	1.157387	-0.358529	0.155087
54	6	0	-2.919030	1.922261	-1.028762
55	6	0	-2.579515	-0.308866	-1.209843
56	6	0	-4.803868	0.622642	-0.627794
57	6	0	-3.082611	-1.603549	-1.089246
58	1	0	-2.453006	-2.471005	-1.223522
59	6	0	-5.306034	-0.654156	-0.506281
60	1	0	-6.326929	-0.836403	-0.208671
61	6	0	-4.433012	-1.752252	-0.747529
62	6	0	-5.162811	2.020662	-0.426225
63	6	0	-3.991216	2.823834	-0.671977
64	6	0	-6.360100	2.623408	-0.044090
65	1	0	-7.242428	2.023611	0.146205
66	6	0	-4.054873	4.210533	-0.526707
67	6	0	-5.260045	4.787955	-0.145947
68	1	0	-5.321801	5.863753	-0.029545
69	6	0	-6.400991	4.005629	0.093637
70	1	0	-3.179479	4.822333	-0.708262
71	7	0	-3.491748	0.684811	-1.000587
72	1	0	-7.324281	4.487617	0.391897
73	7	0	-1.372540	0.278298	-1.402050
74	6	0	-1.569903	1.637069	-1.302241
75	6	0	-4.926981	-3.149285	-0.601598
76	8	0	-4.250614	-4.139999	-0.753656
77	8	0	-6.238199	-3.199459	-0.272753
78	6	0	-6.784196	-4.517769	-0.104180
79	1	0	-7.829933	-4.367763	0.154709
80	1	0	-6.693616	-5.088302	-1.029387
81	1	0	-6.261397	-5.046311	0.693955
82	17	0	1.732565	-1.977750	-2.852297
83	17	0	-1.632866	0.117351	2.289092
84	8	0	2.929309	-2.842321	-2.991287
85	8	0	2.176615	-0.574609	-2.444474
86	8	0	0.878311	-2.465667	-1.697289
87	8	0	0.952136	-1.922026	-4.094278
88	8	0	-1.240104	-1.134499	1.534580
89	8	0	-0.686211	1.197084	1.787130
90	8	0	-1.434920	-0.086913	3.743253
91	8	0	-3.023865	0.488427	1.974072

----

19 (S_0) M06-L

Center Number	Atomic Number	Atomic Type	Coordinates (Angstroms) X	Y	Z
1	6	0	-1.689697	-0.175691	-0.401018
2	6	0	-0.978576	0.902888	0.145232
3	6	0	-0.939161	-1.170575	-1.047673
4	6	0	0.395644	0.998360	0.047202
5	1	0	-1.512790	1.678856	0.683215
6	6	0	0.434502	-1.090136	-1.157298
7	1	0	-1.458323	-2.027339	-1.463949
8	6	0	1.152513	0.003321	-0.615143
9	1	0	0.885477	1.864621	0.474157
10	1	0	0.960158	-1.899528	-1.648669
11	6	0	7.272874	-1.392469	2.732408
12	1	0	7.724522	-2.195531	2.135276
13	1	0	7.439741	-1.651675	3.788546
14	6	0	7.938213	-0.072576	2.418502
15	1	0	8.991838	-0.105177	2.732252
16	1	0	7.449168	0.728088	2.981212
17	6	0	8.746885	-0.362999	0.197475
18	1	0	8.861346	-1.426588	0.457342
19	1	0	9.740028	0.103148	0.294311
20	6	0	8.248673	-0.278291	-1.222688
21	1	0	7.252561	-0.733761	-1.285326
22	1	0	8.924586	-0.852231	-1.870328
23	6	0	6.939421	1.492501	-2.150164
24	1	0	6.508167	0.752476	-2.841982
25	1	0	7.107039	2.412794	-2.716505
26	6	0	5.986574	1.730998	-0.997638
27	1	0	6.364504	2.533657	-0.345220
28	1	0	5.928139	0.819786	-0.389457
29	6	0	3.746246	2.269432	-0.502157
30	1	0	2.898453	2.770399	-0.978283
31	1	0	4.145474	2.940813	0.274271
32	6	0	3.294651	0.969352	0.147972
33	1	0	2.709539	1.200526	1.040479
34	1	0	4.162564	0.401715	0.499480
35	6	0	3.270231	-0.809778	-1.580221
36	1	0	4.110622	-0.253250	-2.006006
37	1	0	2.641183	-1.103756	-2.421072
38	6	0	3.822767	-2.039750	-0.879910
39	1	0	3.924880	-2.876365	-1.588511
40	1	0	3.139622	-2.366389	-0.081002
41	6	0	5.641023	-2.707154	0.495285
42	1	0	5.297983	-3.704236	0.185808
43	1	0	6.726931	-2.683649	0.349785
44	6	0	5.298114	-2.472521	1.953040
45	1	0	4.216339	-2.349505	2.068938
46	1	0	5.595831	-3.347736	2.549555
47	7	0	2.519334	0.098743	-0.726968
48	8	0	4.716113	2.084458	-1.524271
49	8	0	8.209775	1.071343	-1.683803
50	8	0	7.820529	0.281831	1.051745
51	8	0	5.881873	-1.285529	2.466542
52	8	0	5.099801	-1.705405	-0.352962
53	6	0	-5.080745	-1.246925	-0.318906
54	6	0	-4.067688	0.732636	0.066723
55	7	0	-5.246855	0.062001	0.038249
56	6	0	-6.377066	0.777188	0.306686
57	6	0	-7.550090	0.049974	0.244955
58	6	0	-7.474256	-1.323739	-0.094649

59	6	0	-6.264430	-1.978794	-0.381931
60	1	0	-8.508451	0.509686	0.442117
61	1	0	-6.272132	-3.027202	-0.649838
62	6	0	-4.444194	2.089473	0.386713
63	6	0	-5.877030	2.110349	0.546429
64	6	0	-3.729932	3.283891	0.533343
65	6	0	-6.544354	3.293948	0.862571
66	6	0	-5.807872	4.458031	1.012997
67	6	0	-4.415518	4.448625	0.843476
68	1	0	-3.861288	5.374684	0.955094
69	1	0	-7.622938	3.299216	0.983033
70	1	0	-2.655425	3.313761	0.395710
71	7	0	-3.766689	-1.470088	-0.521090
72	6	0	-3.128385	-0.282670	-0.287075
73	1	0	-6.310967	5.387061	1.257693
74	6	0	-8.702988	-2.147535	-0.173768
75	8	0	-8.726365	-3.326048	-0.467987
76	8	0	-9.809696	-1.442465	0.120214
77	6	0	-11.038845	-2.176971	0.064130
78	1	0	-11.818924	-1.469154	0.332061
79	1	0	-11.206998	-2.564831	-0.941506
80	1	0	-11.020263	-3.007818	0.770749

19 (S_1) M06-L

Center Number	Atomic Number	Atomic Type	Coordinates (Angstroms) X	Y	Z
1	6	0	-1.670601	-0.277856	-0.340993
2	6	0	-0.937972	0.762678	0.250155
3	6	0	-0.943248	-1.257489	-1.037027
4	6	0	0.439523	0.840017	0.143243
5	1	0	-1.451792	1.520082	0.827902
6	6	0	0.433292	-1.194834	-1.154329
7	1	0	-1.477029	-2.083558	-1.489820
8	6	0	1.176377	-0.135975	-0.572649
9	1	0	0.942780	1.675535	0.607648
10	1	0	0.937314	-1.988619	-1.686491
11	6	0	7.227887	-1.300849	2.702754
12	1	0	7.721928	-2.069992	2.100645
13	1	0	7.357031	-1.578490	3.757965
14	6	0	7.875639	0.051127	2.458460
15	1	0	8.909963	0.028975	2.826035
16	1	0	7.334059	0.819826	3.014030
17	6	0	8.793775	-0.162827	0.246055
18	1	0	8.993936	-1.195776	0.555515
19	1	0	9.739987	0.392025	0.297126
20	6	0	8.275218	-0.200494	-1.177424
21	1	0	7.304038	-0.703828	-1.195298
22	1	0	8.977780	-0.779998	-1.787260
23	6	0	6.866120	1.466022	-2.197967
24	1	0	6.410589	0.637572	-2.754252
25	1	0	6.995396	2.304474	-2.885121
26	6	0	5.965776	1.854537	-1.033628
27	1	0	6.331217	2.774382	-0.557922
28	1	0	5.992188	1.061285	-0.285050
29	6	0	3.678069	2.170219	-0.482793
30	1	0	2.788846	2.609185	-0.938583
31	1	0	4.047032	2.858890	0.289570
32	6	0	3.335068	0.824689	0.159894

33	1	0	2.799358	1.000571	1.093530
34	1	0	4.247953	0.293366	0.430989
35	6	0	3.273993	-0.912440	-1.625646
36	1	0	4.087908	-0.324547	-2.052337
37	1	0	2.617092	-1.179318	-2.452377
38	6	0	3.862865	-2.174272	-1.005143
39	1	0	4.032879	-2.933250	-1.780820
40	1	0	3.169388	-2.589218	-0.264567
41	6	0	5.609808	-2.799889	0.518173
42	1	0	5.222865	-3.798008	0.281000
43	1	0	6.693366	-2.830840	0.381214
44	6	0	5.260105	-2.450928	1.959254
45	1	0	4.180094	-2.320861	2.057193
46	1	0	5.564437	-3.273822	2.620168
47	7	0	2.544214	-0.060686	-0.691740
48	8	0	4.643620	2.055227	-1.529427
49	8	0	8.169879	1.110604	-1.745822
50	8	0	7.828351	0.449989	1.093594
51	8	0	5.841749	-1.221082	2.384315
52	8	0	5.107398	-1.829591	-0.396154
53	6	0	-5.108171	-1.271110	-0.254755
54	6	0	-4.034806	0.691823	0.090325
55	7	0	-5.236493	0.053212	0.069342
56	6	0	-6.351043	0.811353	0.295644
57	6	0	-7.544926	0.120539	0.233453
58	6	0	-7.506431	-1.268067	-0.066857
59	6	0	-6.314110	-1.969810	-0.316753
60	1	0	-8.489879	0.614340	0.398206
61	1	0	-6.345826	-3.022214	-0.558109
62	6	0	-4.374543	2.074665	0.361797
63	6	0	-5.812009	2.144721	0.500818
64	6	0	-3.620418	3.248193	0.480657
65	6	0	-6.444918	3.359937	0.768407
66	6	0	-5.669674	4.505480	0.890868
67	6	0	-4.273375	4.446103	0.742678
68	1	0	-3.692174	5.356189	0.833131
69	1	0	-7.522289	3.406775	0.873736
70	1	0	-2.545652	3.234851	0.360395
71	7	0	-3.792865	-1.531069	-0.436535
72	6	0	-3.117366	-0.355285	-0.229171
73	1	0	-6.144572	5.456839	1.097333
74	6	0	-8.768937	-2.052732	-0.143995
75	8	0	-8.823649	-3.240447	-0.397520
76	8	0	-9.856863	-1.304414	0.098234
77	6	0	-11.129420	-1.982109	0.043131
78	1	0	-11.871388	-1.218329	0.259622
79	1	0	-11.289624	-2.402410	-0.949619
80	1	0	-11.166356	-2.775405	0.789620

19·Ba(ClO$_4$)$_2$ (S_0) M06-L

Center Number	Atomic Number	Atomic Type	Coordinates (Angstroms) X	Y	Z
1	6	0	-0.322763	2.219036	-1.449290
2	6	0	0.072646	3.088244	-0.423256
3	6	0	0.641452	1.845954	-2.391434
4	6	0	1.404087	3.416090	-0.246613
5	1	0	-0.653967	3.408510	0.315969
6	6	0	1.967958	2.217916	-2.253312
7	1	0	0.361918	1.184340	-3.205231
8	6	0	2.404717	2.916004	-1.110693

9	1	0	1.669647	4.018278	0.612536
10	1	0	2.671018	1.879804	-2.999621
11	6	0	3.391046	-3.225384	1.260765
12	1	0	3.721795	-3.028586	2.293592
13	1	0	4.032093	-4.023567	0.856293
14	6	0	1.958753	-3.684322	1.201977
15	1	0	1.834983	-4.586535	1.815552
16	1	0	1.693825	-3.921277	0.169969
17	6	0	0.771989	-2.592412	3.006679
18	1	0	0.906199	-3.573059	3.481343
19	1	0	-0.275759	-2.303111	3.086673
20	6	0	1.629646	-1.563570	3.710458
21	1	0	2.668061	-1.900728	3.860328
22	1	0	1.195808	-1.364399	4.701464
23	6	0	2.161217	0.732870	3.644048
24	1	0	3.152247	0.474954	4.052762
25	1	0	1.494915	0.991286	4.480460
26	6	0	2.264724	1.891670	2.686723
27	1	0	2.669609	2.769616	3.203838
28	1	0	1.267993	2.146881	2.311197
29	6	0	4.311110	2.242868	1.446269
30	1	0	5.044934	1.534297	1.061460
31	1	0	4.649700	2.593241	2.431954
32	6	0	4.200062	3.428879	0.492630
33	1	0	5.189586	3.892838	0.412480
34	1	0	3.547878	4.199505	0.910636
35	6	0	4.760174	2.603619	-1.780574
36	1	0	5.715322	2.981963	-1.406944
37	1	0	4.603796	3.078362	-2.758743
38	6	0	4.889007	1.096470	-2.020720
39	1	0	5.898525	0.916589	-2.426379
40	1	0	4.180058	0.713639	-2.764023
41	6	0	5.151710	-0.962306	-0.963983
42	1	0	4.628610	-1.448288	-1.796123
43	1	0	6.232560	-0.972774	-1.181441
44	6	0	4.915055	-1.727780	0.303179
45	1	0	5.495561	-2.658444	0.241636
46	1	0	5.277769	-1.166169	1.179197
47	7	0	3.752260	3.080275	-0.845224
48	8	0	3.089598	1.522560	1.578222
49	8	0	1.636175	-0.375715	2.934794
50	8	0	1.042331	-2.678900	1.611211
51	8	0	3.539843	-2.052692	0.471413
52	8	0	4.718551	0.380772	-0.815039
53	56	0	1.151157	-0.269497	0.108547
54	6	0	-2.886832	1.841881	-1.067778
55	6	0	-2.682078	-0.401246	-1.211066
56	6	0	-4.839424	0.675540	-0.631271
57	6	0	-3.263203	-1.657011	-1.061374
58	1	0	-2.692136	-2.568447	-1.184935
59	6	0	-5.418854	-0.567056	-0.487429
60	1	0	-6.451582	-0.680469	-0.188142
61	6	0	-4.618436	-1.717689	-0.709374
62	6	0	-5.110144	2.089033	-0.469192
63	6	0	-3.897245	2.812077	-0.739267
64	6	0	-6.269770	2.771493	-0.108317
65	1	0	-7.185532	2.227239	0.100340
66	6	0	-3.883843	4.203852	-0.642571
67	6	0	-5.051380	4.860560	-0.283537
68	1	0	-5.051888	5.942987	-0.206007
69	6	0	-6.232604	4.154708	-0.017445
70	1	0	-2.976160	4.761822	-0.848569
71	7	0	-3.528785	0.646861	-1.004398

72	1	0	-7.127623	4.699173	0.263682
73	7	0	-1.448031	0.099567	-1.449080
74	6	0	-1.565372	1.462378	-1.364620
75	6	0	-5.191527	-3.075491	-0.547050
76	8	0	-4.582020	-4.108549	-0.696845
77	8	0	-6.501109	-3.036577	-0.205656
78	6	0	-7.106077	-4.317955	-0.028867
79	1	0	-8.142493	-4.123783	0.238927
80	1	0	-7.051885	-4.900697	-0.950386
81	1	0	-6.604467	-4.874787	0.764837
82	17	0	1.864214	-2.279457	-2.600631
83	17	0	-1.606570	0.264658	2.239570
84	8	0	3.008534	-3.195352	-2.504701
85	8	0	2.340712	-0.860006	-2.374497
86	8	0	0.900220	-2.552723	-1.480754
87	8	0	1.196683	-2.378619	-3.889311
88	8	0	-1.236571	-0.990210	1.508206
89	8	0	-0.698825	1.334622	1.683196
90	8	0	-1.354409	0.098349	3.676924
91	8	0	-2.999531	0.613386	1.972534

19·Ba(ClO$_4$)$_2$ (S_1) M06-L

Center Number	Atomic Number	Atomic Type	Coordinates (Angstroms) X	Y	Z
1	6	0	-0.368426	2.475186	-1.322086
2	6	0	0.007083	3.208431	-0.187168
3	6	0	0.587005	2.292291	-2.327565
4	6	0	1.332887	3.551111	0.025808
5	1	0	-0.718324	3.387760	0.595399
6	6	0	1.905101	2.683499	-2.143247
7	1	0	0.317807	1.756979	-3.229236
8	6	0	2.338264	3.185391	-0.898411
9	1	0	1.589477	4.017927	0.964546
10	1	0	2.606258	2.494443	-2.939017
11	6	0	3.516740	-3.323247	0.977044
12	1	0	3.855573	-3.195775	2.013850
13	1	0	4.148334	-4.087376	0.504955
14	6	0	2.079447	-3.795503	0.902631
15	1	0	1.977693	-4.748327	1.434557
16	1	0	1.800208	-3.935651	-0.141146
17	6	0	0.866924	-2.880523	2.819296
18	1	0	0.993658	-3.898410	3.204028
19	1	0	-0.176652	-2.588473	2.909043
20	6	0	1.729843	-1.918875	3.620258
21	1	0	2.771885	-2.257306	3.697130
22	1	0	1.315194	-1.845492	4.633826
23	6	0	2.262081	0.397521	3.764418
24	1	0	3.304264	0.149590	4.012255
25	1	0	1.694057	0.518451	4.695660
26	6	0	2.187237	1.676462	2.955573
27	1	0	2.611583	2.505268	3.530733
28	1	0	1.145020	1.900331	2.729439
29	6	0	4.163797	2.134724	1.610213
30	1	0	4.819690	1.419633	1.119946
31	1	0	4.553646	2.350954	2.612403
32	6	0	4.122286	3.430723	0.793512
33	1	0	5.134644	3.846883	0.784747
34	1	0	3.496713	4.173981	1.288576
35	6	0	4.699953	2.821544	-1.545685

36	1	0	5.667803	3.084888	-1.115774
37	1	0	4.604288	3.401388	-2.471972
38	6	0	4.742387	1.342782	-1.956631
39	1	0	5.674091	1.208594	-2.526214
40	1	0	3.914408	1.054433	-2.605036
41	6	0	5.136778	-0.838204	-1.172796
42	1	0	4.525351	-1.217013	-1.994984
43	1	0	6.190471	-0.831098	-1.489065
44	6	0	5.008316	-1.756066	0.016156
45	1	0	5.563035	-2.672984	-0.215357
46	1	0	5.453091	-1.301133	0.911453
47	7	0	3.690326	3.266287	-0.589369
48	8	0	2.877575	1.512828	1.711205
49	8	0	1.694542	-0.647551	2.984131
50	8	0	1.153735	-2.842178	1.419565
51	8	0	3.643379	-2.092889	0.268697
52	8	0	4.745021	0.485576	-0.821853
53	56	0	1.157387	-0.358529	0.155087
54	6	0	-2.919030	1.922261	-1.028762
55	6	0	-2.579515	-0.308866	-1.209843
56	6	0	-4.803868	0.622642	-0.627794
57	6	0	-3.082611	-1.603549	-1.089246
58	1	0	-2.453006	-2.471005	-1.223522
59	6	0	-5.306034	-0.654156	-0.506281
60	1	0	-6.326929	-0.836403	-0.208671
61	6	0	-4.433012	-1.752252	-0.747529
62	6	0	-5.162811	2.020662	-0.426225
63	6	0	-3.991216	2.823834	-0.671977
64	6	0	-6.360100	2.623408	-0.044090
65	1	0	-7.242428	2.023611	0.146205
66	6	0	-4.054873	4.210533	-0.526707
67	6	0	-5.260045	4.787955	-0.145947
68	1	0	-5.321801	5.863753	-0.029545
69	6	0	-6.400991	4.005629	0.093637
70	1	0	-3.179479	4.822333	-0.708262
71	7	0	-3.491748	0.684811	-1.000587
72	1	0	-7.324281	4.487617	0.391897
73	7	0	-1.372540	0.278298	-1.402050
74	6	0	-1.569903	1.637069	-1.302241
75	6	0	-4.926981	-3.149285	-0.601598
76	8	0	-4.250614	-4.139999	-0.753656
77	8	0	-6.238199	-3.199459	-0.272753
78	6	0	-6.784196	-4.517769	-0.104180
79	1	0	-7.829933	-4.367763	0.154709
80	1	0	-6.693616	-5.088302	-1.029387
81	1	0	-6.261397	-5.046311	0.693955
82	17	0	1.732565	-1.977750	-2.852297
83	17	0	-1.632866	0.117351	2.289092
84	8	0	2.929309	-2.842321	-2.991287
85	8	0	2.176615	-0.574609	-2.444474
86	8	0	0.878311	-2.465667	-1.697289
87	8	0	0.952136	-1.922026	-4.094278
88	8	0	-1.240104	-1.134499	1.534580
89	8	0	-0.686211	1.197084	1.787130
90	8	0	-1.434920	-0.086913	3.743253
91	8	0	-3.023865	0.488427	1.974072

----

19 (S_0) M06-2X

Center Number	Atomic Number	Atomic Type	Coordinates (Angstroms) X	Y	Z
1	6	0	-1.695557	-0.185657	-0.298252
2	6	0	-1.018040	0.825519	0.390458
3	6	0	-0.932704	-1.074418	-1.062254
4	6	0	0.356052	0.963864	0.308923
5	1	0	-1.574311	1.507586	1.024226
6	6	0	0.442686	-0.951691	-1.153028
7	1	0	-1.432242	-1.878538	-1.589833
8	6	0	1.132009	0.079977	-0.474822
9	1	0	0.826545	1.778945	0.842289
10	1	0	0.985682	-1.680718	-1.739124
11	6	0	7.470722	-1.861114	2.434299
12	1	0	7.928644	-2.546467	1.713561
13	1	0	7.708303	-2.232906	3.437976
14	6	0	8.037679	-0.463646	2.266604
15	1	0	9.110123	-0.466785	2.499091
16	1	0	7.536943	0.211169	2.964180
17	6	0	8.750014	-0.337775	-0.001223
18	1	0	8.941320	-1.418540	0.035744
19	1	0	9.702477	0.180439	0.171752
20	6	0	8.203489	0.006865	-1.369497
21	1	0	7.221244	-0.463517	-1.489021
22	1	0	8.879439	-0.390009	-2.133718
23	6	0	6.811868	1.895297	-1.848970
24	1	0	6.363557	1.304865	-2.658628
25	1	0	6.933923	2.920430	-2.202734
26	6	0	5.906935	1.861005	-0.629190
27	1	0	6.323567	2.493654	0.165303
28	1	0	5.849485	0.835685	-0.253608
29	6	0	3.692918	2.334751	0.044702
30	1	0	2.830093	2.903963	-0.307228
31	1	0	4.119104	2.847177	0.918080
32	6	0	3.257557	0.929640	0.453233
33	1	0	2.663115	0.995762	1.365315
34	1	0	4.130676	0.321477	0.702658
35	6	0	3.258655	-0.499437	-1.578458
36	1	0	4.080243	0.141040	-1.908187
37	1	0	2.623653	-0.665385	-2.447912
38	6	0	3.862324	-1.818316	-1.106027
39	1	0	3.993695	-2.499447	-1.956751
40	1	0	3.202353	-2.302356	-0.374362
41	6	0	5.797405	-2.664046	-0.010607
42	1	0	5.506597	-3.568930	-0.558327
43	1	0	6.865549	-2.503026	-0.179369
44	6	0	5.526054	-2.858698	1.472726
45	1	0	4.449175	-2.858080	1.656581
46	1	0	5.924901	-3.829811	1.792660
47	7	0	2.497496	0.226376	-0.573283
48	8	0	4.630888	2.341818	-1.019099
49	8	0	8.106951	1.415863	-1.536980
50	8	0	7.802471	0.051016	0.971159
51	8	0	6.063504	-1.814403	2.263738
52	8	0	5.130461	-1.531595	-0.535871
53	6	0	-5.090185	-1.288017	-0.261925
54	6	0	-4.090538	0.694803	0.098080
55	7	0	-5.266788	0.024174	0.062214
56	6	0	-6.402702	0.744718	0.279576
57	6	0	-7.572005	0.022300	0.205285
58	6	0	-7.480269	-1.362005	-0.098341

59	6	0	-6.275712	-2.026112	-0.337515
60	1	0	-8.535503	0.484483	0.362131
61	1	0	-6.273134	-3.079602	-0.580904
62	6	0	-4.474452	2.069624	0.368731
63	6	0	-5.902632	2.095932	0.493775
64	6	0	-3.753444	3.261974	0.491279
65	6	0	-6.578182	3.287619	0.756793
66	6	0	-5.840635	4.453105	0.886014
67	6	0	-4.443730	4.436326	0.747769
68	1	0	-3.891971	5.364051	0.841650
69	1	0	-7.657553	3.298754	0.852335
70	1	0	-2.677531	3.278830	0.375498
71	7	0	-3.771456	-1.508643	-0.429317
72	6	0	-3.146833	-0.319424	-0.210959
73	1	0	-6.345306	5.389478	1.089182
74	6	0	-8.721357	-2.188300	-0.189671
75	8	0	-8.731494	-3.366925	-0.452952
76	8	0	-9.825795	-1.483103	0.052373
77	6	0	-11.061361	-2.207056	-0.016700
78	1	0	-11.837983	-1.482366	0.208044
79	1	0	-11.197822	-2.618789	-1.015997
80	1	0	-11.063893	-3.012870	0.716357

19 (S_1) M06-2X

Center Number	Atomic Number	Atomic Type	Coordinates (Angstroms) X	Y	Z
1	6	0	-1.670601	-0.277856	-0.340993
2	6	0	-0.937972	0.762678	0.250155
3	6	0	-0.943248	-1.257489	-1.037027
4	6	0	0.439523	0.840017	0.143243
5	1	0	-1.451792	1.520082	0.827902
6	6	0	0.433292	-1.194834	-1.154329
7	1	0	-1.477029	-2.083558	-1.489820
8	6	0	1.176377	-0.135975	-0.572649
9	1	0	0.942780	1.675535	0.607648
10	1	0	0.937314	-1.988619	-1.686491
11	6	0	7.227887	-1.300849	2.702754
12	1	0	7.721928	-2.069992	2.100645
13	1	0	7.357031	-1.578490	3.757965
14	6	0	7.875639	0.051127	2.458460
15	1	0	8.909963	0.028975	2.826035
16	1	0	7.334059	0.819826	3.014030
17	6	0	8.793775	-0.162827	0.246055
18	1	0	8.993936	-1.195776	0.555515
19	1	0	9.739987	0.392025	0.297126
20	6	0	8.275218	-0.200494	-1.177424
21	1	0	7.304038	-0.703828	-1.195298
22	1	0	8.977780	-0.779998	-1.787260
23	6	0	6.866120	1.466022	-2.197967
24	1	0	6.410589	0.637572	-2.754252
25	1	0	6.995396	2.304474	-2.885121
26	6	0	5.965776	1.854537	-1.033628
27	1	0	6.331217	2.774382	-0.557922
28	1	0	5.992188	1.061285	-0.285050
29	6	0	3.678069	2.170219	-0.482793
30	1	0	2.788846	2.609185	-0.938583
31	1	0	4.047032	2.858890	0.289570
32	6	0	3.335068	0.824689	0.159894
33	1	0	2.799358	1.000571	1.093530

34	1	0	4.247953	0.293366	0.430989
35	6	0	3.273993	-0.912440	-1.625646
36	1	0	4.087908	-0.324547	-2.052337
37	1	0	2.617092	-1.179318	-2.452377
38	6	0	3.862865	-2.174272	-1.005143
39	1	0	4.032879	-2.933250	-1.780820
40	1	0	3.169388	-2.589218	-0.264567
41	6	0	5.609808	-2.799889	0.518173
42	1	0	5.222865	-3.798008	0.281000
43	1	0	6.693366	-2.830840	0.381214
44	6	0	5.260105	-2.450928	1.959254
45	1	0	4.180094	-2.320861	2.057193
46	1	0	5.564437	-3.273822	2.620168
47	7	0	2.544214	-0.060686	-0.691740
48	8	0	4.643620	2.055227	-1.529427
49	8	0	8.169879	1.110604	-1.745822
50	8	0	7.828351	0.449989	1.093594
51	8	0	5.841749	-1.221082	2.384315
52	8	0	5.107398	-1.829591	-0.396154
53	6	0	-5.108171	-1.271110	-0.254755
54	6	0	-4.034806	0.691823	0.090325
55	7	0	-5.236493	0.053212	0.069342
56	6	0	-6.351043	0.811353	0.295644
57	6	0	-7.544926	0.120539	0.233453
58	6	0	-7.506431	-1.268067	-0.066857
59	6	0	-6.314110	-1.969810	-0.316753
60	1	0	-8.489879	0.614340	0.398206
61	1	0	-6.345826	-3.022214	-0.558109
62	6	0	-4.374543	2.074665	0.361797
63	6	0	-5.812009	2.144721	0.500818
64	6	0	-3.620418	3.248193	0.480657
65	6	0	-6.444918	3.359937	0.768407
66	6	0	-5.669674	4.505480	0.890868
67	6	0	-4.273375	4.446103	0.742678
68	1	0	-3.692174	5.356189	0.833131
69	1	0	-7.522289	3.406775	0.873736
70	1	0	-2.545652	3.234851	0.360395
71	7	0	-3.792865	-1.531069	-0.436535
72	6	0	-3.117366	-0.355285	-0.229171
73	1	0	-6.144572	5.456839	1.097333
74	6	0	-8.768937	-2.052732	-0.143995
75	8	0	-8.823649	-3.240447	-0.397520
76	8	0	-9.856863	-1.304414	0.098234
77	6	0	-11.129420	-1.982109	0.043131
78	1	0	-11.871388	-1.218329	0.259622
79	1	0	-11.289624	-2.402410	-0.949619
80	1	0	-11.166356	-2.775405	0.789620

19·Ba(ClO$_4$)$_2$ (S$_0$) M06-2X

Center Number	Atomic Number	Atomic Type	Coordinates (Angstroms) X	Y	Z
1	6	0	-0.263463	2.216085	-1.469542
2	6	0	0.084225	3.069925	-0.417542
3	6	0	0.729456	1.859349	-2.381960
4	6	0	1.406209	3.408228	-0.190106
5	1	0	-0.673564	3.370581	0.296479
6	6	0	2.050704	2.231823	-2.185333
7	1	0	0.484133	1.206085	-3.211889
8	6	0	2.439924	2.916743	-1.018463

9	1	0	1.630607	4.007342	0.680147
10	1	0	2.778744	1.904619	-2.909020
11	6	0	3.239699	-3.303481	1.244500
12	1	0	3.516423	-3.157398	2.297858
13	1	0	3.875206	-4.093943	0.825906
14	6	0	1.794369	-3.717304	1.097467
15	1	0	1.609376	-4.630794	1.673112
16	1	0	1.577930	-3.905925	0.047113
17	6	0	0.567802	-2.634100	2.882019
18	1	0	0.715160	-3.614905	3.347509
19	1	0	-0.486749	-2.374289	2.925444
20	6	0	1.374072	-1.595364	3.644940
21	1	0	2.400536	-1.929080	3.847561
22	1	0	0.870854	-1.408437	4.601678
23	6	0	1.875415	0.705609	3.653746
24	1	0	2.806827	0.434195	4.170208
25	1	0	1.110408	0.972332	4.393282
26	6	0	2.118470	1.874168	2.721191
27	1	0	2.528798	2.713753	3.292164
28	1	0	1.174047	2.186008	2.272637
29	6	0	4.241621	2.184358	1.601701
30	1	0	4.976710	1.480764	1.218301
31	1	0	4.552467	2.515906	2.600757
32	6	0	4.168929	3.395848	0.668356
33	1	0	5.162393	3.852846	0.640214
34	1	0	3.504977	4.156987	1.078763
35	6	0	4.813519	2.593763	-1.593876
36	1	0	5.759806	2.939038	-1.174751
37	1	0	4.706422	3.081447	-2.570006
38	6	0	4.922370	1.078430	-1.843904
39	1	0	5.957135	0.886636	-2.161904
40	1	0	4.267391	0.718687	-2.639990
41	6	0	5.147381	-0.968347	-0.755204
42	1	0	4.724712	-1.486567	-1.621349
43	1	0	6.241395	-0.928978	-0.857775
44	6	0	4.815149	-1.729905	0.500278
45	1	0	5.431492	-2.635783	0.510613
46	1	0	5.054491	-1.134723	1.391594
47	7	0	3.775779	3.078286	-0.696033
48	8	0	3.015324	1.472398	1.688131
49	8	0	1.426707	-0.397890	2.888276
50	8	0	0.911604	-2.685442	1.503864
51	8	0	3.444023	-2.097521	0.523930
52	8	0	4.643008	0.352241	-0.665396
53	56	0	1.185201	-0.261818	0.096774
54	6	0	-2.833034	1.836045	-1.149048
55	6	0	-2.632367	-0.403073	-1.264095
56	6	0	-4.780186	0.675751	-0.678481
57	6	0	-3.213601	-1.662537	-1.087497
58	1	0	-2.639314	-2.572371	-1.197097
59	6	0	-5.359603	-0.556853	-0.503479
60	1	0	-6.383832	-0.665727	-0.179203
61	6	0	-4.556755	-1.710073	-0.723526
62	6	0	-5.046126	2.101620	-0.513004
63	6	0	-3.841412	2.816285	-0.801587
64	6	0	-6.199536	2.784226	-0.134242
65	1	0	-7.111751	2.243139	0.088230
66	6	0	-3.816087	4.207816	-0.707657
67	6	0	-4.974678	4.868434	-0.329569
68	1	0	-4.970411	5.949283	-0.252740
69	6	0	-6.155033	4.166779	-0.044389
70	1	0	-2.909657	4.757980	-0.930271
71	7	0	-3.478994	0.644824	-1.078963

72	1	0	-7.041903	4.714332	0.250058
73	7	0	-1.398831	0.093247	-1.493154
74	6	0	-1.512620	1.452186	-1.424415
75	6	0	-5.137554	-3.071442	-0.526124
76	8	0	-4.529952	-4.099815	-0.665286
77	8	0	-6.432235	-3.030491	-0.170935
78	6	0	-7.043714	-4.303689	0.040132
79	1	0	-8.072959	-4.093974	0.318858
80	1	0	-7.004342	-4.897867	-0.873069
81	1	0	-6.529985	-4.841644	0.837107
82	17	0	2.001261	-2.211552	-2.621255
83	17	0	-1.666077	0.261342	2.118008
84	8	0	3.116767	-3.146606	-2.472651
85	8	0	2.482845	-0.816284	-2.338954
86	8	0	0.972172	-2.483259	-1.575976
87	8	0	1.420777	-2.278097	-3.947340
88	8	0	-1.286173	-0.972201	1.379208
89	8	0	-0.734549	1.324092	1.625476
90	8	0	-1.470317	0.060819	3.555777
91	8	0	-3.041287	0.630239	1.811704

19·Ba(ClO$_4$)$_2$ (S$_1$) M06-2X

Center Number	Atomic Number	Atomic Type	Coordinates (Angstroms) X	Y	Z
1	6	0	-0.368426	2.475186	-1.322086
2	6	0	0.007083	3.208431	-0.187168
3	6	0	0.587005	2.292291	-2.327565
4	6	0	1.332887	3.551111	0.025808
5	1	0	-0.718324	3.387760	0.595399
6	6	0	1.905101	2.683499	-2.143247
7	1	0	0.317807	1.756979	-3.229236
8	6	0	2.338264	3.185391	-0.898411
9	1	0	1.589477	4.017927	0.964546
10	1	0	2.606258	2.494443	-2.939017
11	6	0	3.516740	-3.323247	0.977044
12	1	0	3.855573	-3.195775	2.013850
13	1	0	4.148334	-4.087376	0.504955
14	6	0	2.079447	-3.795503	0.902631
15	1	0	1.977693	-4.748327	1.434557
16	1	0	1.800208	-3.935651	-0.141146
17	6	0	0.866924	-2.880523	2.819296
18	1	0	0.993658	-3.898410	3.204028
19	1	0	-0.176652	-2.588473	2.909043
20	6	0	1.729843	-1.918875	3.620258
21	1	0	2.771885	-2.257306	3.697130
22	1	0	1.315194	-1.845492	4.633826
23	6	0	2.262081	0.397521	3.764418
24	1	0	3.304264	0.149590	4.012255
25	1	0	1.694057	0.518451	4.695660
26	6	0	2.187237	1.676462	2.955573
27	1	0	2.611583	2.505268	3.530733
28	1	0	1.145020	1.900331	2.729439
29	6	0	4.163797	2.134724	1.610213
30	1	0	4.819690	1.419633	1.119946
31	1	0	4.553646	2.350954	2.612403
32	6	0	4.122286	3.430723	0.793512
33	1	0	5.134644	3.846883	0.784747
34	1	0	3.496713	4.173981	1.288576
35	6	0	4.699953	2.821544	-1.545685
36	1	0	5.667803	3.084888	-1.115774

37	1	0	4.604288	3.401388	-2.471972
38	6	0	4.742387	1.342782	-1.956631
39	1	0	5.674091	1.208594	-2.526214
40	1	0	3.914408	1.054433	-2.605036
41	6	0	5.136778	-0.838204	-1.172796
42	1	0	4.525351	-1.217013	-1.994984
43	1	0	6.190471	-0.831098	-1.489065
44	6	0	5.008316	-1.756066	0.016156
45	1	0	5.563035	-2.672984	-0.215357
46	1	0	5.453091	-1.301133	0.911453
47	7	0	3.690326	3.266287	-0.589369
48	8	0	2.877575	1.512828	1.711205
49	8	0	1.694542	-0.647551	2.984131
50	8	0	1.153735	-2.842178	1.419565
51	8	0	3.643379	-2.092889	0.268697
52	8	0	4.745021	0.485576	-0.821853
53	56	0	1.157387	-0.358529	0.155087
54	6	0	-2.919030	1.922261	-1.028762
55	6	0	-2.579515	-0.308866	-1.209843
56	6	0	-4.803868	0.622642	-0.627794
57	6	0	-3.082611	-1.603549	-1.089246
58	1	0	-2.453006	-2.471005	-1.223522
59	6	0	-5.306034	-0.654156	-0.506281
60	1	0	-6.326929	-0.836403	-0.208671
61	6	0	-4.433012	-1.752252	-0.747529
62	6	0	-5.162811	2.020662	-0.426225
63	6	0	-3.991216	2.823834	-0.671977
64	6	0	-6.360100	2.623408	-0.044090
65	1	0	-7.242428	2.023611	0.146205
66	6	0	-4.054873	4.210533	-0.526707
67	6	0	-5.260045	4.787955	-0.145947
68	1	0	-5.321801	5.863753	-0.029545
69	6	0	-6.400991	4.005629	0.093637
70	1	0	-3.179479	4.822333	-0.708262
71	7	0	-3.491748	0.684811	-1.000587
72	1	0	-7.324281	4.487617	0.391897
73	7	0	-1.372540	0.278298	-1.402050
74	6	0	-1.569903	1.637069	-1.302241
75	6	0	-4.926981	-3.149285	-0.601598
76	8	0	-4.250614	-4.139999	-0.753656
77	8	0	-6.238199	-3.199459	-0.272753
78	6	0	-6.784196	-4.517769	-0.104180
79	1	0	-7.829933	-4.367763	0.154709
80	1	0	-6.693616	-5.088302	-1.029387
81	1	0	-6.261397	-5.046311	0.693955
82	17	0	1.732565	-1.977750	-2.852297
83	17	0	-1.632866	0.117351	2.289092
84	8	0	2.929309	-2.842321	-2.991287
85	8	0	2.176615	-0.574609	-2.444474
86	8	0	0.878311	-2.465667	-1.697289
87	8	0	0.952136	-1.922026	-4.094278
88	8	0	-1.240104	-1.134499	1.534580
89	8	0	-0.686211	1.197084	1.787130
90	8	0	-1.434920	-0.086913	3.743253
91	8	0	-3.023865	0.488427	1.974072

----

19 (S_0) PBE

Center Number	Atomic Number	Atomic Type	Coordinates (Angstroms) X	Y	Z
1	6	0	-1.739112	-0.251673	-0.310519
2	6	0	-1.005575	0.752025	0.331707
3	6	0	-1.013477	-1.202228	-1.040633
4	6	0	0.371640	0.822682	0.240977
5	1	0	-1.518264	1.488329	0.941433
6	6	0	0.362330	-1.145544	-1.143428
7	1	0	-1.549642	-2.005358	-1.534662
8	6	0	1.107310	-0.122433	-0.510377
9	1	0	0.876208	1.633296	0.750923
10	1	0	0.864170	-1.921874	-1.707008
11	6	0	7.641569	-1.761316	2.313258
12	1	0	8.052508	-2.344574	1.479413
13	1	0	7.950132	-2.259350	3.244753
14	6	0	8.203544	-0.356639	2.285128
15	1	0	9.275295	-0.386689	2.525295
16	1	0	7.705095	0.245468	3.050517
17	6	0	8.945122	0.013102	0.058835
18	1	0	9.235595	-1.046758	0.076608
19	1	0	9.852129	0.610778	0.232732
20	6	0	8.375434	0.305173	-1.307306
21	1	0	7.445718	-0.261656	-1.433534
22	1	0	9.090567	-0.042285	-2.064549
23	6	0	6.843486	2.018633	-1.948263
24	1	0	6.504829	1.312123	-2.718384
25	1	0	6.907635	3.008231	-2.408157
26	6	0	5.857717	2.028166	-0.793959
27	1	0	6.091322	2.854926	-0.106775
28	1	0	5.960550	1.092711	-0.231845
29	6	0	3.557898	2.192648	-0.302973
30	1	0	2.662130	2.620472	-0.760906
31	1	0	3.870835	2.854063	0.518777
32	6	0	3.250283	0.808622	0.262284
33	1	0	2.728931	0.921057	1.216440
34	1	0	4.182644	0.286856	0.491948
35	6	0	3.205714	-0.869902	-1.560656
36	1	0	4.025644	-0.267868	-1.963053
37	1	0	2.556559	-1.104585	-2.405517
38	6	0	3.797915	-2.154192	-0.998051
39	1	0	3.928653	-2.884787	-1.810262
40	1	0	3.121017	-2.592974	-0.251740
41	6	0	5.693262	-2.942591	0.211611
42	1	0	5.257940	-3.895681	-0.115829
43	1	0	6.739781	-2.939516	-0.111772
44	6	0	5.607163	-2.850003	1.723533
45	1	0	4.559287	-2.791633	2.031979
46	1	0	6.038405	-3.759315	2.167967
47	7	0	2.472579	-0.051920	-0.612126
48	8	0	4.551805	2.183067	-1.313115
49	8	0	8.143783	1.692703	-1.499227
50	8	0	7.980040	0.296008	1.050583
51	8	0	6.231947	-1.691092	2.240091
52	8	0	5.056780	-1.849702	-0.425301
53	6	0	-5.156107	-1.250149	-0.280980
54	6	0	-4.109692	0.698697	0.122684
55	7	0	-5.299304	0.056727	0.073878
56	6	0	-6.415690	0.795038	0.314423
57	6	0	-7.602310	0.098060	0.228015
58	6	0	-7.547669	-1.277111	-0.108406

59	6	0	-6.352325	-1.959276	-0.369109
60	1	0	-8.554913	0.578417	0.402076
61	1	0	-6.374331	-3.007342	-0.637738
62	6	0	-4.459795	2.067426	0.431178
63	6	0	-5.890477	2.122092	0.561574
64	6	0	-3.716059	3.240621	0.589391
65	6	0	-6.536066	3.321263	0.860637
66	6	0	-5.773424	4.465745	1.021927
67	6	0	-4.378791	4.421822	0.881464
68	1	0	-3.804708	5.334600	1.002651
69	1	0	-7.615860	3.356229	0.960528
70	1	0	-2.638592	3.240261	0.475774
71	7	0	-3.845332	-1.499083	-0.457790
72	6	0	-3.186561	-0.332239	-0.214047
73	1	0	-6.258095	5.407940	1.253731
74	6	0	-8.799144	-2.073262	-0.213501
75	8	0	-8.835360	-3.258168	-0.502082
76	8	0	-9.890066	-1.347674	0.044064
77	6	0	-11.140374	-2.038208	-0.035603
78	1	0	-11.899759	-1.296005	0.200120
79	1	0	-11.293089	-2.433274	-1.041171
80	1	0	-11.169774	-2.855721	0.686493

19 (S_1) PBE

Center Number	Atomic Number	Atomic Type	Coordinates (Angstroms) X	Y	Z
1	6	0	-1.670601	-0.277856	-0.340993
2	6	0	-0.937972	0.762678	0.250155
3	6	0	-0.943248	-1.257489	-1.037027
4	6	0	0.439523	0.840017	0.143243
5	1	0	-1.451792	1.520082	0.827902
6	6	0	0.433292	-1.194834	-1.154329
7	1	0	-1.477029	-2.083558	-1.489820
8	6	0	1.176377	-0.135975	-0.572649
9	1	0	0.942780	1.675535	0.607648
10	1	0	0.937314	-1.988619	-1.686491
11	6	0	7.227887	-1.300849	2.702754
12	1	0	7.721928	-2.069992	2.100645
13	1	0	7.357031	-1.578490	3.757965
14	6	0	7.875639	0.051127	2.458460
15	1	0	8.909963	0.028975	2.826035
16	1	0	7.334059	0.819826	3.014030
17	6	0	8.793775	-0.162827	0.246055
18	1	0	8.993936	-1.195776	0.555515
19	1	0	9.739987	0.392025	0.297126
20	6	0	8.275218	-0.200494	-1.177424
21	1	0	7.304038	-0.703828	-1.195298
22	1	0	8.977780	-0.779998	-1.787260
23	6	0	6.866120	1.466022	-2.197967
24	1	0	6.410589	0.637572	-2.754252
25	1	0	6.995396	2.304474	-2.885121
26	6	0	5.965776	1.854537	-1.033628
27	1	0	6.331217	2.774382	-0.557922
28	1	0	5.992188	1.061285	-0.285050
29	6	0	3.678069	2.170219	-0.482793
30	1	0	2.788846	2.609185	-0.938583
31	1	0	4.047032	2.858890	0.289570
32	6	0	3.335068	0.824689	0.159894
33	1	0	2.799358	1.000571	1.093530
34	1	0	4.247953	0.293366	0.430989

35	6	0	3.273993	-0.912440	-1.625646
36	1	0	4.087908	-0.324547	-2.052337
37	1	0	2.617092	-1.179318	-2.452377
38	6	0	3.862865	-2.174272	-1.005143
39	1	0	4.032879	-2.933250	-1.780820
40	1	0	3.169388	-2.589218	-0.264567
41	6	0	5.609808	-2.799889	0.518173
42	1	0	5.222865	-3.798008	0.281000
43	1	0	6.693366	-2.830840	0.381214
44	6	0	5.260105	-2.450928	1.959254
45	1	0	4.180094	-2.320861	2.057193
46	1	0	5.564437	-3.273822	2.620168
47	7	0	2.544214	-0.060686	-0.691740
48	8	0	4.643620	2.055227	-1.529427
49	8	0	8.169879	1.110604	-1.745822
50	8	0	7.828351	0.449989	1.093594
51	8	0	5.841749	-1.221082	2.384315
52	8	0	5.107398	-1.829591	-0.396154
53	6	0	-5.108171	-1.271110	-0.254755
54	6	0	-4.034806	0.691823	0.090325
55	7	0	-5.236493	0.053212	0.069342
56	6	0	-6.351043	0.811353	0.295644
57	6	0	-7.544926	0.120539	0.233453
58	6	0	-7.506431	-1.268067	-0.066857
59	6	0	-6.314110	-1.969810	-0.316753
60	1	0	-8.489879	0.614340	0.398206
61	1	0	-6.345826	-3.022214	-0.558109
62	6	0	-4.374543	2.074665	0.361797
63	6	0	-5.812009	2.144721	0.500818
64	6	0	-3.620418	3.248193	0.480657
65	6	0	-6.444918	3.359937	0.768407
66	6	0	-5.669674	4.505480	0.890868
67	6	0	-4.273375	4.446103	0.742678
68	1	0	-3.692174	5.356189	0.833131
69	1	0	-7.522289	3.406775	0.873736
70	1	0	-2.545652	3.234851	0.360395
71	7	0	-3.792865	-1.531069	-0.436535
72	6	0	-3.117366	-0.355285	-0.229171
73	1	0	-6.144572	5.456839	1.097333
74	6	0	-8.768937	-2.052732	-0.143995
75	8	0	-8.823649	-3.240447	-0.397520
76	8	0	-9.856863	-1.304414	0.098234
77	6	0	-11.129420	-1.982109	0.043131
78	1	0	-11.871388	-1.218329	0.259622
79	1	0	-11.289624	-2.402410	-0.949619
80	1	0	-11.166356	-2.775405	0.789620

19·Ba(ClO$_4$)$_2$ (S_0) PBE

Center Number	Atomic Number	Atomic Type	Coordinates (Angstroms) X	Y	Z
1	6	0	-0.499092	2.346653	-1.111516
2	6	0	-0.061884	3.107079	-0.021732
3	6	0	0.392546	2.152563	-2.166863
4	6	0	1.261523	3.492088	0.085746
5	1	0	-0.738369	3.297340	0.804250
6	6	0	1.707454	2.579771	-2.085945
7	1	0	0.074409	1.595506	-3.041350
8	6	0	2.210282	3.139980	-0.898116
9	1	0	1.568288	3.998085	0.991020

10	1	0	2.357016	2.377375	-2.924210
11	6	0	3.952487	-3.159138	0.759763
12	1	0	4.346068	-3.002407	1.775666
13	1	0	4.593012	-3.907352	0.268235
14	6	0	2.546599	-3.712421	0.775355
15	1	0	2.541865	-4.678891	1.295983
16	1	0	2.212625	-3.864289	-0.252964
17	6	0	1.381255	-2.946927	2.760575
18	1	0	1.598778	-3.965579	3.105010
19	1	0	0.321690	-2.738791	2.914735
20	6	0	2.203788	-1.950972	3.549505
21	1	0	3.275269	-2.201406	3.548991
22	1	0	1.852660	-1.956989	4.591767
23	6	0	2.616301	0.364293	3.714458
24	1	0	3.691863	0.162014	3.839631
25	1	0	2.156656	0.437367	4.710859
26	6	0	2.394440	1.653052	2.960149
27	1	0	2.823768	2.491880	3.521310
28	1	0	1.319827	1.820799	2.849922
29	6	0	4.212381	2.202694	1.466424
30	1	0	4.855836	1.498510	0.938020
31	1	0	4.668101	2.440089	2.437529
32	6	0	4.091085	3.483678	0.644821
33	1	0	5.093161	3.917938	0.559026
34	1	0	3.490973	4.225572	1.178413
35	6	0	4.522498	2.982847	-1.745600
36	1	0	5.488460	3.369455	-1.409827
37	1	0	4.263745	3.549511	-2.650373
38	6	0	4.713433	1.529270	-2.180865
39	1	0	5.584626	1.522291	-2.857116
40	1	0	3.859825	1.141655	-2.745778
41	6	0	5.283826	-0.624709	-1.506873
42	1	0	4.573171	-0.979824	-2.261677
43	1	0	6.291145	-0.614964	-1.952800
44	6	0	5.290825	-1.586075	-0.349863
45	1	0	5.831951	-2.484761	-0.677771
46	1	0	5.834050	-1.158958	0.506863
47	7	0	3.568892	3.295294	-0.695664
48	8	0	2.953568	1.568938	1.657871
49	8	0	2.015871	-0.676259	2.974356
50	8	0	1.594935	-2.840564	1.361531
51	8	0	3.976160	-1.950972	0.029845
52	8	0	4.946784	0.681196	-1.079512
53	56	0	1.103041	-0.360177	0.160599
54	6	0	-3.057692	1.872700	-0.788151
55	6	0	-2.790929	-0.353260	-0.974204
56	6	0	-5.000026	0.651745	-0.509511
57	6	0	-3.353979	-1.626725	-0.932548
58	1	0	-2.762234	-2.520974	-1.079110
59	6	0	-5.564826	-0.602296	-0.466886
60	1	0	-6.617008	-0.748682	-0.266849
61	6	0	-4.724716	-1.724820	-0.681873
62	6	0	-5.324676	2.059899	-0.358375
63	6	0	-4.118920	2.815854	-0.532650
64	6	0	-6.528248	2.706296	-0.089186
65	1	0	-7.442567	2.138045	0.047060
66	6	0	-4.149968	4.206242	-0.436109
67	6	0	-5.359312	4.827843	-0.169915
68	1	0	-5.396659	5.909665	-0.093085
69	6	0	-6.536672	4.088632	0.003590
70	1	0	-3.244737	4.788808	-0.569164
71	7	0	-3.666147	0.663460	-0.766269
72	1	0	-7.466067	4.607060	0.213618

73	7	0	-1.564300	0.186479	-1.142793
74	6	0	-1.717358	1.539204	-1.039323
75	6	0	-5.286332	-3.101108	-0.638973
76	8	0	-4.645353	-4.107104	-0.811061
77	8	0	-6.604716	-3.107672	-0.384471
78	6	0	-7.204213	-4.399734	-0.322685
79	1	0	-8.257757	-4.225853	-0.110523
80	1	0	-7.085423	-4.923175	-1.273497
81	1	0	-6.746973	-4.996105	0.469351
82	17	0	1.696229	-1.928960	-2.871380
83	17	0	-1.471439	-0.124890	2.507758
84	8	0	2.901052	-2.719677	-3.122600
85	8	0	2.089191	-0.530319	-2.472193
86	8	0	0.967240	-2.471658	-1.680654
87	8	0	0.827049	-1.890825	-4.033800
88	8	0	-1.037633	-1.325891	1.724823
89	8	0	-0.606008	0.998618	2.010133
90	8	0	-1.230678	-0.345101	3.931774
91	8	0	-2.870357	0.175241	2.236983

19·Ba(ClO$_4$)$_2$ (S$_1$) PBE

Center Number	Atomic Number	Atomic Type	Coordinates (Angstroms) X	Y	Z
1	6	0	-0.368426	2.475186	-1.322086
2	6	0	0.007083	3.208431	-0.187168
3	6	0	0.587005	2.292291	-2.327565
4	6	0	1.332887	3.551111	0.025808
5	1	0	-0.718324	3.387760	0.595399
6	6	0	1.905101	2.683499	-2.143247
7	1	0	0.317807	1.756979	-3.229236
8	6	0	2.338264	3.185391	-0.898411
9	1	0	1.589477	4.017927	0.964546
10	1	0	2.606258	2.494443	-2.939017
11	6	0	3.516740	-3.323247	0.977044
12	1	0	3.855573	-3.195775	2.013850
13	1	0	4.148334	-4.087376	0.504955
14	6	0	2.079447	-3.795503	0.902631
15	1	0	1.977693	-4.748327	1.434557
16	1	0	1.800208	-3.935651	-0.141146
17	6	0	0.866924	-2.880523	2.819296
18	1	0	0.993658	-3.898410	3.204028
19	1	0	-0.176652	-2.588473	2.909043
20	6	0	1.729843	-1.918875	3.620258
21	1	0	2.771885	-2.257306	3.697130
22	1	0	1.315194	-1.845492	4.633826
23	6	0	2.262081	0.397521	3.764418
24	1	0	3.304264	0.149590	4.012255
25	1	0	1.694057	0.518451	4.695660
26	6	0	2.187237	1.676462	2.955573
27	1	0	2.611583	2.505268	3.530733
28	1	0	1.145020	1.900331	2.729439
29	6	0	4.163797	2.134724	1.610213
30	1	0	4.819690	1.419633	1.119946
31	1	0	4.553646	2.350954	2.612403
32	6	0	4.122286	3.430723	0.793512
33	1	0	5.134644	3.846883	0.784747
34	1	0	3.496713	4.173981	1.288576
35	6	0	4.699953	2.821544	-1.545685
36	1	0	5.667803	3.084888	-1.115774

37	1	0	4.604288	3.401388	-2.471972
38	6	0	4.742387	1.342782	-1.956631
39	1	0	5.674091	1.208594	-2.526214
40	1	0	3.914408	1.054433	-2.605036
41	6	0	5.136778	-0.838204	-1.172796
42	1	0	4.525351	-1.217013	-1.994984
43	1	0	6.190471	-0.831098	-1.489065
44	6	0	5.008316	-1.756066	0.016156
45	1	0	5.563035	-2.672984	-0.215357
46	1	0	5.453091	-1.301133	0.911453
47	7	0	3.690326	3.266287	-0.589369
48	8	0	2.877575	1.512828	1.711205
49	8	0	1.694542	-0.647551	2.984131
50	8	0	1.153735	-2.842178	1.419565
51	8	0	3.643379	-2.092889	0.268697
52	8	0	4.745021	0.485576	-0.821853
53	56	0	1.157387	-0.358529	0.155087
54	6	0	-2.919030	1.922261	-1.028762
55	6	0	-2.579515	-0.308866	-1.209843
56	6	0	-4.803868	0.622642	-0.627794
57	6	0	-3.082611	-1.603549	-1.089246
58	1	0	-2.453006	-2.471005	-1.223522
59	6	0	-5.306034	-0.654156	-0.506281
60	1	0	-6.326929	-0.836403	-0.208671
61	6	0	-4.433012	-1.752252	-0.747529
62	6	0	-5.162811	2.020662	-0.426225
63	6	0	-3.991216	2.823834	-0.671977
64	6	0	-6.360100	2.623408	-0.044090
65	1	0	-7.242428	2.023611	0.146205
66	6	0	-4.054873	4.210533	-0.526707
67	6	0	-5.260045	4.787955	-0.145947
68	1	0	-5.321801	5.863753	-0.029545
69	6	0	-6.400991	4.005629	0.093637
70	1	0	-3.179479	4.822333	-0.708262
71	7	0	-3.491748	0.684811	-1.000587
72	1	0	-7.324281	4.487617	0.391897
73	7	0	-1.372540	0.278298	-1.402050
74	6	0	-1.569903	1.637069	-1.302241
75	6	0	-4.926981	-3.149285	-0.601598
76	8	0	-4.250614	-4.139999	-0.753656
77	8	0	-6.238199	-3.199459	-0.272753
78	6	0	-6.784196	-4.517769	-0.104180
79	1	0	-7.829933	-4.367763	0.154709
80	1	0	-6.693616	-5.088302	-1.029387
81	1	0	-6.261397	-5.046311	0.693955
82	17	0	1.732565	-1.977750	-2.852297
83	17	0	-1.632866	0.117351	2.289092
84	8	0	2.929309	-2.842321	-2.991287
85	8	0	2.176615	-0.574609	-2.444474
86	8	0	0.878311	-2.465667	-1.697289
87	8	0	0.952136	-1.922026	-4.094278
88	8	0	-1.240104	-1.134499	1.534580
89	8	0	-0.686211	1.197084	1.787130
90	8	0	-1.434920	-0.086913	3.743253
91	8	0	-3.023865	0.488427	1.974072

----

19 (S_0) wB97XD

Center Number	Atomic Number	Atomic Type	Coordinates (Angstroms) X	Y	Z
1	6	0	-1.683357	-0.240507	-0.304224
2	6	0	-0.977134	0.747933	0.385299
3	6	0	-0.945056	-1.131547	-1.087379
4	6	0	0.397648	0.861617	0.287703
5	1	0	-1.507894	1.437469	1.032850
6	6	0	0.430156	-1.034218	-1.193152
7	1	0	-1.462775	-1.920370	-1.621141
8	6	0	1.148667	-0.025671	-0.513375
9	1	0	0.885652	1.661472	0.828427
10	1	0	0.948680	-1.766849	-1.797034
11	6	0	7.353967	-1.679164	2.481047
12	1	0	7.824744	-2.373424	1.775840
13	1	0	7.520044	-2.081804	3.489696
14	6	0	8.005337	-0.311955	2.381217
15	1	0	9.057504	-0.392607	2.686940
16	1	0	7.506401	0.377558	3.066153
17	6	0	8.787805	-0.237499	0.125240
18	1	0	8.973741	-1.310069	0.267321
19	1	0	9.753297	0.281119	0.202571
20	6	0	8.196255	-0.055433	-1.255756
21	1	0	7.214405	-0.540654	-1.291063
22	1	0	8.852244	-0.549085	-1.982386
23	6	0	6.787285	1.749061	-1.954335
24	1	0	6.308122	1.034729	-2.637401
25	1	0	6.903303	2.696531	-2.484966
26	6	0	5.920106	1.929889	-0.719341
27	1	0	6.333016	2.720891	-0.078104
28	1	0	5.926952	0.999134	-0.146147
29	6	0	3.672237	2.243434	-0.071166
30	1	0	2.788517	2.774100	-0.432515
31	1	0	4.072876	2.789758	0.794768
32	6	0	3.292630	0.828715	0.361066
33	1	0	2.736187	0.884389	1.298581
34	1	0	4.191059	0.247951	0.582378
35	6	0	3.253984	-0.636508	-1.642502
36	1	0	4.069679	0.001497	-1.990040
37	1	0	2.605573	-0.801195	-2.503112
38	6	0	3.853025	-1.959204	-1.181824
39	1	0	3.984727	-2.635546	-2.037791
40	1	0	3.182122	-2.445530	-0.461572
41	6	0	5.679045	-2.771119	0.117981
42	1	0	5.314985	-3.729162	-0.274091
43	1	0	6.758018	-2.737552	-0.058916
44	6	0	5.381011	-2.691514	1.608023
45	1	0	4.303176	-2.597384	1.763075
46	1	0	5.712181	-3.618700	2.095810
47	7	0	2.515341	0.090445	-0.623423
48	8	0	4.610314	2.269930	-1.134508
49	8	0	8.089382	1.318041	-1.608357
50	8	0	7.891990	0.263364	1.096389
51	8	0	5.967319	-1.561491	2.226003
52	8	0	5.115231	-1.688063	-0.595353
53	6	0	-5.097239	-1.283530	-0.232773
54	6	0	-4.060551	0.681048	0.091586
55	7	0	-5.247807	0.033159	0.069962
56	6	0	-6.367187	0.776530	0.278911
57	6	0	-7.548104	0.076870	0.217823
58	6	0	-7.487226	-1.313670	-0.064336

59	6	0	-6.294210	-2.000439	-0.294613
60	1	0	-8.500676	0.562368	0.370015
61	1	0	-6.308502	-3.057572	-0.521406
62	6	0	-4.416454	2.064240	0.344748
63	6	0	-5.841414	2.120099	0.472295
64	6	0	-3.670293	3.239899	0.453721
65	6	0	-6.490213	3.328181	0.721129
66	6	0	-5.728529	4.478767	0.834895
67	6	0	-4.333898	4.431325	0.696872
68	1	0	-3.762078	5.348484	0.780772
69	1	0	-7.569069	3.364777	0.818930
70	1	0	-2.593566	3.229544	0.340148
71	7	0	-3.783458	-1.531114	-0.400273
72	6	0	-3.137152	-0.348142	-0.203927
73	1	0	-6.213785	5.428356	1.027243
74	6	0	-8.743620	-2.116803	-0.140800
75	8	0	-8.776889	-3.301965	-0.381874
76	8	0	-9.831634	-1.384377	0.086281
77	6	0	-11.089708	-2.068627	0.032554
78	1	0	-11.841182	-1.311209	0.239340
79	1	0	-11.244221	-2.498720	-0.957401
80	1	0	-11.124556	-2.855981	0.786058

19 (S_1) wB97XDYP

Center Number	Atomic Number	Atomic Type	Coordinates (Angstroms) X	Y	Z
1	6	0	-1.670601	-0.277856	-0.340993
2	6	0	-0.937972	0.762678	0.250155
3	6	0	-0.943248	-1.257489	-1.037027
4	6	0	0.439523	0.840017	0.143243
5	1	0	-1.451792	1.520082	0.827902
6	6	0	0.433292	-1.194834	-1.154329
7	1	0	-1.477029	-2.083558	-1.489820
8	6	0	1.176377	-0.135975	-0.572649
9	1	0	0.942780	1.675535	0.607648
10	1	0	0.937314	-1.988619	-1.686491
11	6	0	7.227887	-1.300849	2.702754
12	1	0	7.721928	-2.069992	2.100645
13	1	0	7.357031	-1.578490	3.757965
14	6	0	7.875639	0.051127	2.458460
15	1	0	8.909963	0.028975	2.826035
16	1	0	7.334059	0.819826	3.014030
17	6	0	8.793775	-0.162827	0.246055
18	1	0	8.993936	-1.195776	0.555515
19	1	0	9.739987	0.392025	0.297126
20	6	0	8.275218	-0.200494	-1.177424
21	1	0	7.304038	-0.703828	-1.195298
22	1	0	8.977780	-0.779998	-1.787260
23	6	0	6.866120	1.466022	-2.197967
24	1	0	6.410589	0.637572	-2.754252
25	1	0	6.995396	2.304474	-2.885121
26	6	0	5.965776	1.854537	-1.033628
27	1	0	6.331217	2.774382	-0.557922
28	1	0	5.992188	1.061285	-0.285050
29	6	0	3.678069	2.170219	-0.482793
30	1	0	2.788846	2.609185	-0.938583
31	1	0	4.047032	2.858890	0.289570
32	6	0	3.335068	0.824689	0.159894
33	1	0	2.799358	1.000571	1.093530

34	1	0	4.247953	0.293366	0.430989
35	6	0	3.273993	-0.912440	-1.625646
36	1	0	4.087908	-0.324547	-2.052337
37	1	0	2.617092	-1.179318	-2.452377
38	6	0	3.862865	-2.174272	-1.005143
39	1	0	4.032879	-2.933250	-1.780820
40	1	0	3.169388	-2.589218	-0.264567
41	6	0	5.609808	-2.799889	0.518173
42	1	0	5.222865	-3.798008	0.281000
43	1	0	6.693366	-2.830840	0.381214
44	6	0	5.260105	-2.450928	1.959254
45	1	0	4.180094	-2.320861	2.057193
46	1	0	5.564437	-3.273822	2.620168
47	7	0	2.544214	-0.060686	-0.691740
48	8	0	4.643620	2.055227	-1.529427
49	8	0	8.169879	1.110604	-1.745822
50	8	0	7.828351	0.449989	1.093594
51	8	0	5.841749	-1.221082	2.384315
52	8	0	5.107398	-1.829591	-0.396154
53	6	0	-5.108171	-1.271110	-0.254755
54	6	0	-4.034806	0.691823	0.090325
55	7	0	-5.236493	0.053212	0.069342
56	6	0	-6.351043	0.811353	0.295644
57	6	0	-7.544926	0.120539	0.233453
58	6	0	-7.506431	-1.268067	-0.066857
59	6	0	-6.314110	-1.969810	-0.316753
60	1	0	-8.489879	0.614340	0.398206
61	1	0	-6.345826	-3.022214	-0.558109
62	6	0	-4.374543	2.074665	0.361797
63	6	0	-5.812009	2.144721	0.500818
64	6	0	-3.620418	3.248193	0.480657
65	6	0	-6.444918	3.359937	0.768407
66	6	0	-5.669674	4.505480	0.890868
67	6	0	-4.273375	4.446103	0.742678
68	1	0	-3.692174	5.356189	0.833131
69	1	0	-7.522289	3.406775	0.873736
70	1	0	-2.545652	3.234851	0.360395
71	7	0	-3.792865	-1.531069	-0.436535
72	6	0	-3.117366	-0.355285	-0.229171
73	1	0	-6.144572	5.456839	1.097333
74	6	0	-8.768937	-2.052732	-0.143995
75	8	0	-8.823649	-3.240447	-0.397520
76	8	0	-9.856863	-1.304414	0.098234
77	6	0	-11.129420	-1.982109	0.043131
78	1	0	-11.871388	-1.218329	0.259622
79	1	0	-11.289624	-2.402410	-0.949619
80	1	0	-11.166356	-2.775405	0.789620

19·Ba(ClO_4)$_2$ (S_0) wB97XD

Center Number	Atomic Number	Atomic Type	Coordinates (Angstroms) X	Y	Z
1	6	0	-0.288031	2.346384	-1.472328
2	6	0	0.100259	3.142130	-0.392548
3	6	0	0.663112	2.062300	-2.449803
4	6	0	1.430624	3.465019	-0.196411
5	1	0	-0.623369	3.393147	0.372678
6	6	0	1.986651	2.431858	-2.287180
7	1	0	0.385988	1.463886	-3.309250
8	6	0	2.426716	3.015172	-1.086229

9	1	0	1.691268	3.990791	0.710527
10	1	0	2.684246	2.166182	-3.064986
11	6	0	3.277397	-3.343218	1.197705
12	1	0	3.616215	-3.197337	2.233099
13	1	0	3.865467	-4.160782	0.759007
14	6	0	1.816284	-3.726720	1.129554
15	1	0	1.649251	-4.644323	1.706495
16	1	0	1.541993	-3.903659	0.089455
17	6	0	0.685436	-2.619665	2.968594
18	1	0	0.771187	-3.610862	3.429620
19	1	0	-0.343968	-2.276022	3.047858
20	6	0	1.593241	-1.645526	3.697722
21	1	0	2.618513	-2.028037	3.802106
22	1	0	1.181628	-1.485009	4.702879
23	6	0	2.198300	0.636984	3.710660
24	1	0	3.208478	0.354515	4.043238
25	1	0	1.582621	0.865514	4.590325
26	6	0	2.257495	1.847374	2.805736
27	1	0	2.708329	2.688579	3.343139
28	1	0	1.244535	2.124535	2.507523
29	6	0	4.264966	2.154359	1.487430
30	1	0	4.929584	1.407703	1.056522
31	1	0	4.654850	2.453374	2.469321
32	6	0	4.216862	3.376635	0.569358
33	1	0	5.228370	3.793239	0.521076
34	1	0	3.596302	4.160280	1.006340
35	6	0	4.768517	2.545192	-1.696479
36	1	0	5.744094	2.805852	-1.280975
37	1	0	4.702952	3.053682	-2.666692
38	6	0	4.759896	1.037896	-1.984051
39	1	0	5.716627	0.815351	-2.480250
40	1	0	3.961615	0.730907	-2.662875
41	6	0	5.057577	-1.053828	-0.988858
42	1	0	4.497809	-1.514298	-1.809142
43	1	0	6.130097	-1.075407	-1.234974
44	6	0	4.845098	-1.850028	0.270686
45	1	0	5.413943	-2.782611	0.170388
46	1	0	5.226652	-1.305669	1.145629
47	7	0	3.776780	3.095580	-0.786324
48	8	0	2.998694	1.530949	1.634031
49	8	0	1.622044	-0.427412	2.982600
50	8	0	0.959161	-2.694462	1.578958
51	8	0	3.473925	-2.156751	0.451590
52	8	0	4.650005	0.286231	-0.793517
53	56	0	1.164543	-0.278641	0.133186
54	6	0	-2.838702	1.858089	-1.120492
55	6	0	-2.542085	-0.366796	-1.228249
56	6	0	-4.730929	0.613698	-0.650129
57	6	0	-3.064451	-1.650172	-1.059533
58	1	0	-2.447597	-2.530227	-1.178078
59	6	0	-5.254750	-0.641789	-0.480530
60	1	0	-6.276309	-0.793626	-0.165304
61	6	0	-4.403369	-1.760538	-0.697242
62	6	0	-5.061959	2.026441	-0.497150
63	6	0	-3.891228	2.791925	-0.784742
64	6	0	-6.243824	2.658822	-0.124514
65	1	0	-7.132607	2.079951	0.099438
66	6	0	-3.925643	4.181394	-0.694397
67	6	0	-5.112043	4.792255	-0.322382
68	1	0	-5.154329	5.873037	-0.246813
69	6	0	-6.260276	4.041475	-0.039740
70	1	0	-3.041204	4.769037	-0.911032
71	7	0	-3.428697	0.640292	-1.036739

72	1	0	-7.171584	4.550746	0.251351
73	7	0	-1.335248	0.183543	-1.463692
74	6	0	-1.509262	1.538773	-1.402043
75	6	0	-4.922726	-3.145522	-0.500283
76	8	0	-4.264956	-4.146589	-0.624899
77	8	0	-6.220779	-3.160840	-0.160482
78	6	0	-6.787760	-4.452345	0.056305
79	1	0	-7.828105	-4.274950	0.320517
80	1	0	-6.719873	-5.056771	-0.849695
81	1	0	-6.268782	-4.965926	0.867257
82	17	0	1.674032	-2.193731	-2.719855
83	17	0	-1.629162	0.391609	2.286719
84	8	0	2.799700	-3.129137	-2.758621
85	8	0	2.194723	-0.812119	-2.429715
86	8	0	0.781951	-2.522370	-1.567197
87	8	0	0.929926	-2.195054	-3.965409
88	8	0	-1.305892	-0.871002	1.562825
89	8	0	-0.670501	1.410966	1.751721
90	8	0	-1.410410	0.213213	3.723578
91	8	0	-2.997626	0.802013	2.001516

19·Ba(ClO$_4$)$_2$ (S$_1$) wB97XD

Center Number	Atomic Number	Atomic Type	Coordinates (Angstroms) X	Y	Z
1	6	0	-0.368426	2.475186	-1.322086
2	6	0	0.007083	3.208431	-0.187168
3	6	0	0.587005	2.292291	-2.327565
4	6	0	1.332887	3.551111	0.025808
5	1	0	-0.718324	3.387760	0.595399
6	6	0	1.905101	2.683499	-2.143247
7	1	0	0.317807	1.756979	-3.229236
8	6	0	2.338264	3.185391	-0.898411
9	1	0	1.589477	4.017927	0.964546
10	1	0	2.606258	2.494443	-2.939017
11	6	0	3.516740	-3.323247	0.977044
12	1	0	3.855573	-3.195775	2.013850
13	1	0	4.148334	-4.087376	0.504955
14	6	0	2.079447	-3.795503	0.902631
15	1	0	1.977693	-4.748327	1.434557
16	1	0	1.800208	-3.935651	-0.141146
17	6	0	0.866924	-2.880523	2.819296
18	1	0	0.993658	-3.898410	3.204028
19	1	0	-0.176652	-2.588473	2.909043
20	6	0	1.729843	-1.918875	3.620258
21	1	0	2.771885	-2.257306	3.697130
22	1	0	1.315194	-1.845492	4.633826
23	6	0	2.262081	0.397521	3.764418
24	1	0	3.304264	0.149590	4.012255
25	1	0	1.694057	0.518451	4.695660
26	6	0	2.187237	1.676462	2.955573
27	1	0	2.611583	2.505268	3.530733
28	1	0	1.145020	1.900331	2.729439
29	6	0	4.163797	2.134724	1.610213
30	1	0	4.819690	1.419633	1.119946
31	1	0	4.553646	2.350954	2.612403
32	6	0	4.122286	3.430723	0.793512
33	1	0	5.134644	3.846883	0.784747
34	1	0	3.496713	4.173981	1.288576
35	6	0	4.699953	2.821544	-1.545685

36	1	0	5.667803	3.084888	-1.115774
37	1	0	4.604288	3.401388	-2.471972
38	6	0	4.742387	1.342782	-1.956631
39	1	0	5.674091	1.208594	-2.526214
40	1	0	3.914408	1.054433	-2.605036
41	6	0	5.136778	-0.838204	-1.172796
42	1	0	4.525351	-1.217013	-1.994984
43	1	0	6.190471	-0.831098	-1.489065
44	6	0	5.008316	-1.756066	0.016156
45	1	0	5.563035	-2.672984	-0.215357
46	1	0	5.453091	-1.301133	0.911453
47	7	0	3.690326	3.266287	-0.589369
48	8	0	2.877575	1.512828	1.711205
49	8	0	1.694542	-0.647551	2.984131
50	8	0	1.153735	-2.842178	1.419565
51	8	0	3.643379	-2.092889	0.268697
52	8	0	4.745021	0.485576	-0.821853
53	56	0	1.157387	-0.358529	0.155087
54	6	0	-2.919030	1.922261	-1.028762
55	6	0	-2.579515	-0.308866	-1.209843
56	6	0	-4.803868	0.622642	-0.627794
57	6	0	-3.082611	-1.603549	-1.089246
58	1	0	-2.453006	-2.471005	-1.223522
59	6	0	-5.306034	-0.654156	-0.506281
60	1	0	-6.326929	-0.836403	-0.208671
61	6	0	-4.433012	-1.752252	-0.747529
62	6	0	-5.162811	2.020662	-0.426225
63	6	0	-3.991216	2.823834	-0.671977
64	6	0	-6.360100	2.623408	-0.044090
65	1	0	-7.242428	2.023611	0.146205
66	6	0	-4.054873	4.210533	-0.526707
67	6	0	-5.260045	4.787955	-0.145947
68	1	0	-5.321801	5.863753	-0.029545
69	6	0	-6.400991	4.005629	0.093637
70	1	0	-3.179479	4.822333	-0.708262
71	7	0	-3.491748	0.684811	-1.000587
72	1	0	-7.324281	4.487617	0.391897
73	7	0	-1.372540	0.278298	-1.402050
74	6	0	-1.569903	1.637069	-1.302241
75	6	0	-4.926981	-3.149285	-0.601598
76	8	0	-4.250614	-4.139999	-0.753656
77	8	0	-6.238199	-3.199459	-0.272753
78	6	0	-6.784196	-4.517769	-0.104180
79	1	0	-7.829933	-4.367763	0.154709
80	1	0	-6.693616	-5.088302	-1.029387
81	1	0	-6.261397	-5.046311	0.693955
82	17	0	1.732565	-1.977750	-2.852297
83	17	0	-1.632866	0.117351	2.289092
84	8	0	2.929309	-2.842321	-2.991287
85	8	0	2.176615	-0.574609	-2.444474
86	8	0	0.878311	-2.465667	-1.697289
87	8	0	0.952136	-1.922026	-4.094278
88	8	0	-1.240104	-1.134499	1.534580
89	8	0	-0.686211	1.197084	1.787130
90	8	0	-1.434920	-0.086913	3.743253
91	8	0	-3.023865	0.488427	1.974072
